# Formation of oligopeptides in high yield under simple programmable conditions

**DOI:** 10.1038/ncomms9385

**Published:** 2015-10-07

**Authors:** Marc Rodriguez-Garcia, Andrew J. Surman, Geoffrey J.T. Cooper, Irene Suárez-Marina, Zied Hosni, Michael P. Lee, Leroy Cronin

**Affiliations:** 1 WestCHEM, School of Chemistry, University of Glasgow, University Avenue, Glasgow G12 8QQ, UK

## Abstract

Many high-yielding reactions for forming peptide bonds have been developed but these are complex, requiring activated amino-acid precursors and heterogeneous supports. Herein we demonstrate the programmable one-pot dehydration–hydration condensation of amino acids forming oligopeptide chains in around 50% yield. A digital recursive reactor system was developed to investigate this process, performing these reactions with control over parameters such as temperature, number of cycles, cycle duration, initial monomer concentration and initial pH. Glycine oligopeptides up to 20 amino acids long were formed with very high monomer-to-oligomer conversion, and the majority of these products comprised three amino acid residues or more. Having established the formation of glycine homo-oligopeptides, we then demonstrated the co-condensation of glycine with eight other amino acids (Ala, Asp, Glu, His, Lys, Pro, Thr and Val), incorporating a range of side-chain functionality.

## Abstract (web summary)

Typically, in order to form large peptides, complex procedures or activating agents are required. Here, the authors show that simple aqueous conditions with dehydration-hydration cycles are sufficient for the oligomerization of amino acids into peptides (up to 20 amino acids long) in yields of up to 50%.

## Graphical abstract

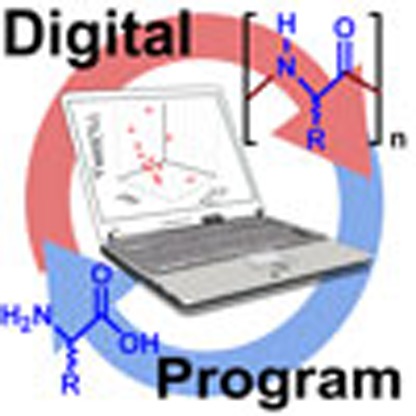

The peptide bonds that link together amino acids into short oligomers and proteins are vital for biology^1^. Because of this biological importance, many high-yielding reactions for forming peptide bonds have been developed, but these typically require activated amino-acid precursors and heterogeneous supports^2^. The formation of peptides from unactivated amino acids is hindered by thermodynamic constraints^3^, thermal decomposition if heated^4^ and requires a catalyst if yields higher than *ca*. 1% are to be achieved^5^,^6^,^7^. In addition, the formation of peptide bonds between amino acid monomers and the emergence of sequence bias is crucial to understanding the emergence of life^8^,^9^.

Although peptide bond formation has been widely established, obtaining oligopeptides in high yields requires catalysis, to overcome kinetic limitations, and activation of the amino acid monomers, to overcome thermodynamic limitations. Without these, studies have explored peptide synthesis on clays^10^, minerals^11^, at air–water interfaces^12^, on metal oxide surfaces^13^ and under hydrothermal conditions^14^,^15^, resulting in very low yields (typically <1%) of oligomeric products (where *n*>3). The difficulty arises as the condensation of amino acid monomers to form peptide bonds in aqueous solution (Fig. 1) is hampered by both unfavourable kinetics and thermodynamics, that is, the formation of peptide bonds is slow, and in aqueous solution the reagents are thermodynamically more stable than the peptide products.

In this work, we demonstrate an uncatalysed, amino acid oligomerization reaction producing unprecedentedly high yields of long oligomers using a very simple approach with a programmable reactor system allowing the exploration of many experimental parameters (see Supplementary Figs 1–6).

## Results

### The abiotic peptide synthesizer system

**Figure 2:**
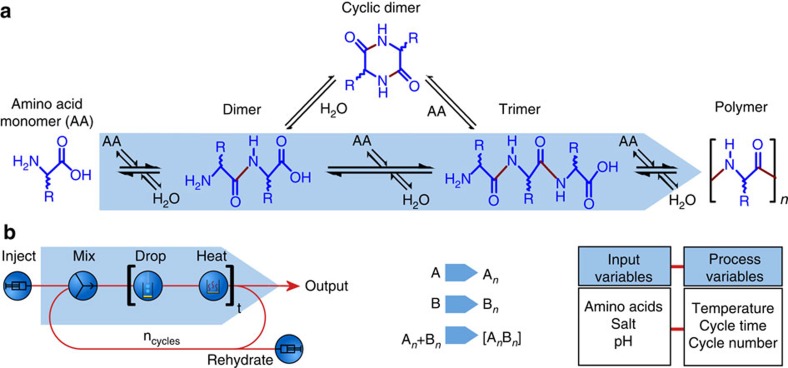
The APS. (**a**) Schematic of peptide bond formation to give oligomers from amino acid monomers. (**b**) Process flow scheme for the APS system: amino acid starting material is added and dehydrated for a given time (*t*), then rehydrated/dehydrated for a given number of cycles (*n*), after which the product outputs are removed for analysis. Single amino acid monomers (A, B and so on) may be added or, alternatively, defined amino acid 2-3-mer fragments (A_*n*_ and B_*n*_) can be used as starting materials. Reaction outcome is controlled by selection of input variables and process variables, and the overall reaction time is simply the time per cycle × the number of cycles.

In this work, we set out to explore the formation of peptide oligomers under the simplest possible ‘one-pot' reaction conditions. To do this, aqueous solutions of amino acid monomers were added to a hot empty glass vial and the water was removed by continuous heating. For subsequent dehydration–hydration cycles, further amounts of water were added to the reaction vials and were again allowed to completely evaporate. By taking this approach, we aimed to explore the formation of peptide bonds with minimal chemical inputs, see Fig. 2. Our investigations showed that even the simplest process parameters (that is, just a single addition–dehydration step) give high conversion of amino acid monomers to peptide oligomers. As we wished to investigate many different combinations of conditions (temperature, concentration of starting materials, pH and number of dehydration–hydration cycles), we designed the ‘abiotic peptide synthesiser' (APS), a computer-controlled reaction system that allowed us to run several reactions in parallel (see Fig. 2b), and automatically vary both the input and process variables.

A typical reaction run involved injection (10 ml min^−1^) of an aqueous solution of glycine (4ml, 0.0875M) containing NaCl (0.25 M), pH adjusted with NaOH (to 9.8), into a pre-heated vial (*T*=90 °C–130 °C), which was then maintained at that temperature for ∼15 h, evaporating the solution to dryness (the ‘dehydration step'). Subsequent cycles began with rehydration of the sample with 4 ml of water (the ‘hydration step') and proceeded similarly; after the final cycle, the vial was allowed to cool to room temperature. To prepare a solution for analysis, 8 ml of water was then added; typically, some insoluble precipitate is also observed, which was analysed separately. After only one dehydration–hydration cycle we can already observe oligomers in solution (up to Gly_12_) in up to around 50% yield by ion-pairing high performance liquid chromatography (IP-HPLC, see Methods section and Supplementary Figs 7–22)^16^ along with some cyclic glycine dimer (diketopiperazine DKP, not included in yields). Although our reaction conditions incorporate some NaCl (to maintain ionic strength at a similar order of magnitude in the absence of strong base to adjust pH), these conditions are very different to many previous studies (where often [NaCl]>>[Gly])^6^. Furthermore, our observations suggest that NaCl can be omitted, without significant loss of yield.

### Analysis of the products

Size exclusion chromatography–mass spectrometry (SEC-MS) was used to further confirm the identity of the oligomeric products and the presence of peptide bonds, revealing a range of oligomers that yielded tandem mass spectrometry (MS/MS) fragmentation patterns consistent with a peptide structure, see Fig. 3a,b (see Supplementary Figs 23–41). This was further corroborated by infrared analysis of precipitate (consistent with oligomer standards, see Supplementary Figs 42 and 43, and Fig. 3c,d), proton nuclear magnetic resonance spectroscopy (^1^H–NMR, see Supplementary Figs 44 and 45) and Biuret assay (a test for peptide oligomers, see Supplementary Figs 46–48).

### Exploring the parameter space

**Figure 4:**
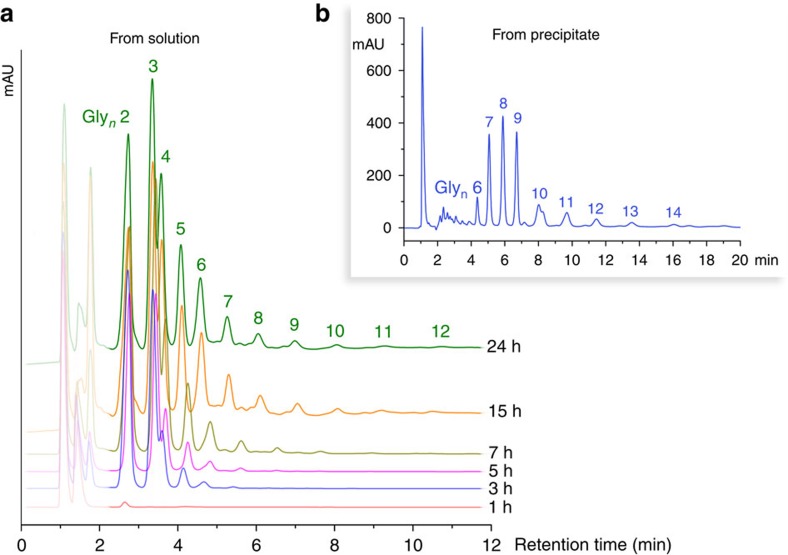
IP-HPLC analyses of products. (**a**) IP-HPLC chromatograms of a standard glycine condensation reaction, showing product oligomer length increasing with dehydration time (between 1 and 24 h). (**b**) Similar analysis of the solid precipitate formed during the reaction after two dehydration–hydration cycles, taken up in a 0.1% trifluoroacetic acid (TFA)/H_2_O solution, showing longer (sparingly soluble) oligomers. In both **a** and **b**, Gly_*n*_ oligomer peaks are labelled with the corresponding value of *n*; assignment of the peaks was established by comparison with Gly standards and by separate MS confirmation.

One important aspect of this work was the development of a robotic protocol to explore the parameter space for peptide bond formation. To do this, we constructed a robotic system to automatically search the parameter space. This is because we have previously found that automation and feedback can allow even simple systems to exhibit interesting outcomes^17^. To establish a feasible range of dehydration times for glycine oligomer formation, APS reactions were carried with dehydration times between 1 and 24 h. IP-HPLC analyses of the resulting soluble products showed that longer reaction times led to progressively longer oligomer products; products up to Gly_12_ could be observed after a single dehydration–hydration cycle (Fig. 4). Furthermore, significant quantities of insoluble products were observed in some of these reactions. To produce a solution amenable to IP-HPLC analysis, these precipitates were washed with an aqueous solution of 0.1% v/v trifluoroacetic acid; although not dissolving all the material, it was possible to observe larger oligomeric glycine species in the fraction which was dissolved, and this comprised mostly of higher oligomers (*n*>5, see Fig. 4b). Although not a quantitative technique, matrix-assisted laser desorption/ionization analysis of these insoluble fractions yielded evidence of oligomers>20-mer (see Supplementary Figs 40 and 41).

**Figure 5:**
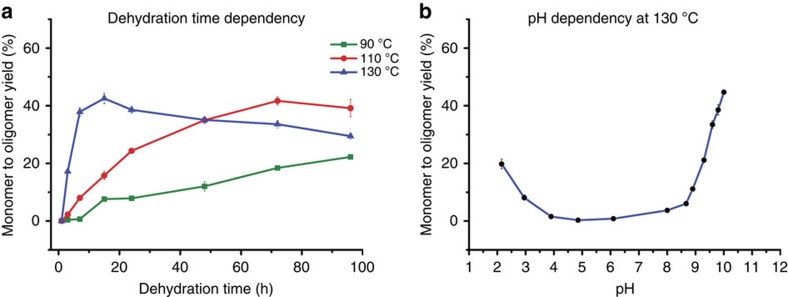
Exploring the parameter space. Graphs showing oligomer yield from a single cycle as a function of (**a**) dehydration time and (**b**) pH (at 130 °C for 24 h) (see Supplementary Figs 9 and 10 and Supplementary Tables 1 and 2).

Having demonstrated unactivated glycine oligomerization, we used the APS to systematically investigate the influence of other variables such as temperature, pH, number of cycles and cycle duration on the distribution of the oligopeptides formed (see Fig. 5). On raising the temperature of reaction from 90 °C to 130 °C, we observed a general increase in yield (from <1% after 1 h at 90 °C to *ca*. 50% after 15 h at 130 °C; see Fig. 5a). However, later in reactions at higher temperatures, we also observed the appearance of a brown colour (from a colourless solution; see Supplementary Fig. 11) and it is noteworthy that this coincides with an apparent drop in solution yields. This may be due to both precipitation of longer oligomer products or decomposition. Both are possible, but we note that no significant new peaks are resolved in IP-HPLC for decomposition products. Setting the pH of the amino acid input solutions was found to influence the reaction (see Fig. 5b); we observed a <0.1% yield at pH 6.1, rising to *ca.* 45% yield at pH 9.75 in more basic conditions, and to *ca.* 20% in more acidic conditions.

We also found that the formation of oligopeptides is possible over a monomer concentration range of 10^−4^ to 10^−1^ M (see Supplementary Fig. 15 and Supplementary Table 4). As the number of cycles increases the distribution of oligomer chain lengths observed in solution shifts. The yield of the lower oligomers decreases, whereas that of the longer oligomers in solution remains fixed and the amount of solid material precipitated increases (see Supplementary Fig. 14 and Supplementary Table 3). The yields achieved by the process described above are considerably in excess of those previously reported to result from similar reactions^6^; this is probably the result of exploring the effects of several parameters at once, without the constraint of hypotheses on the nature of optimum conditions. Although the system under consideration is more complex than might previously have been imagined (and mechanistic explanation of all the variance in reactivity observed is beyond the scope of this communication), we note that the starting pH is of particular importance (Fig. 5b and Supplementary Figs 12 and 13), with unprecedented yields observed from acidic and basic reaction mixtures. This is readily rationalized mechanistically: it has previously been shown that glycine dimerization proceeds most readily at high pH (ref. 18) where unprotonated amine groups are more nucleophilic, and hence more readily attack partner carbonyls, whereas at neutral pH glycine monomers are zwitterionic and interactions between the charged amino group and a charged carboxyl group reduce reactivity. The oligomerization of glycine under acidic conditions has not attracted particular attention because of the poor nucleophilicity of protonated amines; however, we note that acid catalysis should not be unexpected, as the OH group of the carboxylate becomes a better leaving group (H_2_O) on protonation and equilibria supplying a small amount of deprotonated amines should always be operative.

### Making and breaking peptide bonds

In addition to the oligomerization of glycine, we found that reaction of both glycinamide and DKP produce reasonable yields (>10%) at 130 °C, giving oligomers up to 10-mer (see Supplementary Figs 16 and 17). The role of DKP has been subject to debate: either seen as a ‘dead end'/thermodynamic ‘sink'^19^ or as able to react (often with caveats such as the presence of ‘free' amines)^15^. Observation of condensation of these amides and of possible traces of glycine monomer in DKP reactions lead us to pose an important question: are peptide bonds being concurrently made and broken during this process? To test this, we studied the reaction of linear Gly_2_ dimers under the same conditions. Along with the series of event-numbered Gly_*x*_ oligomers expected if only bond formation was occurring, we observed the formation of an odd-numbered series (*x*=3, 5, 7, etc.; see Supplementary Figs 19 and 20). This provides clear evidence that peptide bonds are both being formed and being broken concurrently, raising the possibility of dynamic combinatorial processes.

### Heteropolymers and expansion to other amino acids

Having established the formation of glycine homo-oligopeptides, we made some exploration of the co-condensation of glycine with several other amino acids (Ala, Asp, Glu, His, Lys, Pro, Thr and Val). In all of these cases, reverse-phase (RP)-HPLC-MS analysis revealed the presence of many species consistent with (Gly-X)_*n*_ hetero-oligomers and, furthermore, MS/MS analysis of representative masses (consistent with Gly_2_X_2_ tetramers) yielded *α*- and *y*-series fragments predicted by theory for such peptide structures (see Supplementary Figs 49–64). Furthermore, combination of Gly, Ala and Lys also resulted similarly in apparent formation of hetero-oligomers containing residues of all three amino acids (see Supplementary Figs 65 and 66). We note that the amino acids included in this preliminary investigation of co-oligomerization incorporate a wide range of functionality in their side chains: carboxylic acid (Glu and Asp), primary (Lys), secondary (Pro) and aromatic (His) amines, alcohol (Thr) and hydrophobic (Ala, Val) groups. Although fragments corresponding to linear peptide structures were observed, we also note that both branched structures and other forms of bond (for example, ester) may have been formed; indeed, given the presence of reactive side chains, this is not unlikely.

## Discussion

Although we have used an automated reaction system to discover and optimize the initial conditions for dehydration–hydration-driven peptide bond formation, this simple reaction does not require automation. At its most straightforward, the reaction of a 0.0875-M aqueous solution of G monomers (pH 9.8) over a period of 15 h at 130 °C in a simple glass vial was observed to produce oligomers of a length greater than the minimum previously shown to yield function (*n*≥7)^20^. These conditions are strikingly similar to the formation of ‘proteinoids' reported by Fox and Harada^21^,^22^. Although previous authors have reported formation of large insoluble products by such approaches^23^,^24^, it has been suggested that peptide bonds may have not been present in these products^25^,^26^; however, in light of our results it appears that such bond formation may have been possible. Such a simple procedure should be of interest to both synthetic chemists and to those interested in how recursive chemistry could allow the gradual emergence of order without human intervention^27^. In addition, it is worth mentioning that this process, involving reaction of an unadulterated solution of glycine in water over natrolite mineral, also gave peptide oligomers up to 8-mer (see Supplementary Figs 67 and 68) in only one cycle.

In this study, our investigation of the reaction input and process variables reveals that peptide bond formation from unactivated amino acids is less challenging than previously imagined. This process is both simple and general, does not require catalysts or activating reagents and can produce large yields of oligomers, for the majority of which *n*>3. Preliminary tests suggest that a wide range of amino acids may be co-condensed in a similar manner. We intend to continue to use this approach, to investigate the propensity of small biases (intrinsic reactivity, process variables and recursive processing), to influence the structures and sequences resulting from recursive reactions in fluctuating conditions. These aspects, not typically addressed when reporting uncontrolled peptide synthesis, are vital if such reactions are to be of use, whether for synthesis or the understanding of prebiotic peptide formation.

## Methods

### Apparatus

The synthesis took place in Bespoke automated apparatus comprising a set of programmable syringe pumps (C3000 Tricontinent), which were employed to flow the solutions to the heated reaction vessels. The pumps were controlled employing in-house developed LabView applications. Standard PEEK fittings were used to connect the tubing (1/16′′ OD, 0.3 mm ID) and the reaction vessels. Custom three-dimensional printed caps/fittings were manufactured for the reaction vessels. Two different contact hotplates (RCT basic, IKA) fitted with DrySyn heat-transfer blocks were used in parallel, to perform an array of experiments under different temperature conditions (see Supplementary Figs 1 and 2).

### Operation and programme

The unit operations of each cycle are controlled by LabView. We designed a programme capable of controlling the flow rate of addition, volume and cycle time of each individual experiment within a continuous loop (see Supplementary Figs 3–6 and Supplementary Methods). Each one of these values can be easily modified between experiments, as well as the total number of cycles. Before starting the experiment, the desired values were entered in the programme and the experiments were left running autonomously over a specific period of time (depending on the number of cycles).

A typical peptide synthesis experiment involved the following: (a) the preparation of a dilute solution of starting material was performed by taking an aliquot of 350 μl from a 1M solution of glycine, to which we subsequently added 1 ml of a 1M NaCl solution and 2.55 ml of (HPLC) water, and finally the pH was adjusted to 9.8 by adding 100 μl of 1 M NaOH. (b) Glass reaction vessels were placed in the corresponding Drysyn hotplate inserts. Custom-made three-dimensional printed polypropylene lids with integrated holes were placed on each vial to connect them to the pumps, which facilitated the evaporation during the drying step. Water solutions were connected to each individual pump to deliver a given volume in each re-hydrating step. (c) Then, the prepared solution was injected in a pre-heated vial (*T*=130 °C). (d) The process inputs (volume, flow rate, dehydration time and number of cycles) were entered for each pump or set of pumps. The pumps were initialized and tested before starting the experiment, to ensure their correct functioning. Finally, the array of experiments was started by pressing the START button. (e) After initializing the programme, the vials were kept at 130 °C for a given time, to evaporate the solution to complete dryness. Once a cycle was finished, the process was restarted by rehydrating the sample with 4 ml of HPLC water. (f) Once finished, products were collected for analysis by adding 8 ml of a 0.1% trifluoroacetic acid aqueous solution. Then, 500 μl of the extracted sample were taken for HPLC analysis.

Although the reactions shown here were performed in glass vials, we note that the same series of oligomeric products were also observed if the reactions were run in Teflon reactors (see Supplementary Fig. 18). Although NaCl is present in these reactions, omission of NaCl in otherwise identical reactions resulted in no significant drop in yield (see Supplementary Information, Supplementary Fig. 21). Furthermore, although NaOH was used to adjust pH, reactions using LiOH instead show that Na^+^ is not crucial (see Supplementary Figs 22 and 23).

### Determination of soluble oligomer yields

The concentration of the smaller soluble oligomer products (1-mer to 6-mer; also DKP) was established by integration of absorbance values (195 nm) and calibration with commercially available standards. The calibration constants for larger oligomers were estimated based on the mean absorbance per glycine unit in the larger standards, which was observed to become approximately constant >3-mer (see Supplementary Figs 7 and 8).

The yield was then calculated as a proportion of glycine (or glycine oligomer) starting material input. The data are averaged over three repetitions and error bars show the s.d.

### IP-HPLC analysis

IP-HPLC analysis was performed using an Agilent 1100 HPLC system fitted with a reversed-phase C18 column (Phenomenex Luna, 300 × 7.8 mm). Samples were injected in 5 μl aliquots and eluted isocratically at 0.3 ml min^−1^ with a mobile phase consisting of 50 mM KH_2_PO_4_ and 7.2 mM C_6_H_13_SO_3_Na solution, adjusted to pH 2.5 using H_3_PO_4_. The oligomeric products were detected at 195 nm and the retention times confirmed by comparison with pre-made standards containing glycine monomer, glycine anhydride, as well as glycine peptide oligomers (*n*=2–6). The instrument was controlled and the resulting data analysed using Agilent Technologies OpenLAB Software.

### SEC-MS analysis

SEC-MS analyses were performed (also with an Agilent 1100) system fitted with a Polysep-GFC-P 1,000 column (Phenomenex, 300 × 7.8 mm) and PolySep-GFC-P guard column (Phenomenex, 35 × 7.8 mm). Samples were injected in 10 μl aliquots and eluted isocratically at 0.4 ml min^−1^ with 90% aqueous NH_4_OAc (50 mM) and 10% acetonitrile.

The MS apparatus was a Bruker MaXis Impact instrument, calibrated for the 50–1,200 Da range using sodium formate solution. The eluent stream was introduced directly into the source (no splitting) following the diode array detector, at a dry gas temperature of 200 °C. The ion polarity for all MS scans recorded was positive, with the voltage of the capillary tip set at 4,800 V, end plate offset at −500 V, funnel 1 radio frequency (RF) at 400 Vpp and funnel 2 RF at 400 Vpp, hexapole RF at 100 Vpp, ion energy 5.0 eV, collision energy at 5 eV, collision cell RF at 200 Vpp, transfer time at 100.0 μs and the pre-pulse storage time at 1.0 μs.

### Reverse-phase HPLC-MS

RP-HPLC analyses were performed (also with an Agilent 1200 series instrument) fitted with an Agilent Poroshell 120 EC-C18 (4.6 × 50 mm, 2.7 μm) column. Samples were injected in 10 μl aliquots (except when required to obtain higher signal intensity for some MS/MS experiments—all chromatograms obtained with comparable injection volumes) and eluted with a linear gradient mixture of solvents A (water w/0.1% v/v formic acid) and B (100% acetonitrile w/0.1% v/v formic acid) over 21 min as follows: 0 min, 100% A; 3 min, 100% A; 13 min,100% B; 15 min, 100% B; 18 min, 100% A. The column over was maintained at 30 °C.

The MS apparatus was a Bruker MaXis Impact instrument, calibrated for the 50–1,200 Da range using sodium formate solution. The eluent stream was introduced directly into the source (no splitting) following the diode array detector, at a dry gas temperature of 200 °C. The ion polarity for all MS scans recorded was positive, with the voltage of the capillary tip set at 4,800 V, end plate offset at −500 V, funnel 1 RF at 400 Vpp and funnel 2 RF at 400 Vpp, hexapole RF at 100 Vpp, ion energy 5.0 eV, collision energy at 5 eV, collision cell RF at 200 Vpp, transfer time at 100.0 μs and the pre-pulse storage time at 1.0 μs. In MS/MS experiments, collision-induced dissociation energies were optimized according to products (typically between 15 and 30 eV) and the quadrupole mass ‘window' was set as 0.01 Da.

### General MS control and data processing

All data were acquired using the Bruker MaXis Impact instrument, controlled by the Compass software suite; where chromatography analysis was added, this process was controlled by accompanying Bruker Hystar software (running Agilent ICF for instrument interface). Where extracted ion chromatograms (EICs) are presented, they were extracted from the raw data using Compass Data Analysis (*m/z* ±0.01, unless otherwise stated) and exported as *xy* data; they were then plotted using Origin 8.5.0 (applying Savitsk–Golay smoothing—points of window=5, polynomial order=2, normalizing intensity to a 0–1 scale for ready comparison). MS/MS spectra presented in this Supplementary Information document were exported from Compass Data Analysis plotted in R, and theoretically predicted fragmentation patterns (based on putative sequence assignment) were compared with experimentally derived spectra using the OrgMassSpecR library (*m*/*z* matching ±0.05; threshold intensity 3%)^29^ running in the R environment^30^. MS/MS spectra presented in the manuscript were exported from Compass Data Analysis, and underwent the same assignment process, however were plotted for publication using Origin 8.5.0.

### Generation of vBPC traces to demonstrate co-oligomerization

Observed virtual base peak chromatogram (vBPC) data for RP-HPLC-MS runs was produced to demonstrate co-oligomerization of amino acid species to form complex mixtures. These were constructed from a combination of all the observed EICs corresponding to the members of a list of possible oligomer products (*vide infra*). This was accomplished in the following steps (all operations in R using a custom script): (a) acquisition of RP-HPLC-MS data for a set of amino acid oligomerization products. (b)Conversion of the raw data files from Bruker proprietary format to centroided.mzXML format, using the MSConvert software^31^. (c) Generation of a combinatorial list of all the putative oligomeric products of the condensation reaction (that is, all combinations and sequence permutations) under R (using the OrgMassSpecR library for fragmentation pattern prediction and mass calculation), produced on the basis of the amino acids incorporated and a maximum length (chosen to ensure that all species up to the mass limit at which the data were acquired was accounted for). (d) Removal of homo-oligomer products (that is, all VBPCs represent only co-oligomerization of the starting species). (e) Generation of the putative of a mass list incorporating all the putative species. (f) Extraction of EIC data for each member of the mass list using xcms^32^ under R. (g) Generation of a vBPC from the EICs based on the highest intensity observed in the EIC of any member of the mass list and (h) plotting in R.

### Direct infusion mass spectrometry

Direct infusion MS analyses were performed using a Thermo Scientifc LTQ Orbitrap XL apparatus. The sample was solvated in water and diluted into a mixture of water:methanol (1: 1) with 10% formic acid before analysis by positive nanospray (see Supplementary Figs 29–39).

### MALDI analysis of a solid fraction

MALDI analyses were performed using a Voyager DE-STR MALDI-TOF with Linear and Reflectron analysers (equipped with an Nd:YAG laser (*λ*=355 nm)). Sample was attempted by solvent-free MALDI, initially with CHCA (α-Cyano-4-hydroxycinnamic acid) matrix typically used for peptide samples. However, no polymeric species and only matrix ions were observed. When the analysis was repeated with DHB (2,5-dihydroxybenzoic acid) matrix, four oligomeric series were observed in addition to matrix ions. The four series all show the expected repeat unit mass of 57 Da and correspond to [M+H]^+^, [M+Na]^+^, [M+K]^+^ and the MALDI artefact [M−H+2Na]^+^ (see Supplementary Figs 40 and 41).

### Transmission infrared spectroscopy

Transmission infrared spectroscopy was performed on samples in the solid phase using a Thermo Scientific Nicolet iS5 instrument with Specac Golden Gate attachment and processed with OMNIC software (see Supplementary Figs 42 and 43 and Supplementary Table 5).

### ^1^H-NMR spectrometry

^1^H-NMR spectra were recorded on a Bruker Avance III (500.2 MHz) using the deuterated solvent as the lock and the residual solvent as the reference. All spectra were run in D_2_O (see Supplementary Figs 44 and 45).

### Qualitative chemical testing for peptide bonds

Qualitative chemical testing for peptide bonds was performed using the Biuret test. One millilitre of Biuret reagent (hydrated copper (II) sulfate, sodium hydroxide (NaOH) and potassium sodium tartrate) was added to a solution of ∼5 mg amino acid/polyamino acid in 1 ml of fresh 0.1 M sodium hydroxide solution. A positive test for peptide bonds is shown by a colour change from pale blue to violet. Further quantitative analysis was performed (see Supplementary Table 6 and Supplementary Methods).

## Additional information

**How to cite this article:** Rodriguez-Garcia, M. *et al*. Formation of oligopeptides in high yield under simple programmable conditions. *Nat. Commun.* 6:8385 doi: 10.1038/ncomms9385 (2015).

## Supplementary Material

Supplementary Information Supplementary Figures 1-68, Supplementary Tables 1-6, Supplementary Methods and Supplementary References.

## Figures and Tables

**Figure 1:**
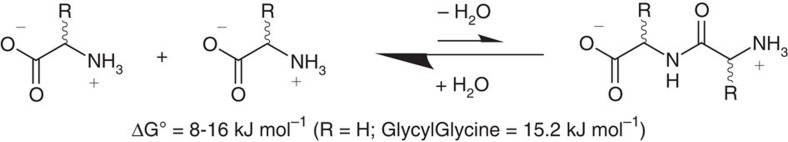
Peptide bond formation. Scheme showing equilibrium and ΔG^o^ for peptide bond formation in aqueous solution^28^.

**Figure 3:**
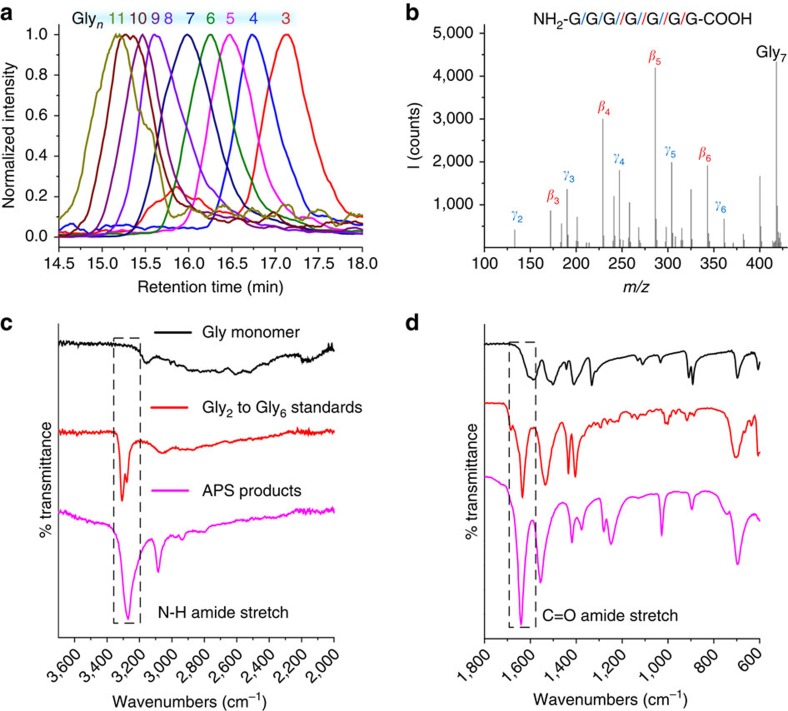
Product characterization. (**a**) Extracted ion chromatograms (EICs) for a series of glycine oligomers—from Gly_3_ to Gly_11_—produced in a typical monomeric glycine experiment on analysis by SEC-MS, see Methods sections for details. (**b**) Typical SEC-MS/MS spectrum of one such species (Gly_7_, *m/z*=418.17); the peptide structure is confirmed by fragmentation to *β*- and *γ*-series product ions of masses predicted by theory for collision induced dissociation (CID) of Gly_7_ (see Supplementary Figs 24–28). Infrared showing the comparison of glycine monomer, oligomer standards and the reaction products from the APS. (**c**) Infrared spectrum in the region 3,600–2,000 wavenumbers showing the N–H amide stretch and (**d**) infrared spectrum in the region of 1,800–600 wavenumbers showing the C=O amide stretch.
