# Supplementary material for: Formation of oligopeptides in high yield under simple programmable conditions
Source: Nat Commun. 2015 Oct 7;6:8385. doi: 10.1038/ncomms9385 (PMC4633627; doi:10.1038/ncomms9385)
Supplement: Supplementary Information — Supplementary Figures 1-68, Supplementary Tables 1-6, Supplementary Methods and Supplementary References. [file ncomms9385-s1.pdf]

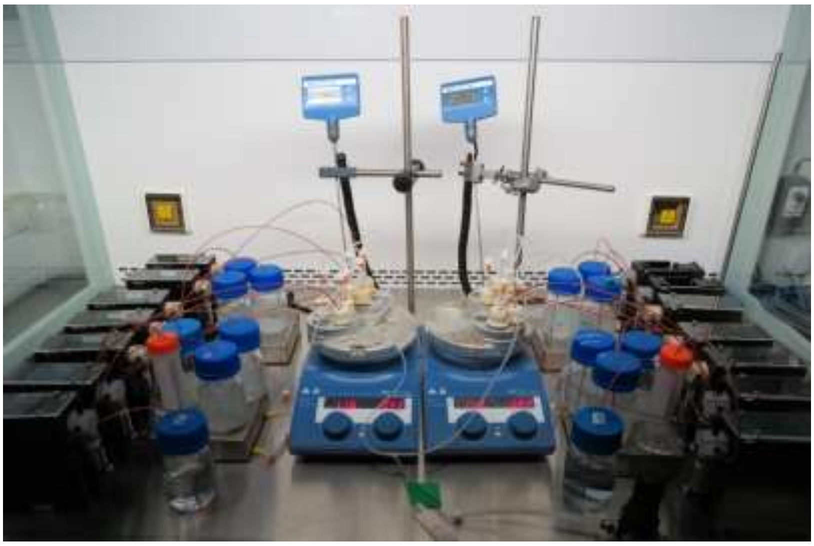

Supplementary Figure 1: Photograph of two APS systems in operation.

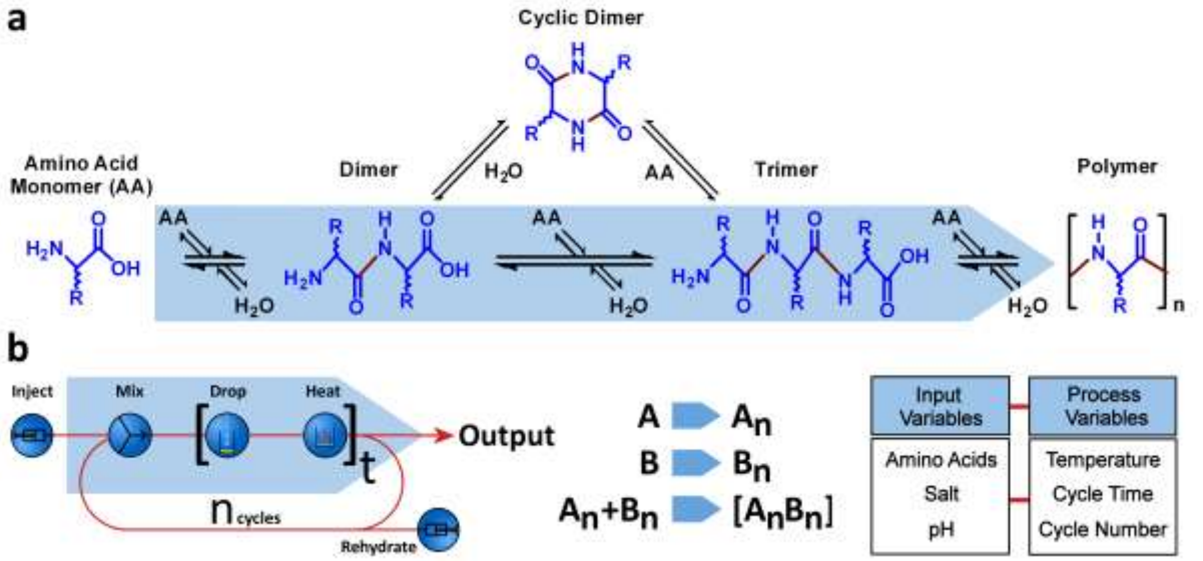

Supplementary Figure 2: a) Schematic of peptide bond formation to give oligomers from monomeric amino acids. b) Process flow scheme for the Abiotic Peptide Synthesiser (APS) system: amino acid starting material is added and dehydrated for a given time (t) then rehydrated / dehydrated for a given number of cycles (n), after which the product outputs are removed for analysis. Single amino acid monomers (A, B) may be added or, alternatively, mixed amino acid 2-4-mer fragments / higher order oligomers (A_n, B_n) can be used as starting

materials. Reaction outcome is controlled by selection of input variables and process variables.

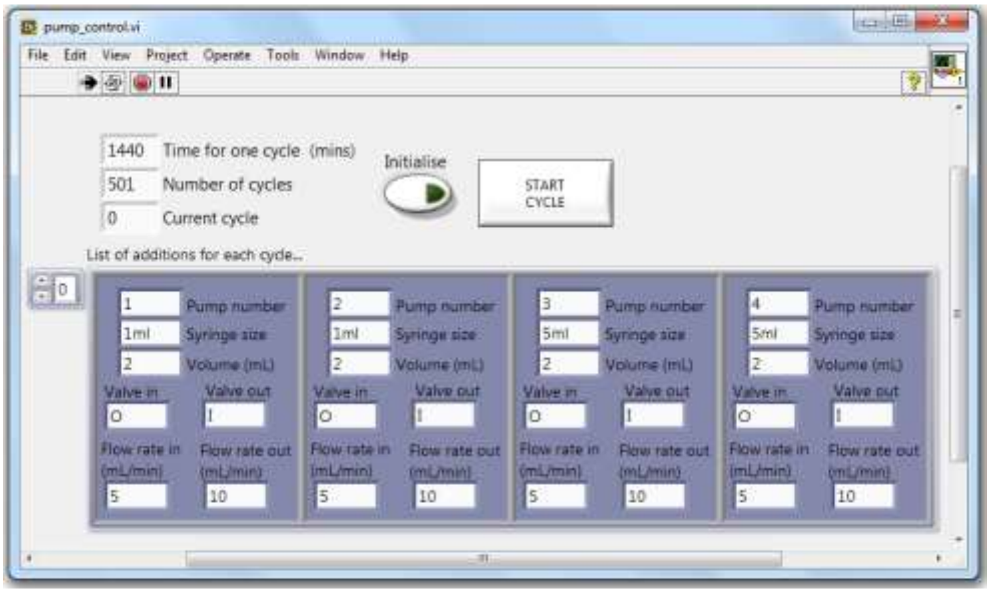

Supplementary Figure 3: A screen capture image of the National Instruments LabVIEW control software user interface. The software is designed specifically for the control of the experiment described in the manuscript, which features cyclic movement of fluids via Tricontinent syringe pumps. The “Time for one cycle,” and “Number of cycles,” are set by the user, and the software displays the “Current cycle,” once the experiment begins. Each pump instruction in the cycle is determined by an element in the array “List of additions for each cycle…”. Each element requires the user to input the pump number to be addressed (up to 16 pumps), the size of the syringe that that pump is equipped with, the Volume (ml) to be pumped, the valve positions (i.e. where to pump the fluid), and the speed at which to pump. Note that the Volume can be greater than the syringe size, in which case the pump performs the instruction in multiple steps. Shown in this image are the instructions for pumps 1 – 4, however further elements can be added or removed prior to starting the experiment. Once the controls are set correctly, “Start Cycle” begins the experiment. Alternatively, “Initialise” can be used to empty all syringes.

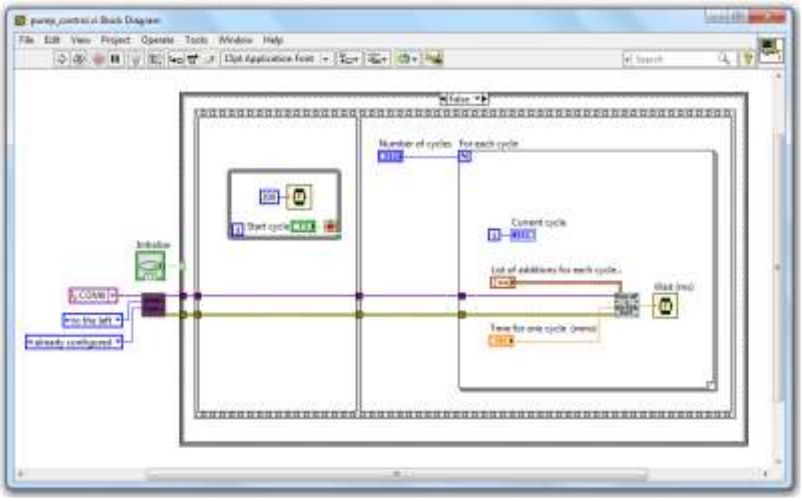

Supplementary Figure 4: The National Instruments LabVIEW software Block Diagram associated with the Front Panel in Fig. S3. The pump configuration is hard-wired into an initialisation Sub VI. Shown is the non-initialisation case, the software waits for the user to click “Start Cycle”. A “For” loop, named “For each cycle,” then runs for the “Number of Cycles,” set up the user. A Sub VI named “Pick up and Deliver (dist vale).vi,” reads the “List of additions for each cycle…,” and executes them in parallel, then outputs the remaining “Wait (ms)” time in milliseconds which is used to delay to next iteration of the experiment.

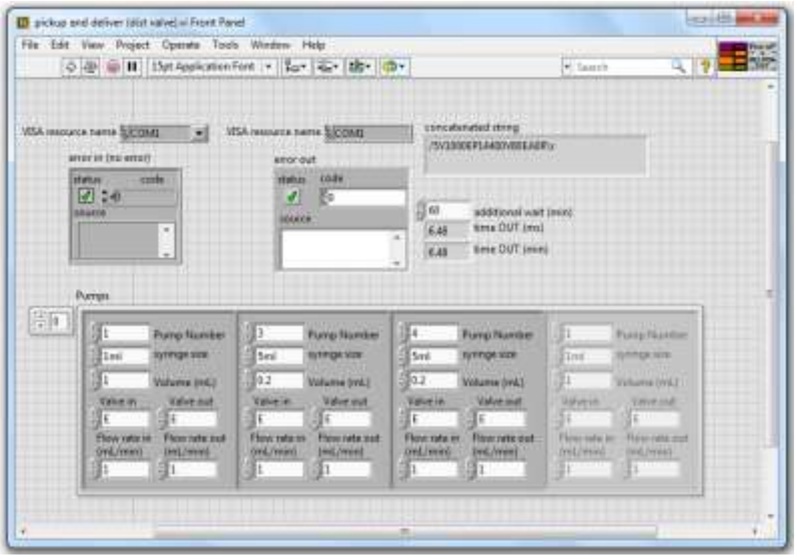

Supplementary Figure 5: A screen shot of the front panel of the “Pick up and Deliver (dist vale).vi” Sub VI as used by the “pump_control.vi”. The function of this VI is to convert the

user defined instructions in the “Pumps” array into a string command (named “concatenated string”) which is sent via serial to the each pump and valve unit.

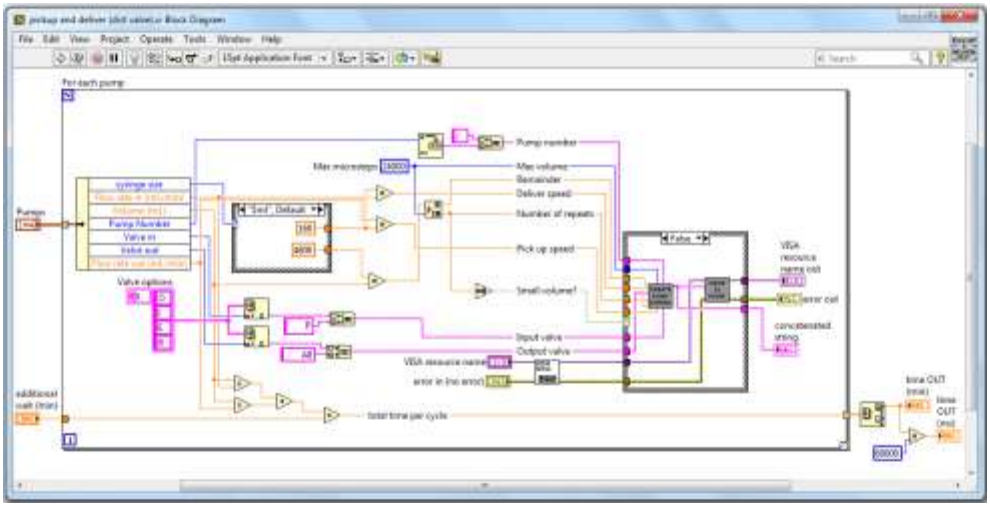

Supplementary Figure 6: The Block Diagram associated with the Front Panel in Supplementary Fig. 5. This VI converts the user instructions in the “Pumps” array (equal to “List of additions for each cycle…”) into a string command, and then sends that command to the correct pump. This is done in a “For” loop, named “For each pump,” whereby each pump is addressed sequentially (the order is determined by the order they appear in the “Pumps” array). The VI also calculates the time to wait between each iteration of the experiment, given by the sum of the time taken to operate the pumps, plus the user defined “additional wait (min)”.

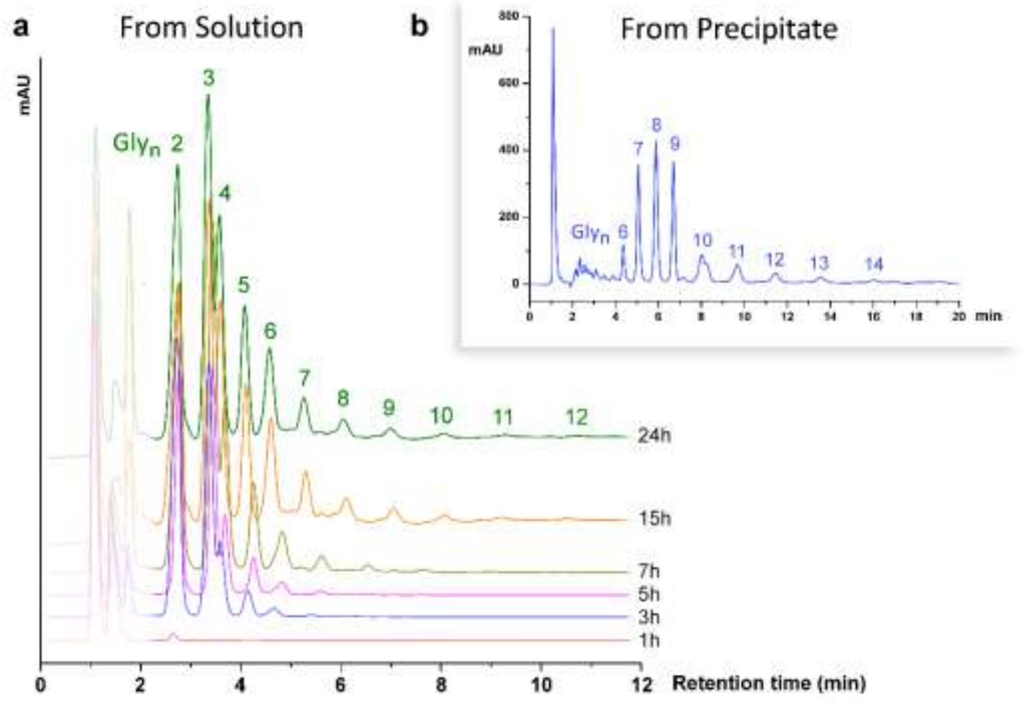

Supplementary Figure 7: **a**) Ion pairing HPLC chromatograms of a standard glycine condensation reaction, showing oligomer length increasing with dehydration time (between 1 and 24 hours). The traces prior to elution of $(Gly)_2$ are faded for clarity; this region includes peaks due to glycine monomer and cyclic glycine dimer (diketopiperazine - DKP) along with any ionic/polar contaminants and 'method peaks'. **b**) Similar analysis of the solid precipitate formed during the reaction taken up in a 0.1 % TFA/H_2O solution, showing longer (sparingly soluble) oligomers. In both (a) and (b), Gly_n oligomer peaks are labelled with the corresponding value of n; assignment of the peaks was established both by comparison to Gly standards, and by separate mass spec. confirmation of the presence of oligomers.

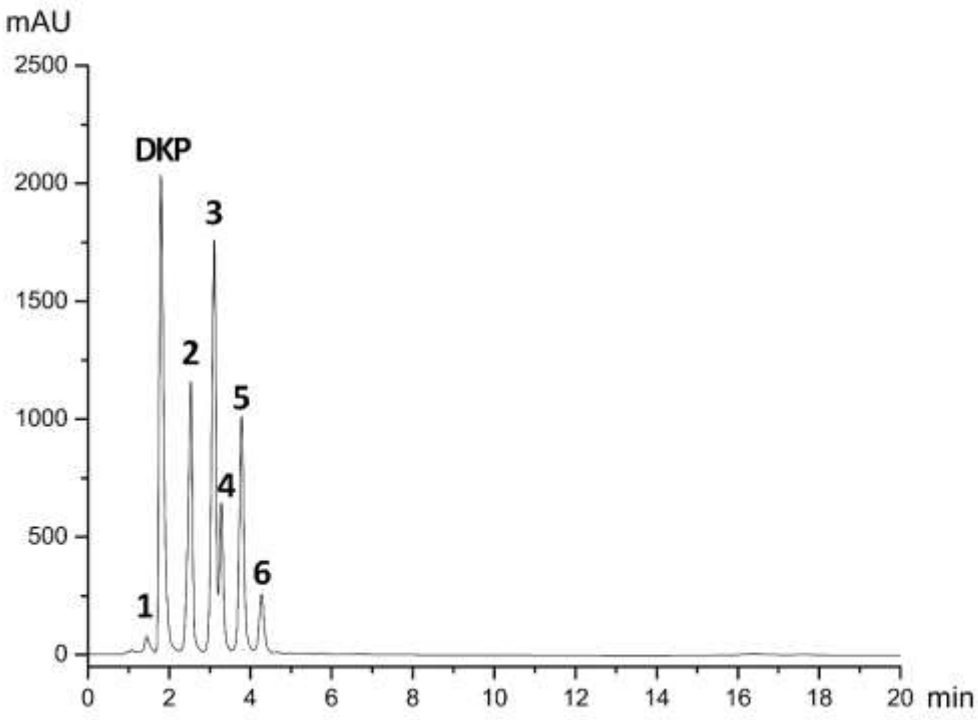

Supplementary Figure 8: IP-HPLC chromatogram of Gly_n oligomer standards.

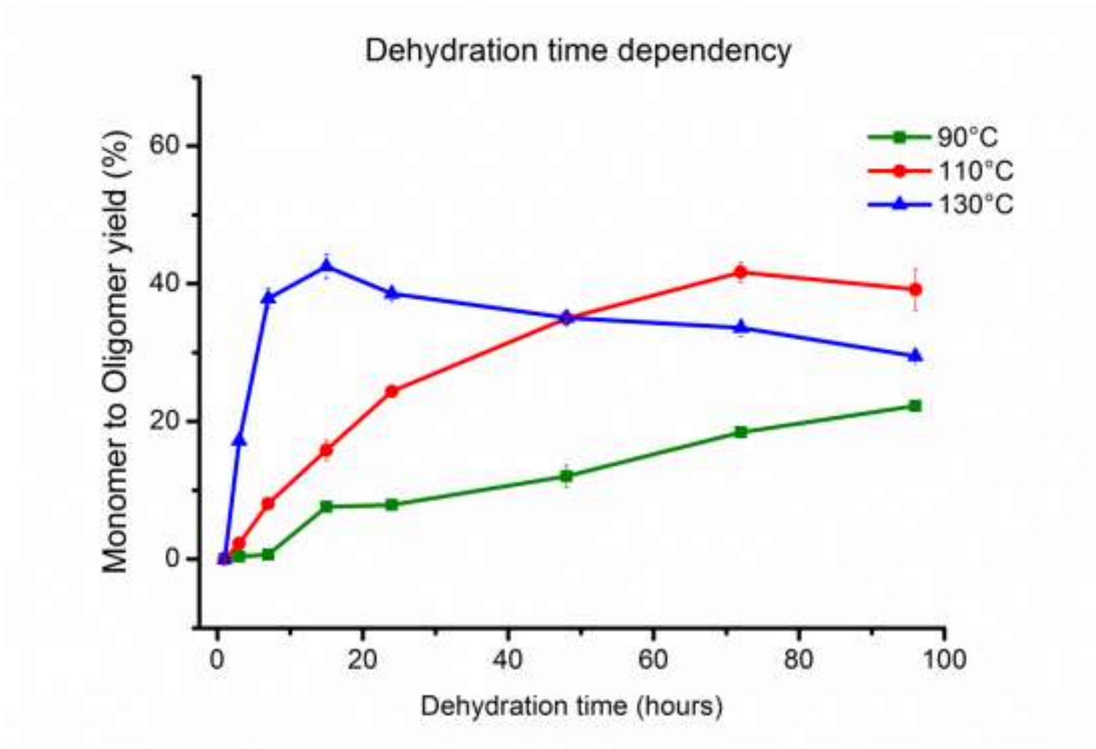

Supplementary Figure 9: Monomer to Oligomer yield (%) vs. dehydration time at 90ºC, 110ºC and 130ºC.

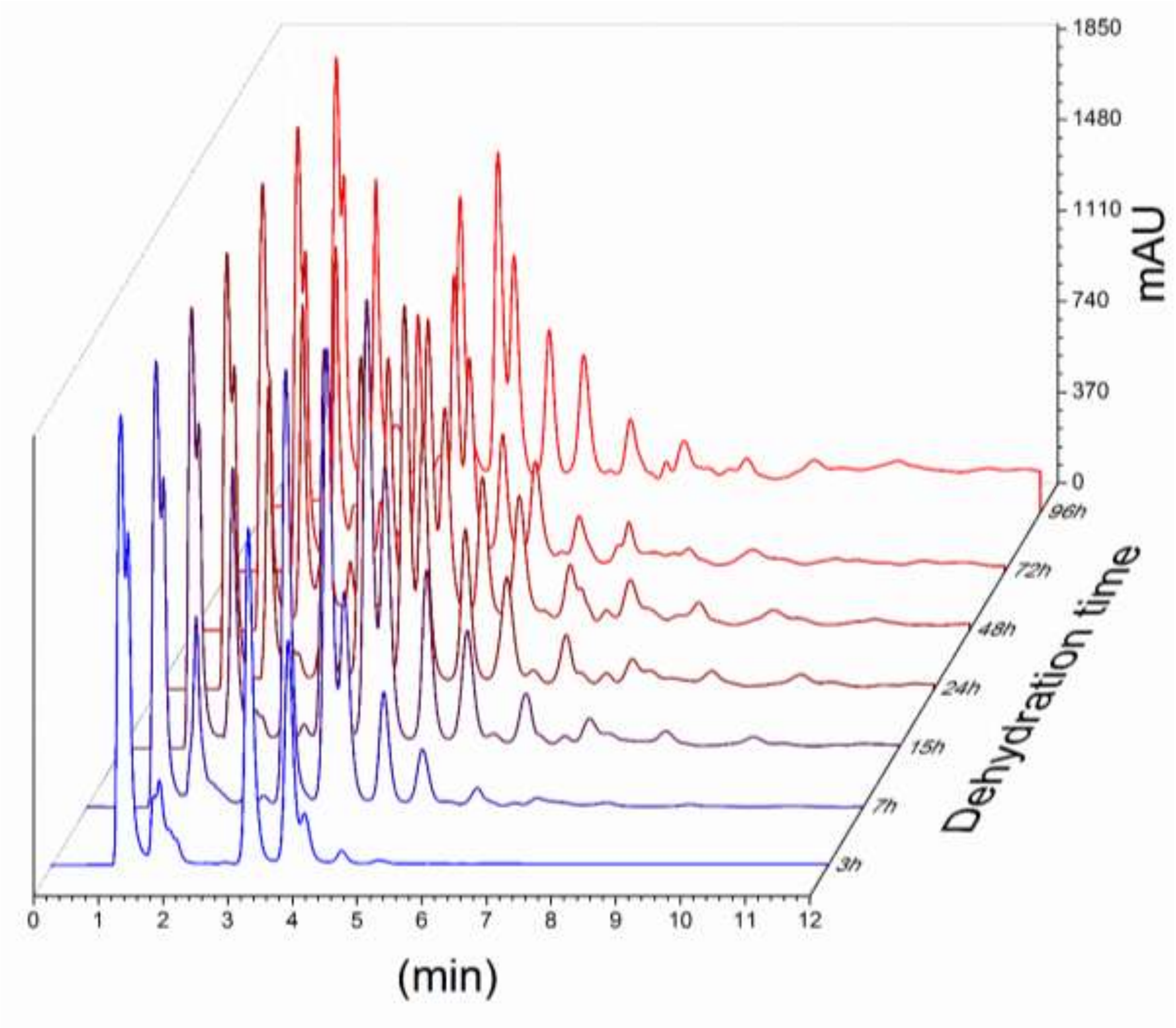

Supplementary Figure 10: IP-HPLC chromatograms showing the oligomerisation products of glycine as a function of the dehydration time at130ºC.

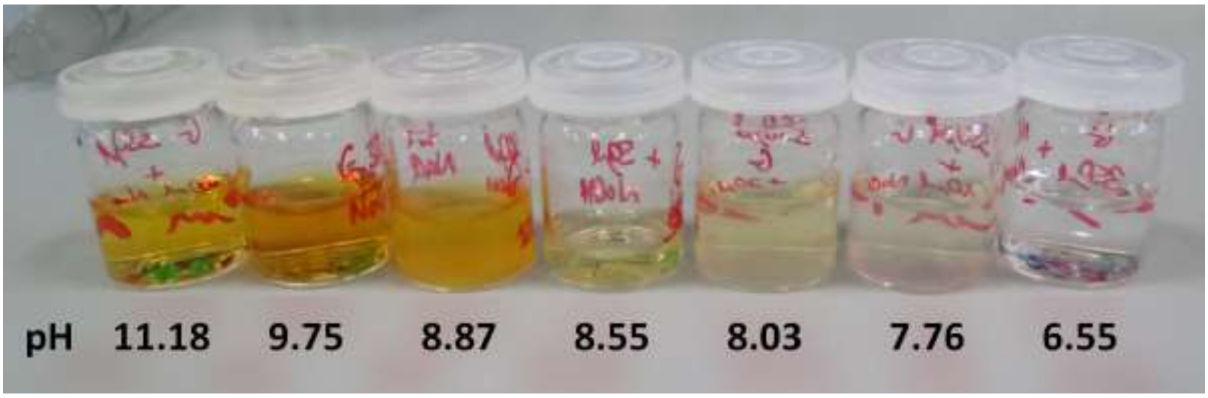

Supplementary Figure 11: Correlation between the pH and the browning produced by decomposition processes.

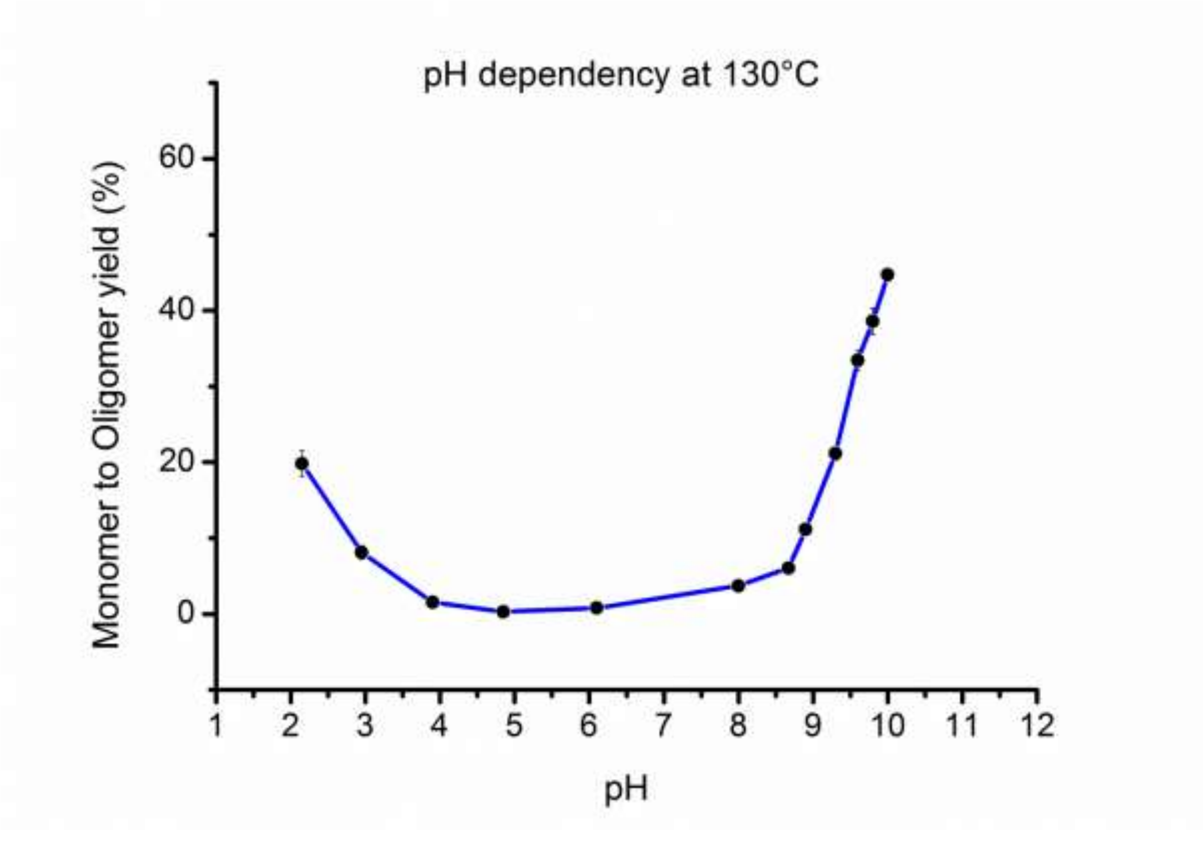

Supplementary Figure 12: Monomer to Oligomer yield (%) vs. pH at 130ºC for a single dehydration cycle (24h).

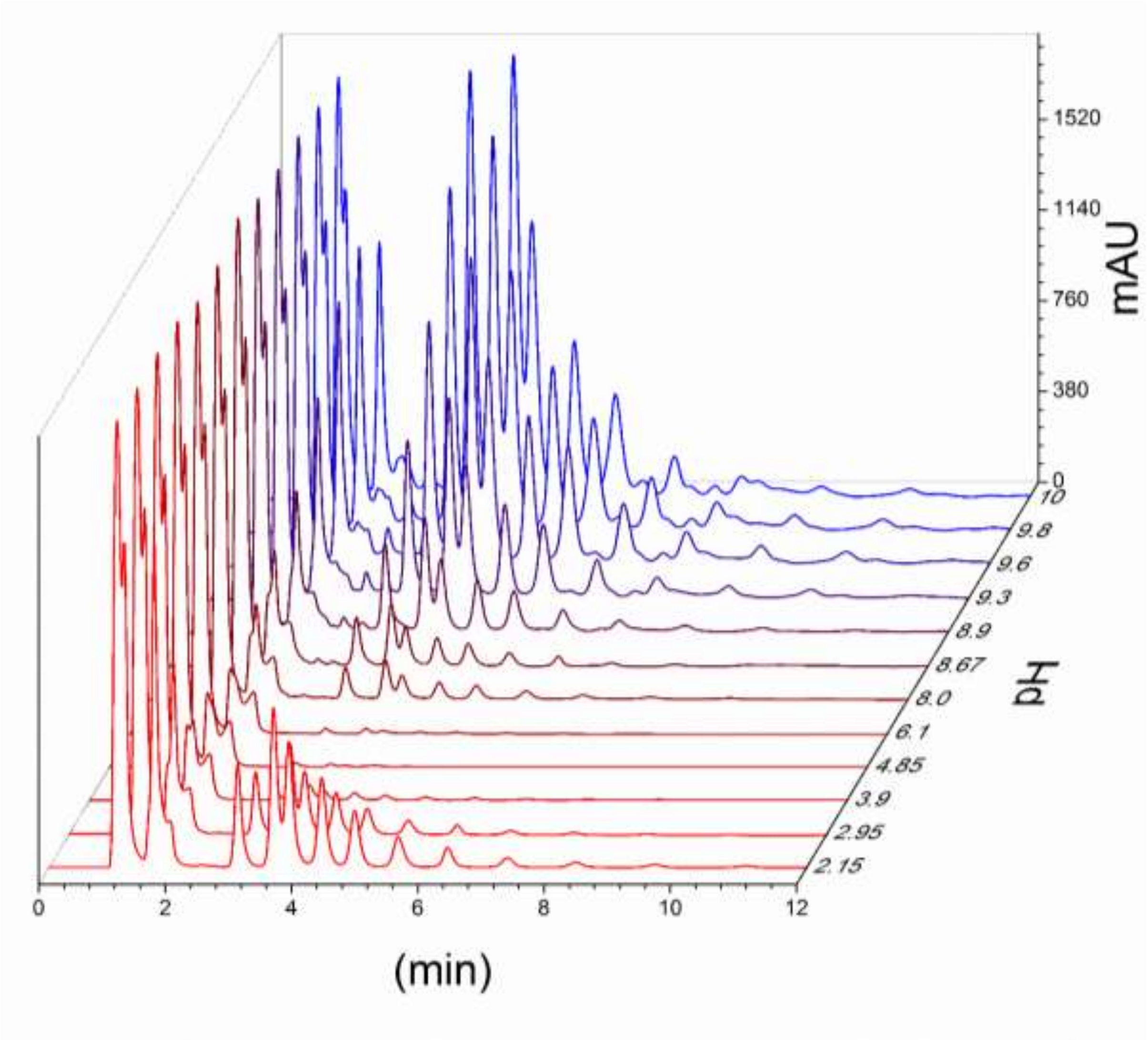

Supplementary Figure 13: IP-HPLC chromatograms showing the oligomerisation products of glycine as a function of the pH in a single dehydration cycle (24h) at 130ºC.

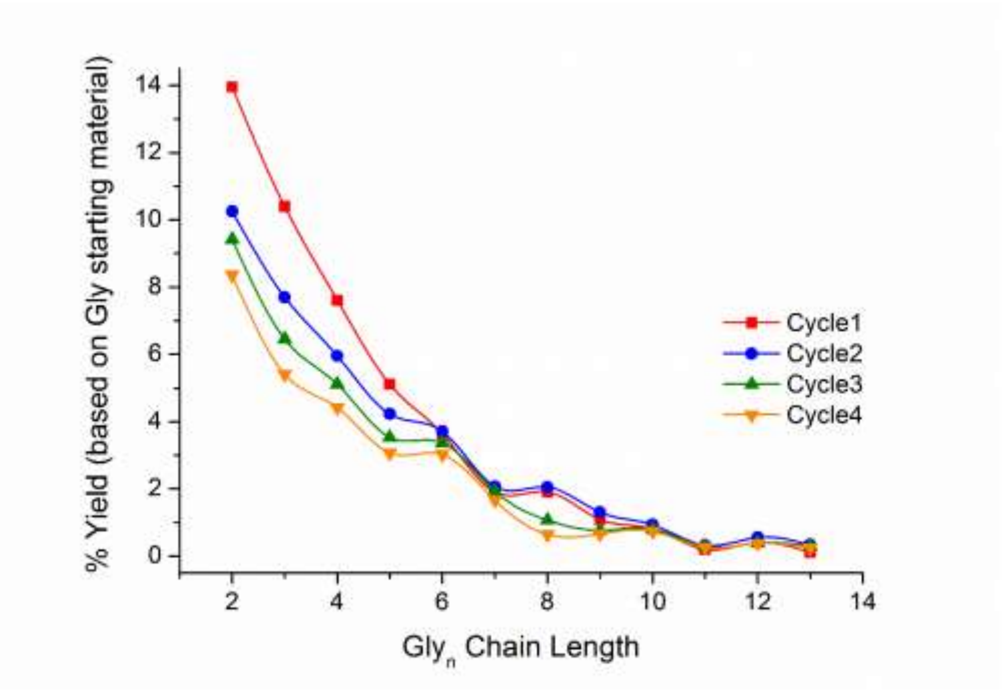

Supplementary Figure 14: Distribution of Gly_n chain lengths as a function of dehydration cycle number (oligomer yields expressed as % of initial Gly added).

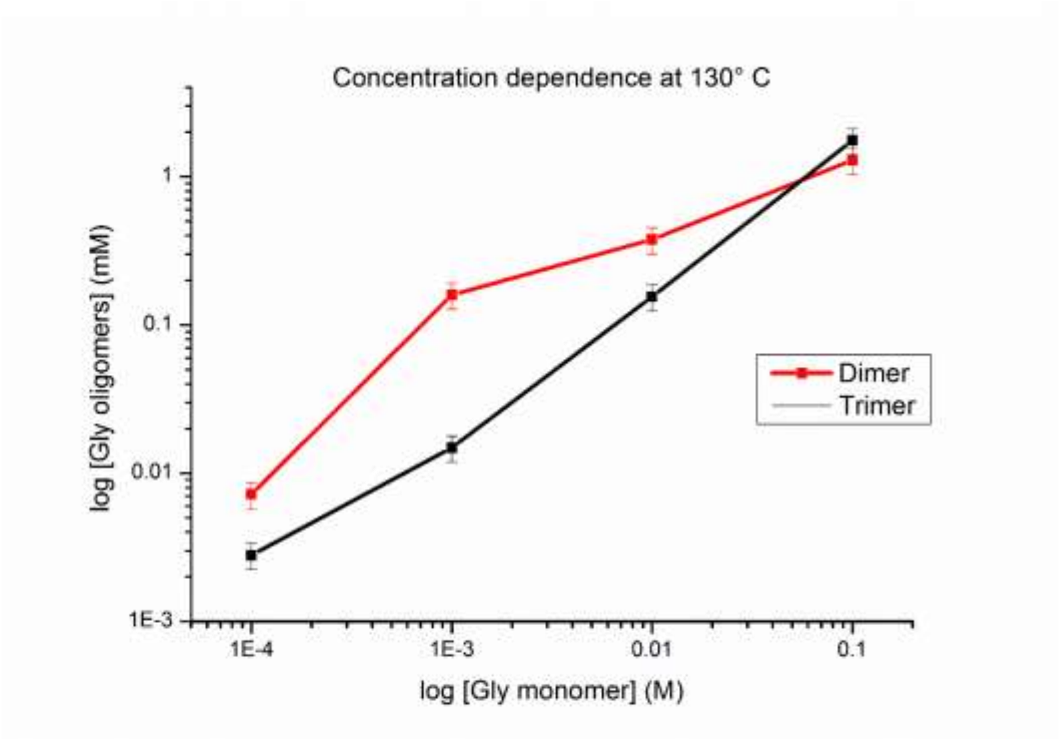

Supplementary Figure 15: Log-log plot of the concentration of glycine oligomers formed against glycine monomer input concentration.

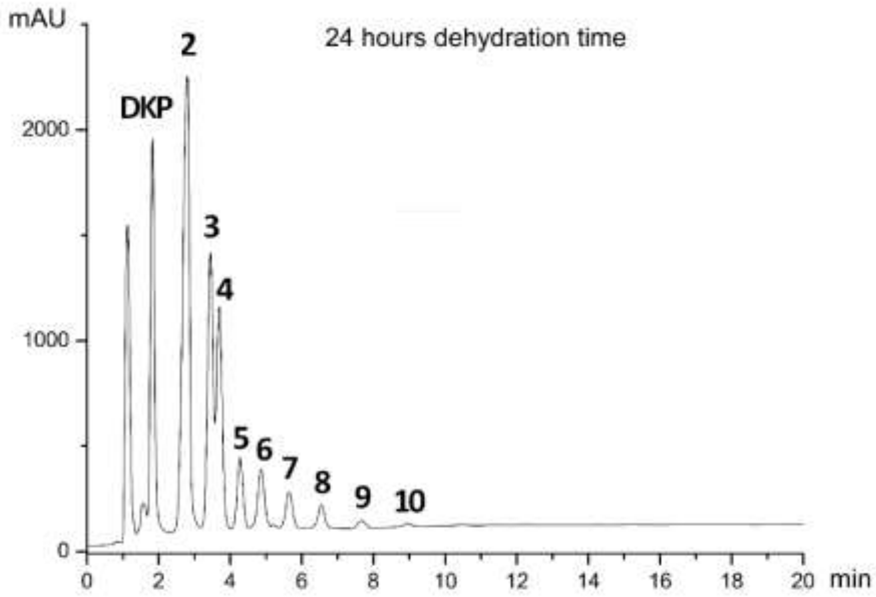

Supplementary Figure 16: IP-HPLC chromatogram of DKP oligomerisation products (24h, 130ºC).

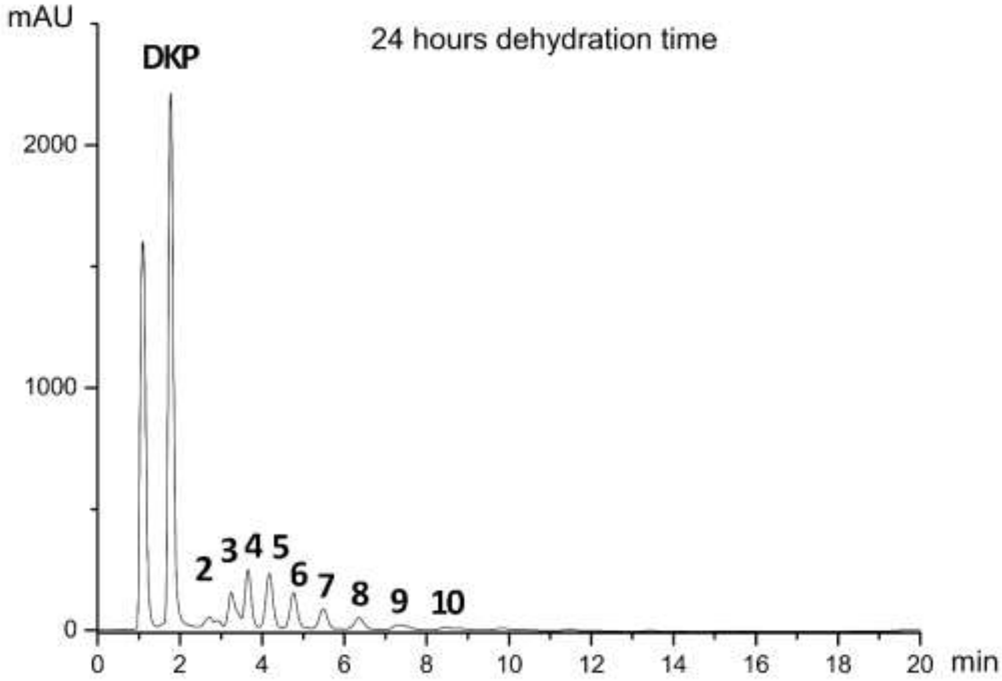

Supplementary Figure 17: IP-HPLC chromatogram of glycinamide oligomerisation products (24h, 130ºC).

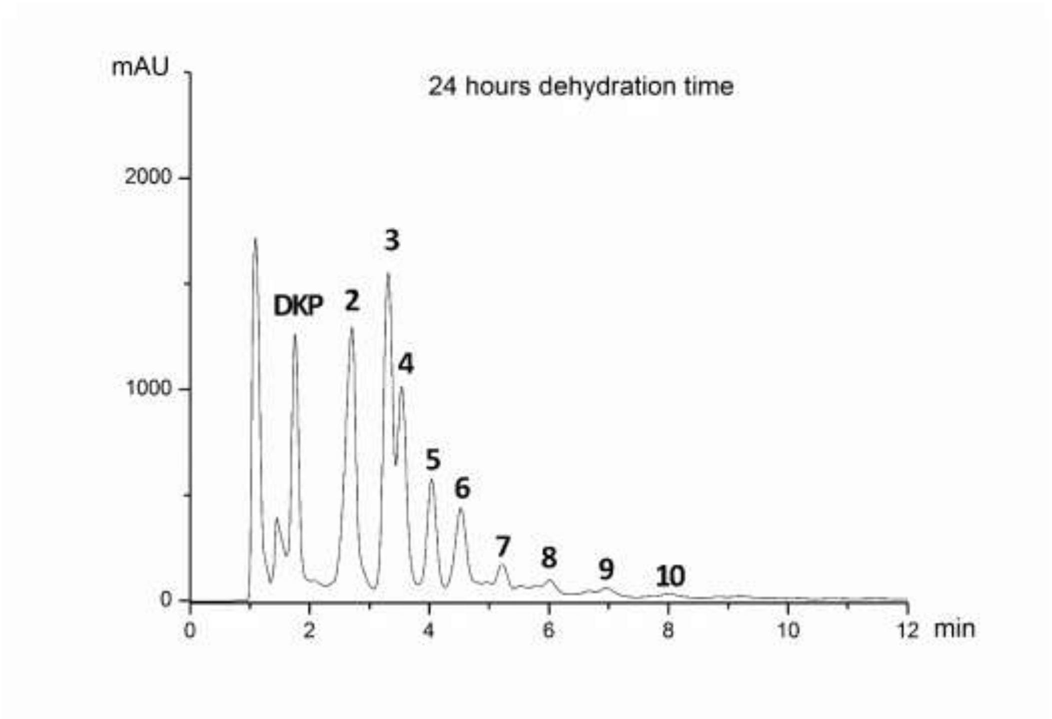

Supplementary Figure 18: IP-HPLC chromatogram of glycine oligomerisation products (24h, 130ºC) where a Teflon reaction vessel was used.

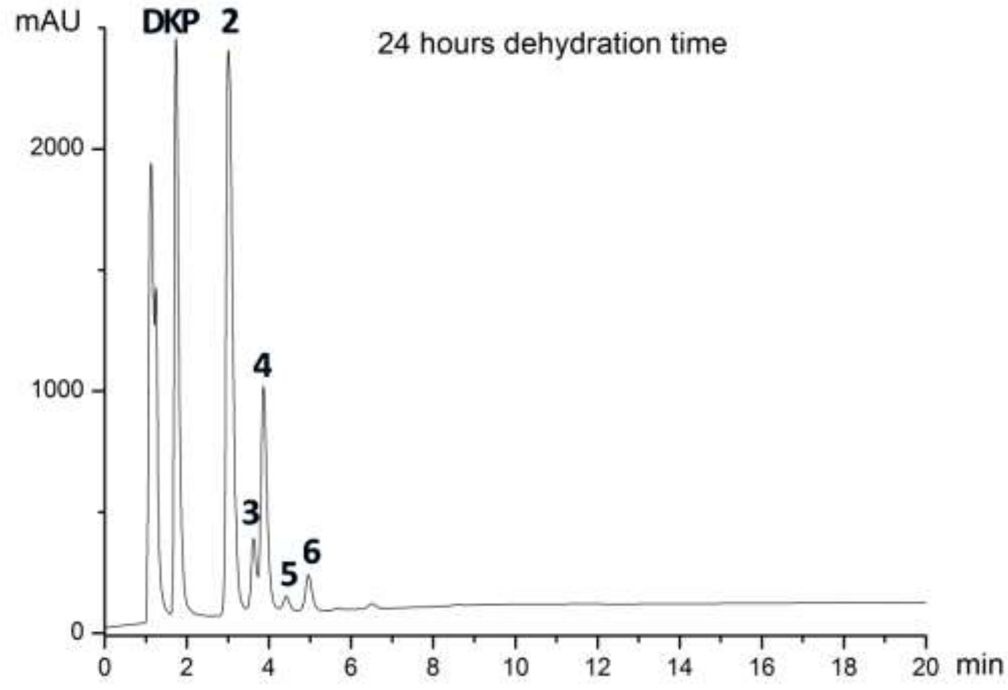

Supplementary Figure 19: IP-HPLC chromatogram of glycylglycine oligomerisation products (24h, 110ºC).

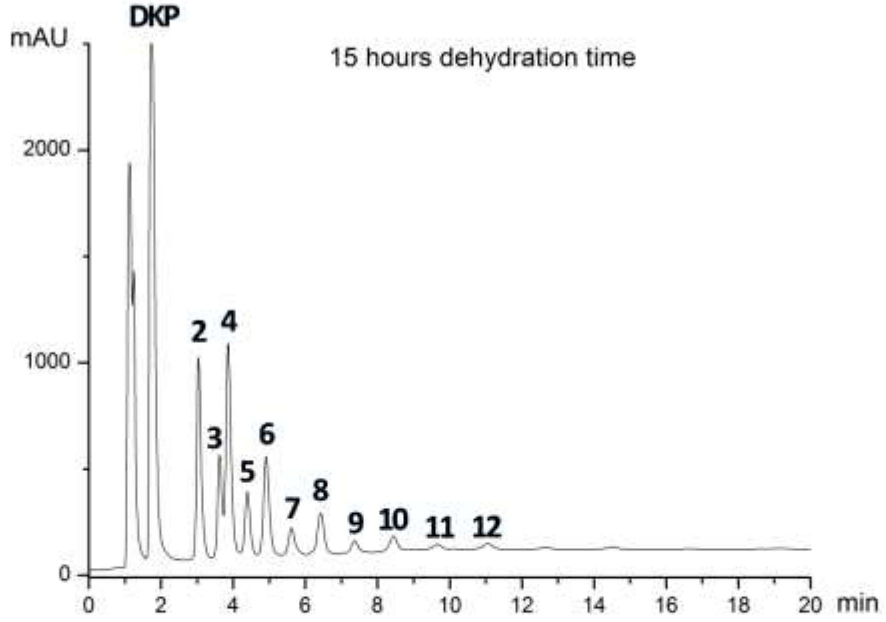

Supplementary Figure 20: IP-HPLC chromatogram of glycylglycine oligomerisation products (15h, 130ºC).

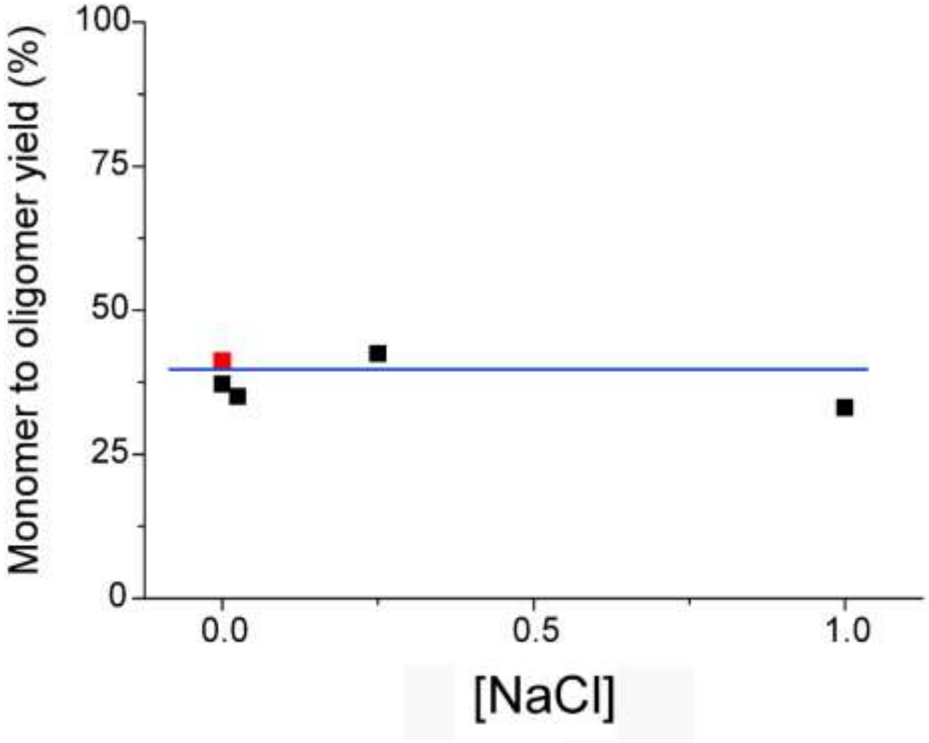

Supplementary Figure 21: Monomer to Oligomer yield (%) vs. added [NaCl] (M). The red point indicates the yield obtained in an experiment ran under the same conditions, but using LiOH to adjust the pH in the absence of NaCl.

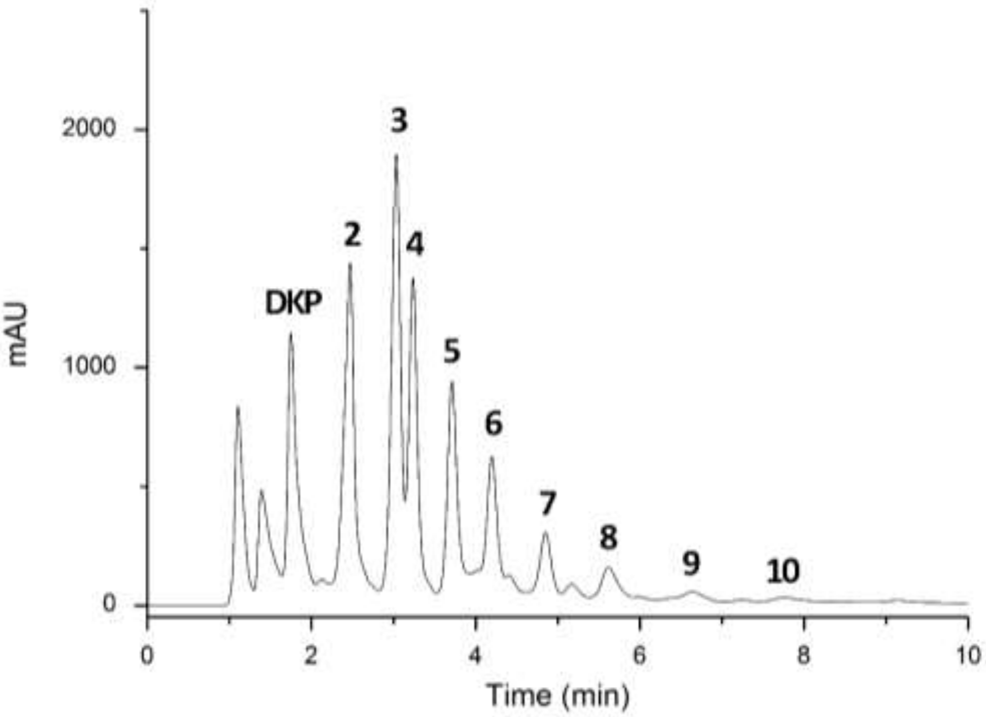

Supplementary Figure 22: IP-HPLC chromatogram of glycine oligomerisation products corresponding to the black point at an added [NaCl] = 0 in the previous plot. In this sample, glycine (350µl from a 1M Gly solution, so 3.5•10^{-4} moles) was diluted in a total volume of 4ml. The pH was adjusted to 9.75 by adding 100 µl of a 1M NaOH solution. No NaCl was added.

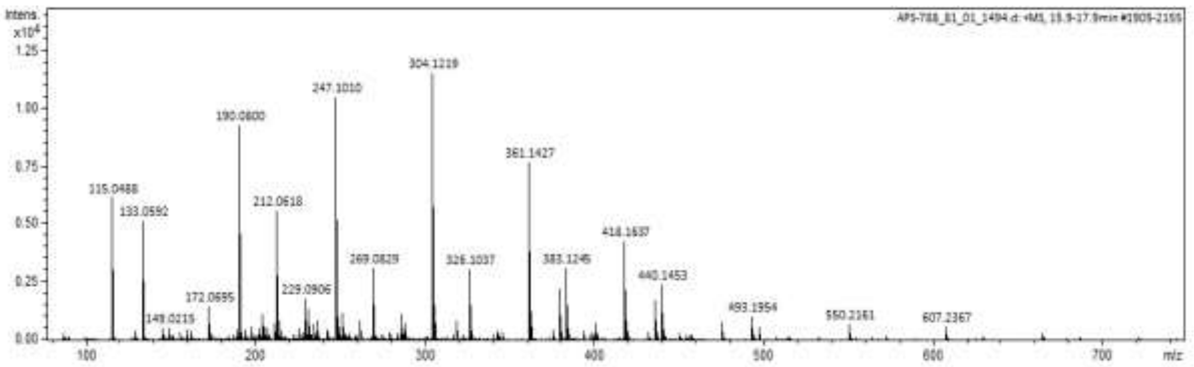

Supplementary Figure 23: MS-spectra corresponding to the glycine oligomerisation products from the reaction described in the figure above on elution from a Phenomenex Polysep 1000 SEC column (50 mM ammonium acetate w/ 10% v/v acetonitrile). Glycine oligomeric peaks up to 10-mer can be identified as H^+ adducts (along with a series of $NH4^+$ adducts).

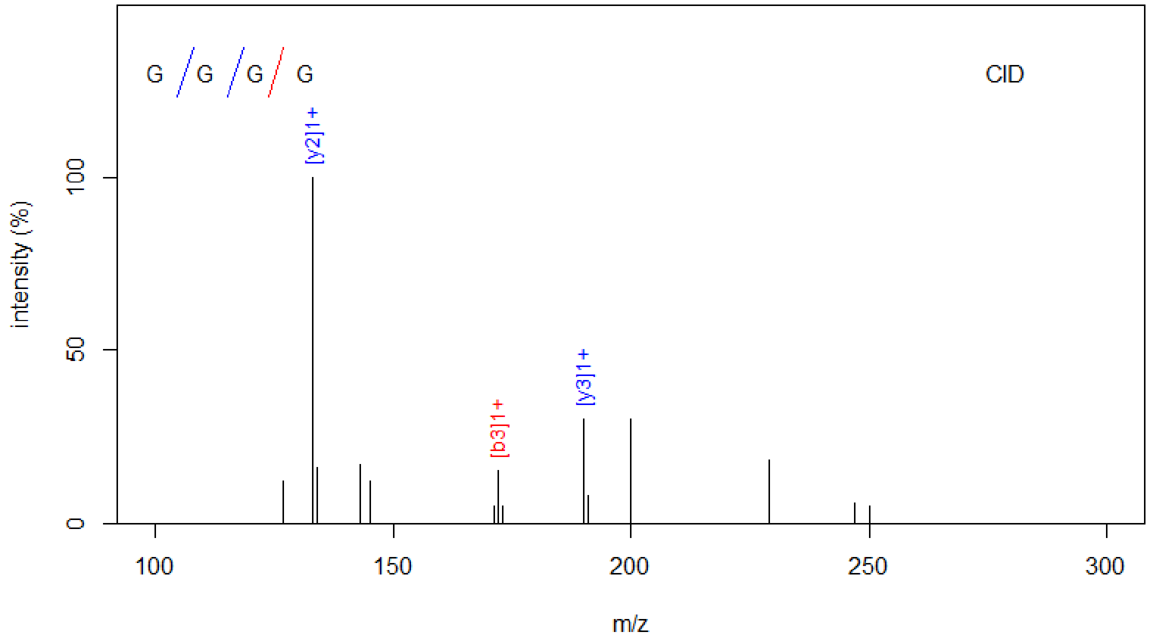

Supplementary Figure 24: MS/MS of fragmentation products of ion assigned as Gly_4 oligomer (m/z = 247.104); experimental data matches fragmentation products calculated for GGGG sequence.

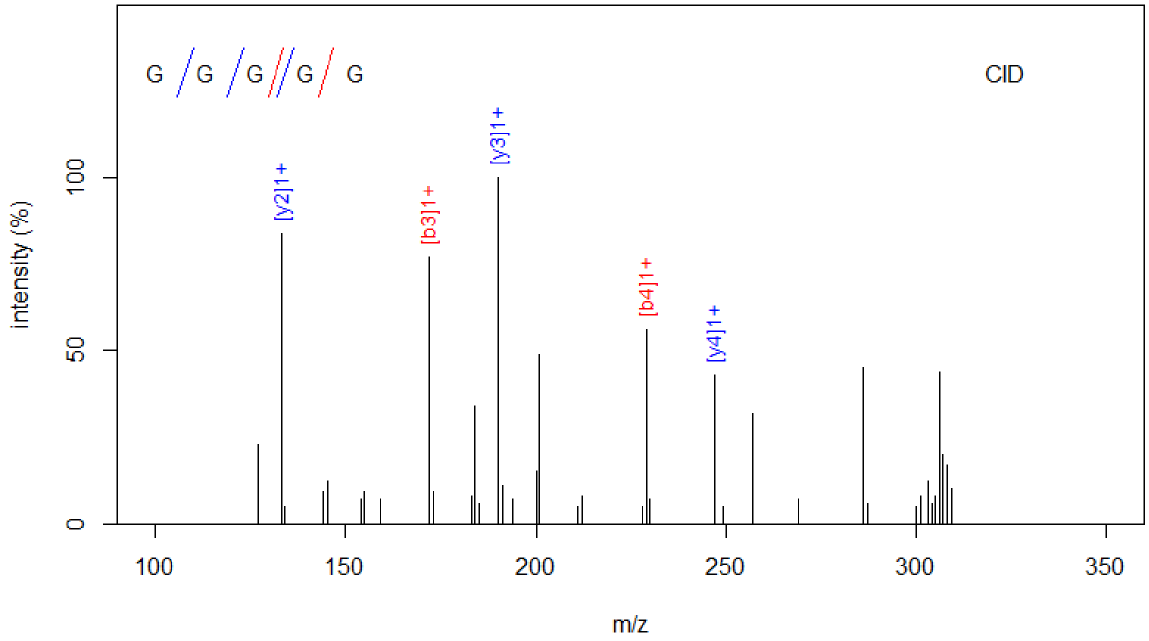

Supplementary Figure 25: MS/MS of fragmentation products of ion assigned as Gly_5 oligomer (m/z = 304.125); experimental data matches fragmentation products calculated for GGGGG sequence.

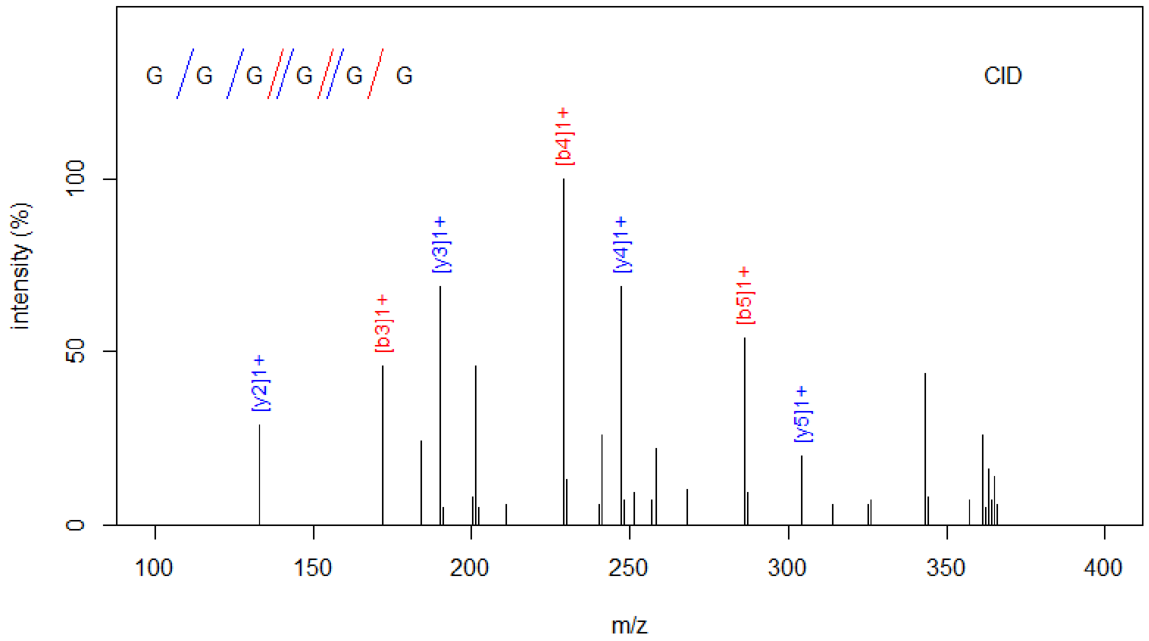

Supplementary Figure 26: MS/MS of fragmentation products of ion assigned as Gly_6 oligomer (m/z = 361.147); experimental data matches fragmentation products calculated for GGGGGG sequence.

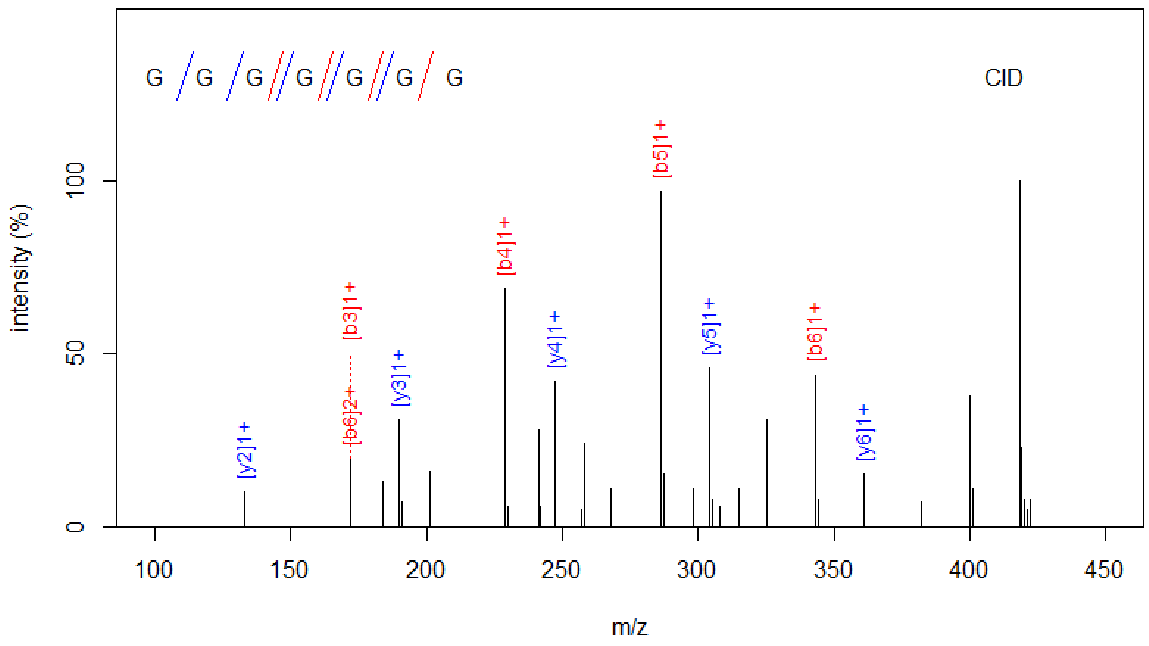

Supplementary Figure 27: MS/MS of fragmentation products of ion assigned as Gly_7 oligomer (m/z = 418.168); experimental data matches fragmentation products calculated for GGGGGGG sequence.

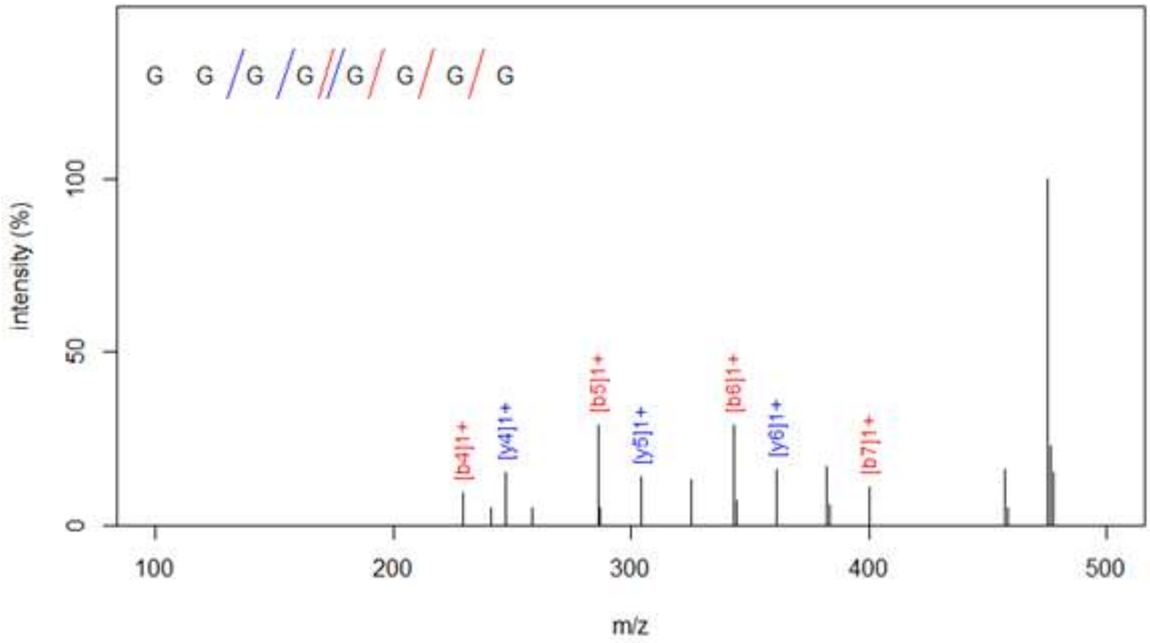

Supplementary Figure 28: MS/MS of fragmentation products of ion assigned as Gly_8 oligomer (m/z = 475.190); experimental data matches fragmentation products calculated for GGGGGGGG sequence.

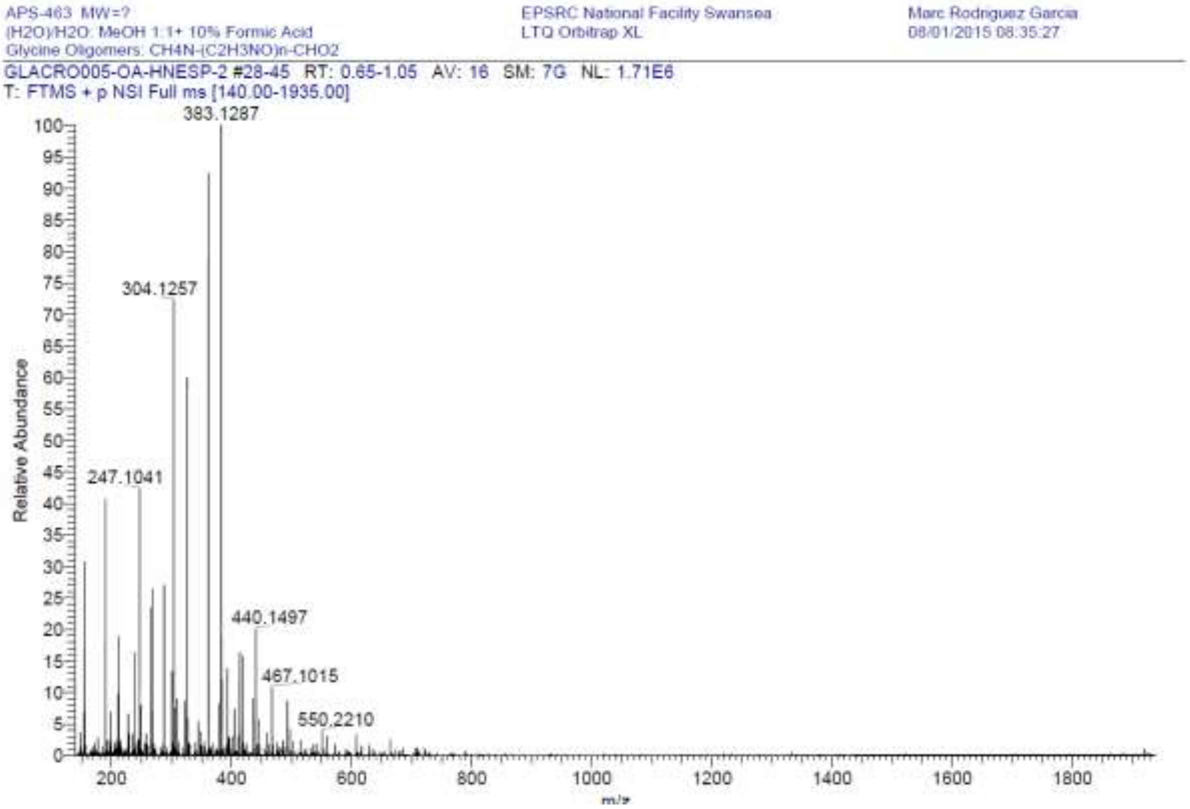

Supplementary Figure 29: Direct Infusion MS analysis of a typical APS reaction.

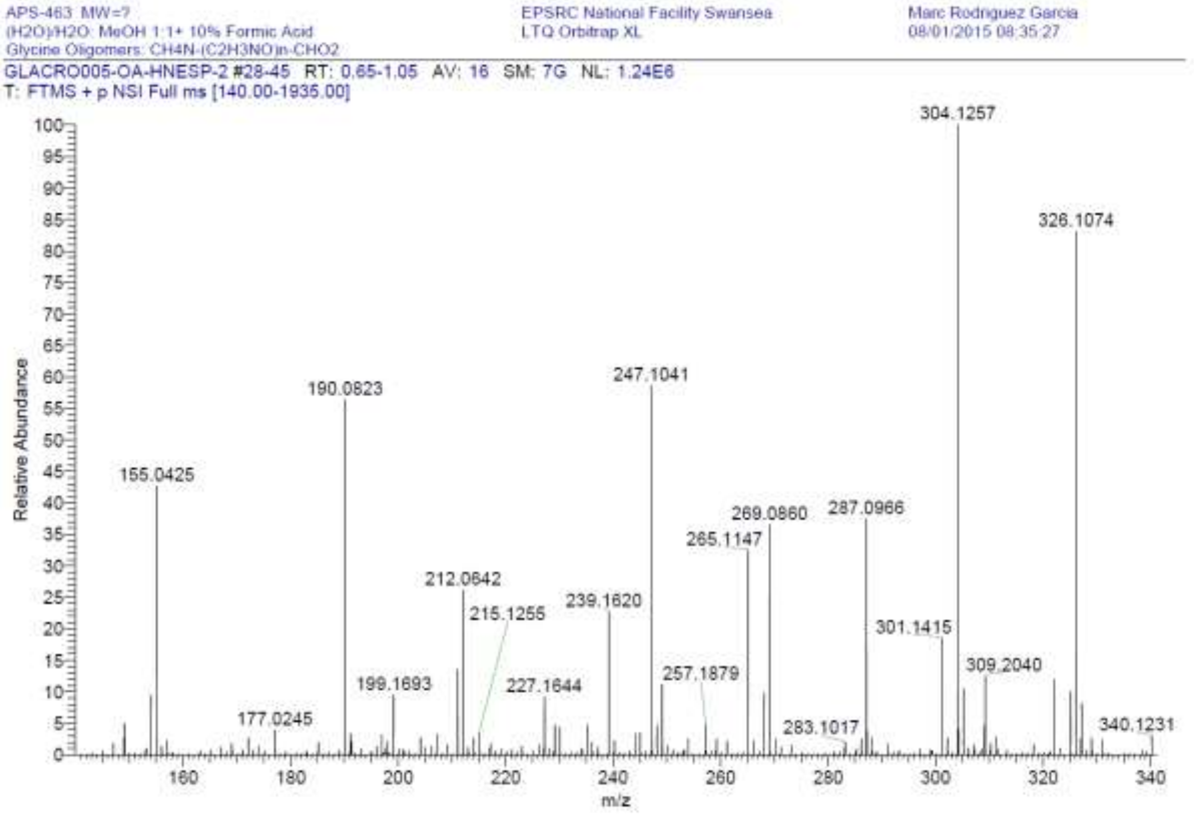

Supplementary Figure 30: Direct Infusion MS analysis of a typical APS reaction.

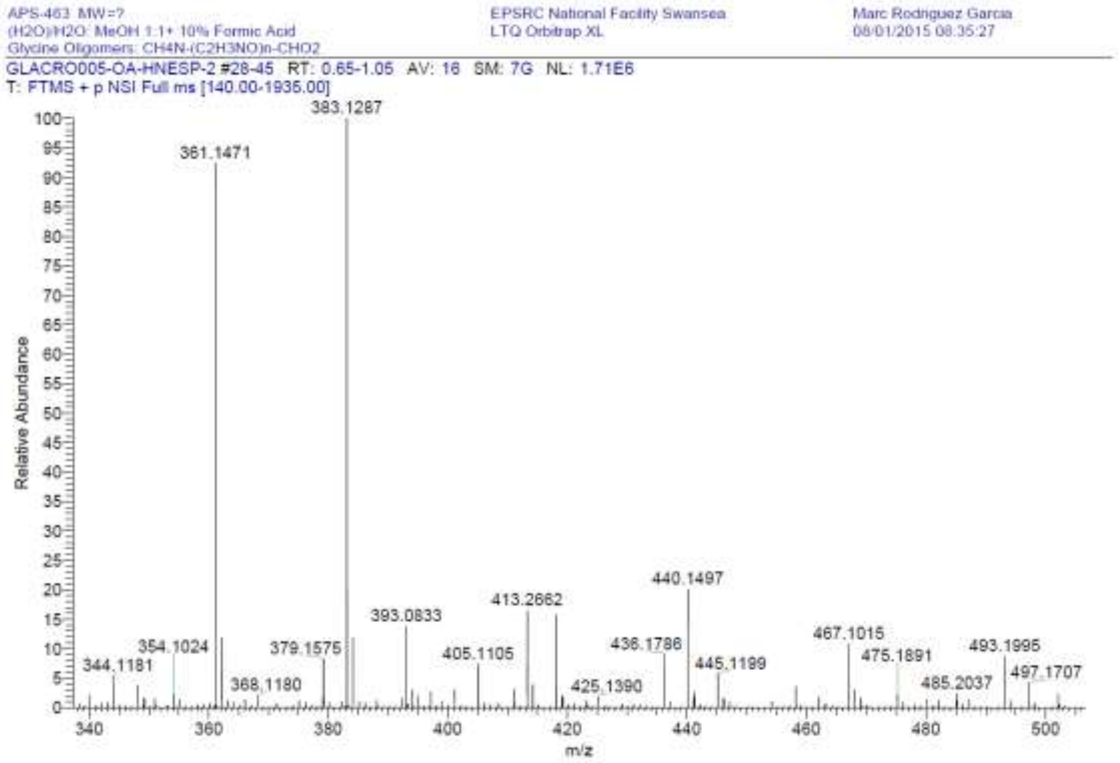

Supplementary Figure 31: Direct Infusion MS analysis of a typical APS reaction.

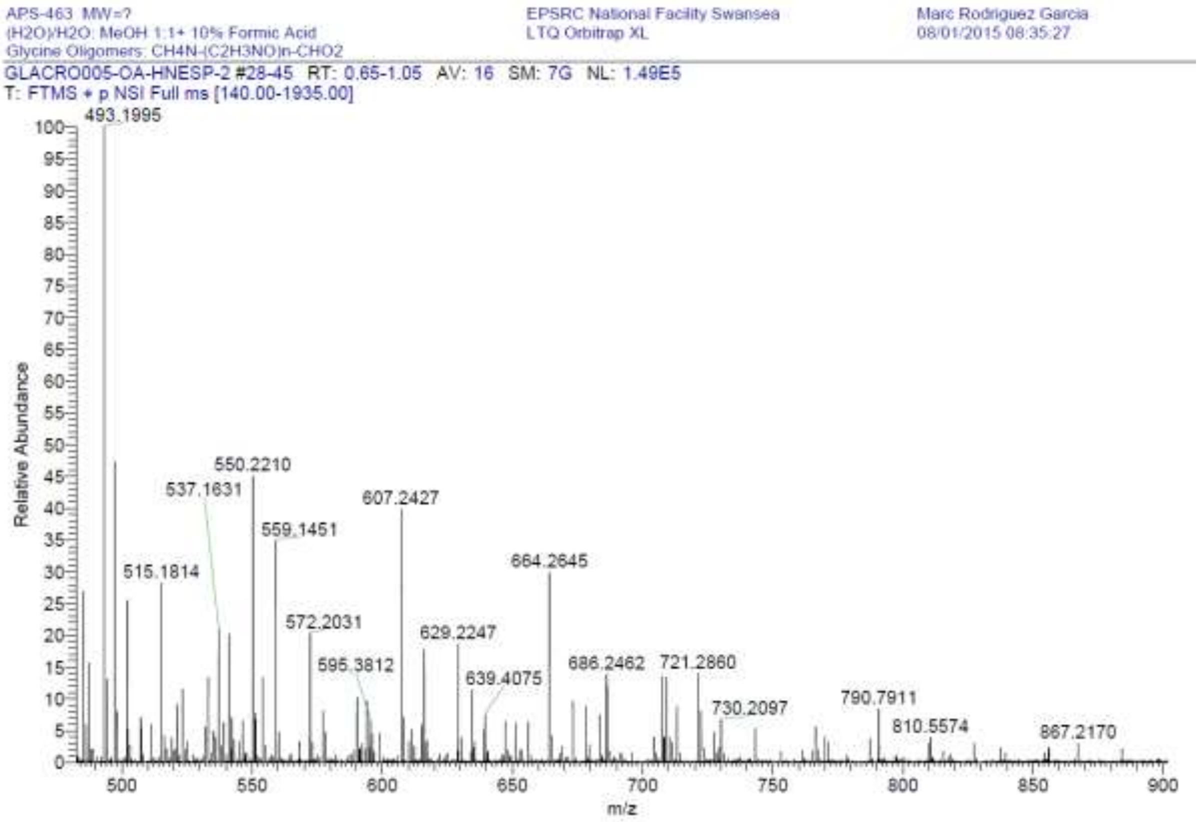

Supplementary Figure 32: Direct Infusion MS analysis of a typical APS reaction.

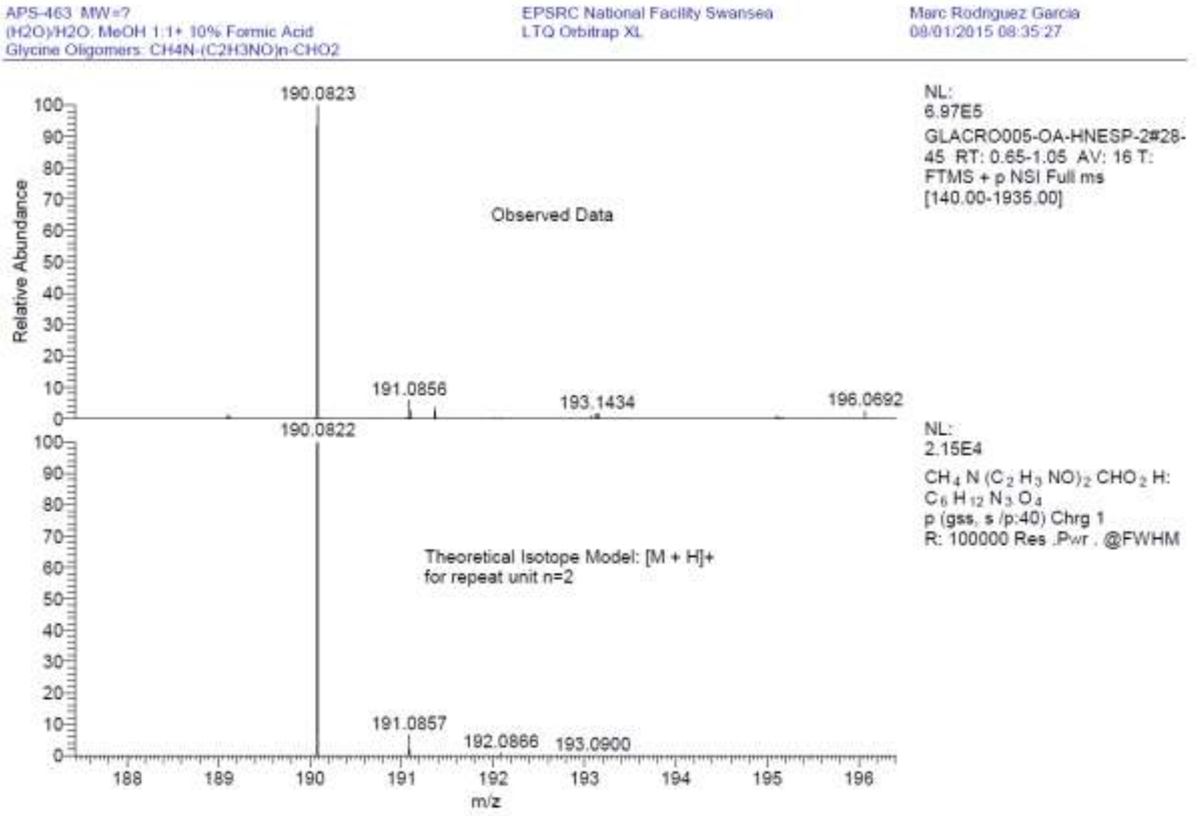

Supplementary Figure 33: Direct Infusion MS analysis of a typical APS reaction.

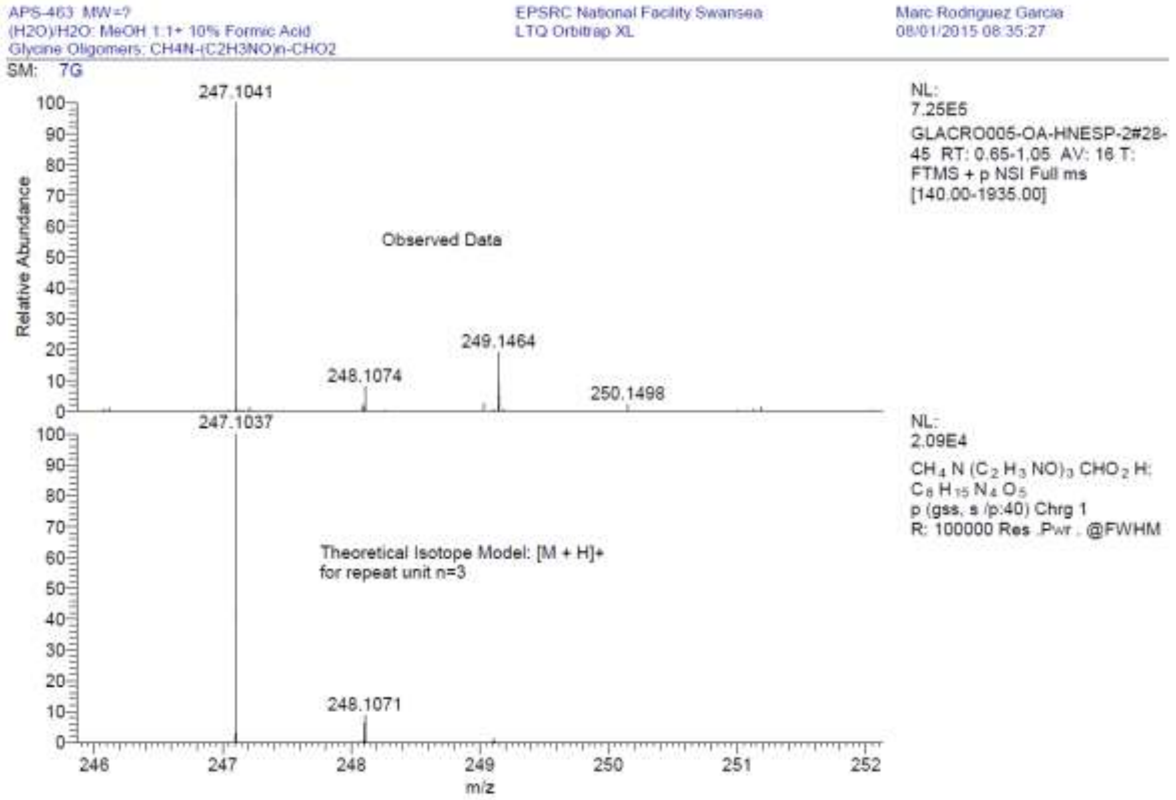

Supplementary Figure 34: Direct Infusion MS analysis of a typical APS reaction.

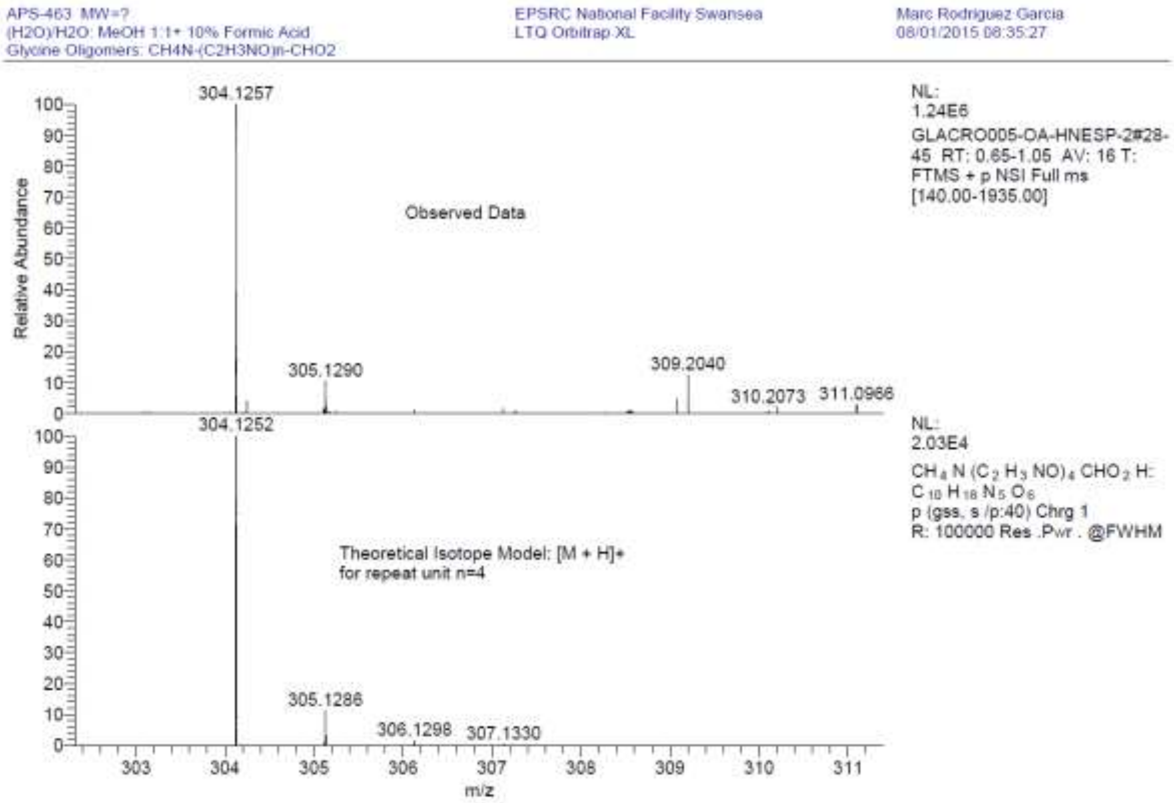

Supplementary Figure 35: Direct Infusion MS analysis of a typical APS reaction.

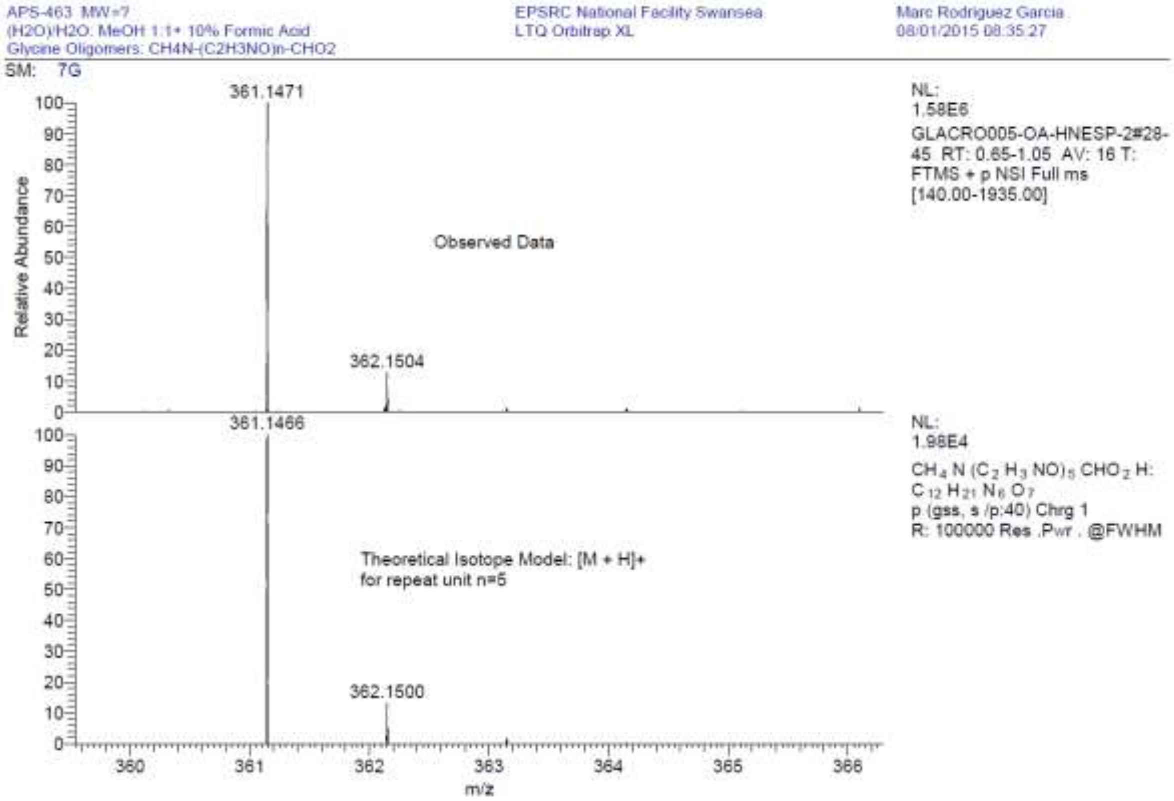

Supplementary Figure 36: Direct Infusion MS analysis of a typical APS reaction.

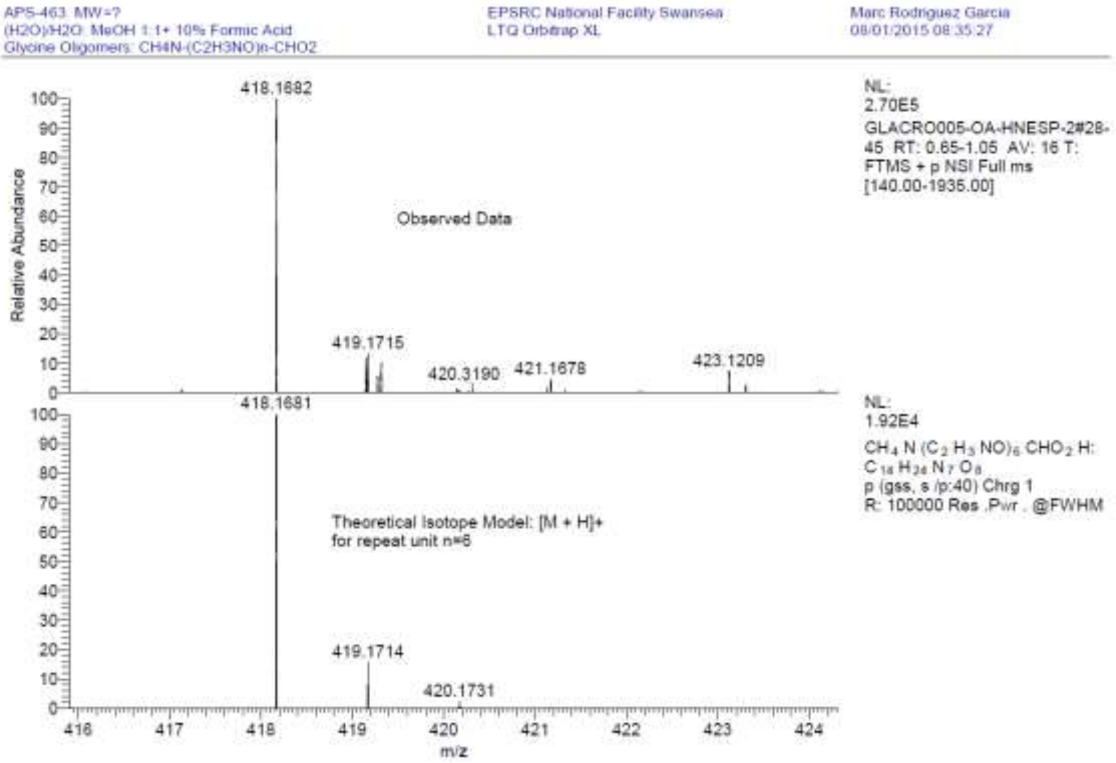

Supplementary Figure 37: Direct Infusion MS analysis of a typical APS reaction.

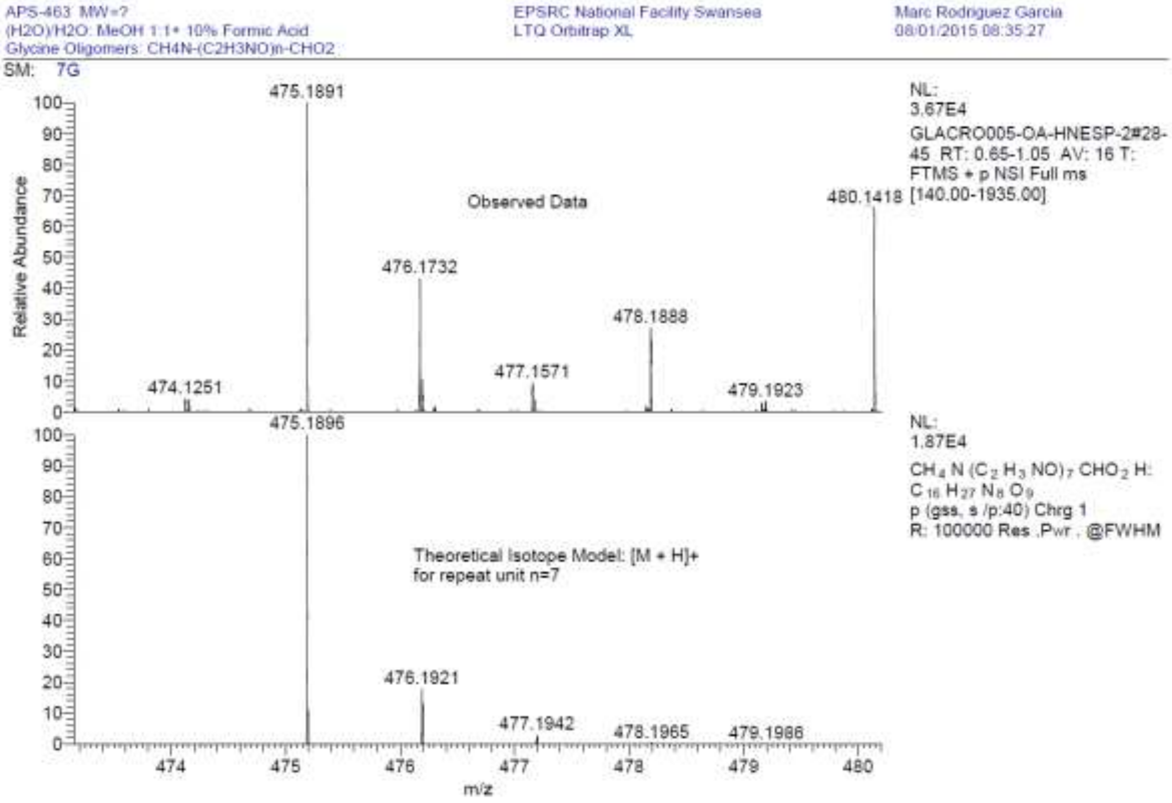

Supplementary Figure 38: Direct Infusion MS analysis of a typical APS reaction.

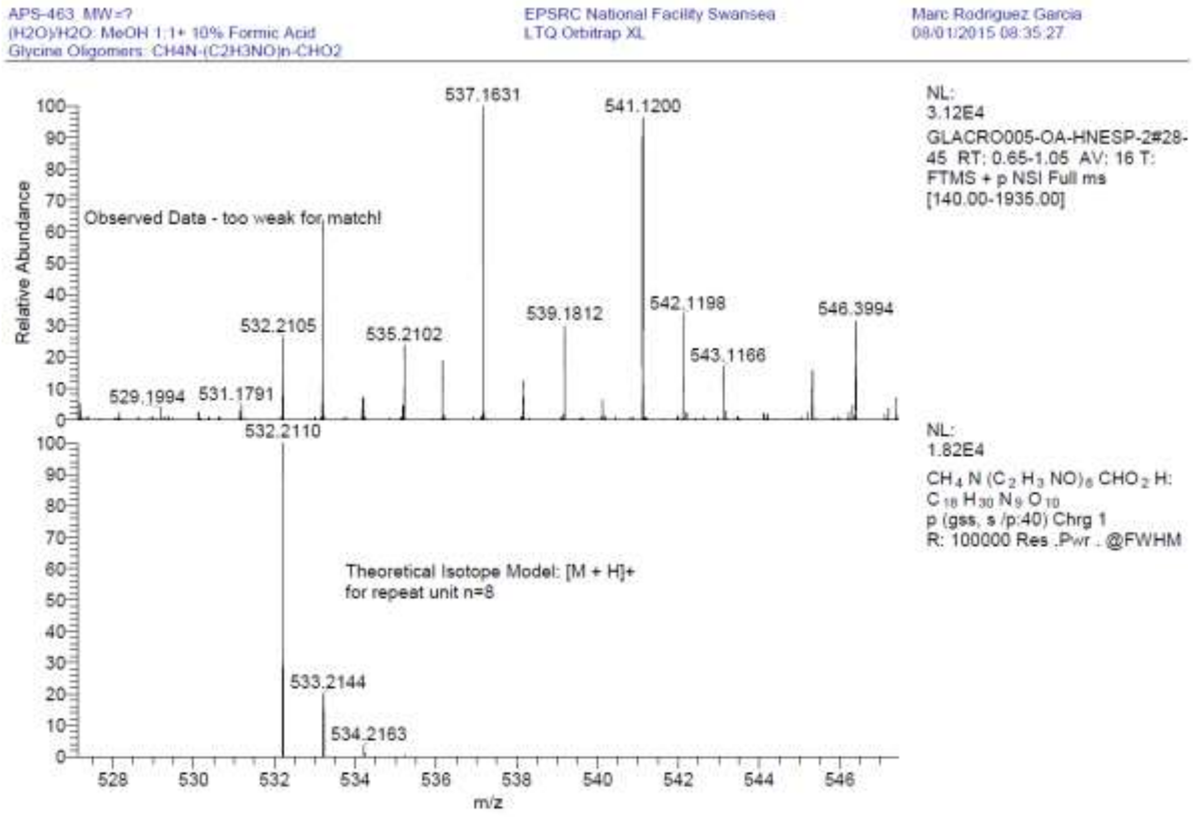

Supplementary Figure 39: Direct Infusion MS analysis of a typical APS reaction.

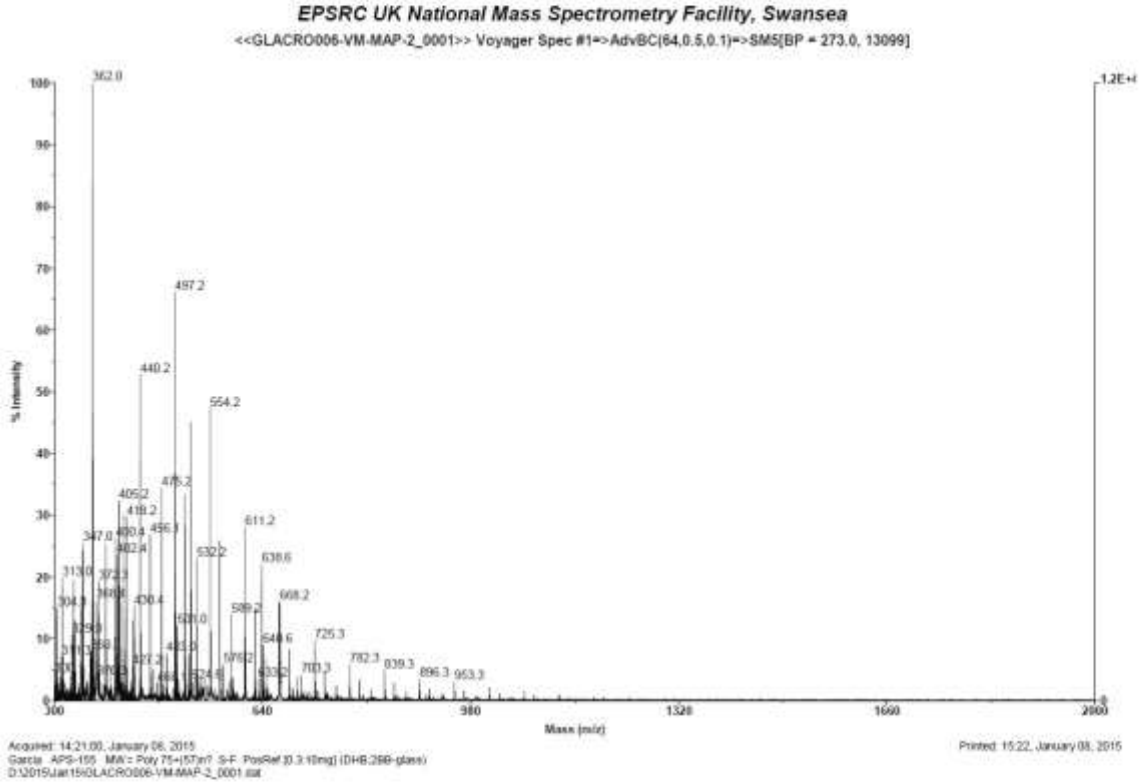

Supplementary Figure 40: MALDI-TOF analysis of a solid fraction

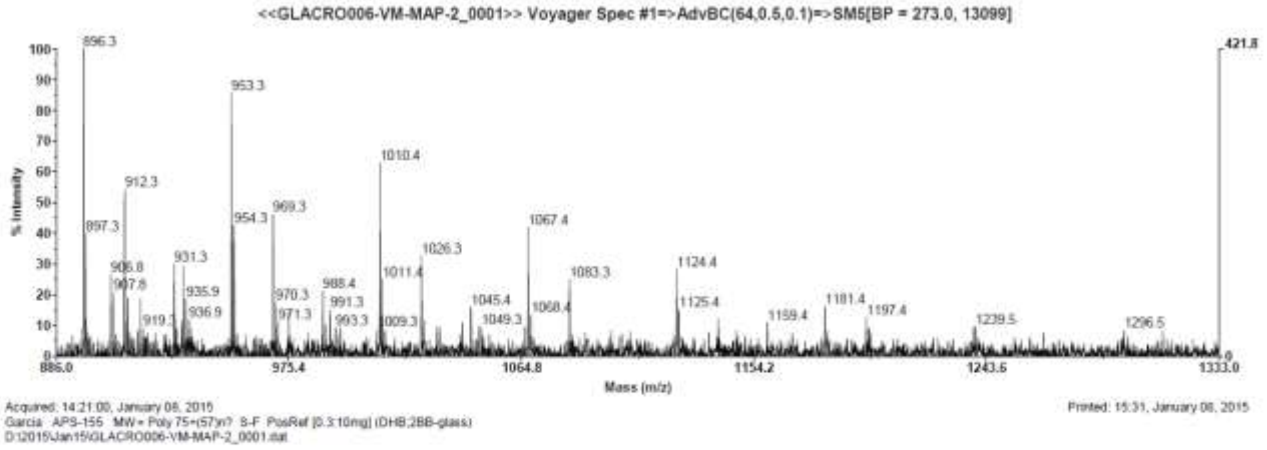

Supplementary Figure 41: MALDI-TOF analysis of a solid fraction (zoom in high mass region). Note peak at 1181.4, corresponding to $[C_{40}H_{62}N_{20}O_{21}Na]^+$, the composition of the sodium ESI adduct of $(Gly)_{20}$.

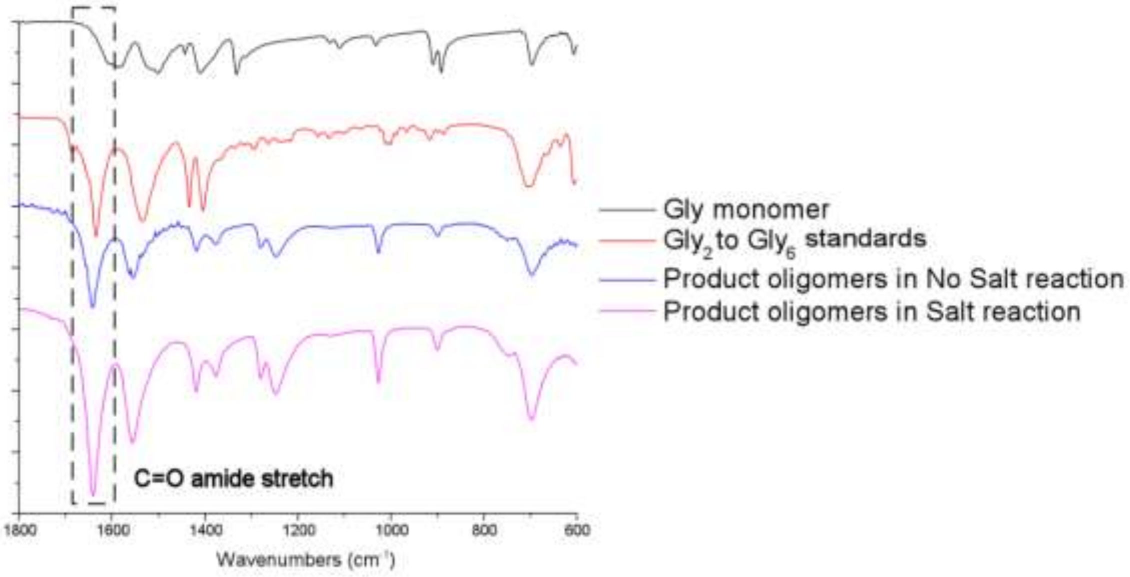

Supplementary Figure 42: C=O amide stretching IR band of glycine monomer, gly_2-gly_6 standard oligomers, and two product oligomer samples run with different NaCl concentrations. As can be seen the IR of our samples indeed matches the oligomers which confirms our HPLC results.

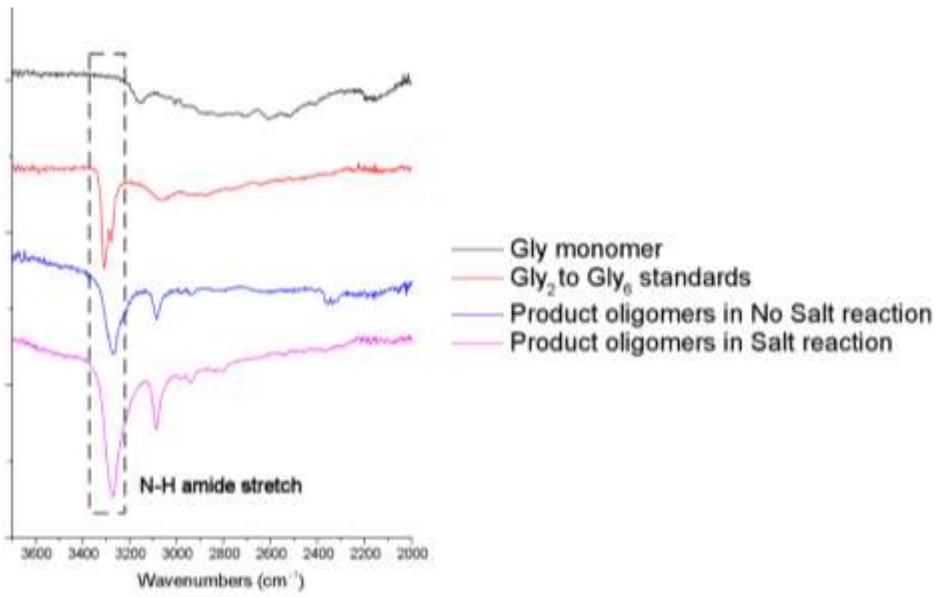

Supplementary Figure 43: N-H amide stretching IR band of glycine monomer, gly$_2$-gly$_6$ standard oligomers, and two product oligomer samples ran with different NaCl concentrations. As can be seen the IR of our samples indeed matches the oligomers which confirms our HPLC results.

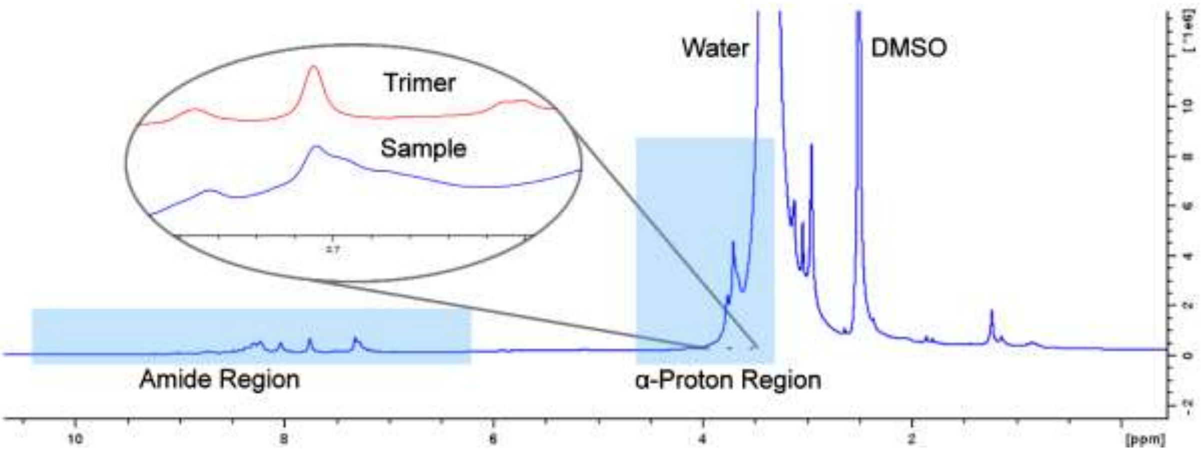

Supplementary Figure 44: ^{1}H-NMR spectrum of APS products in DMSO highlighting amide and α-proton regions.

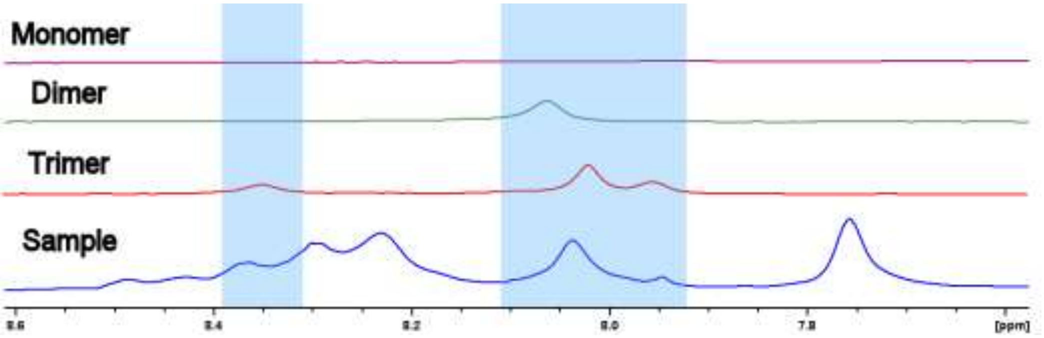

Supplementary Figure 45: 1H-NMR comparison of Gly, Gly_2, Gly_3 and the APS sample in the amide region.

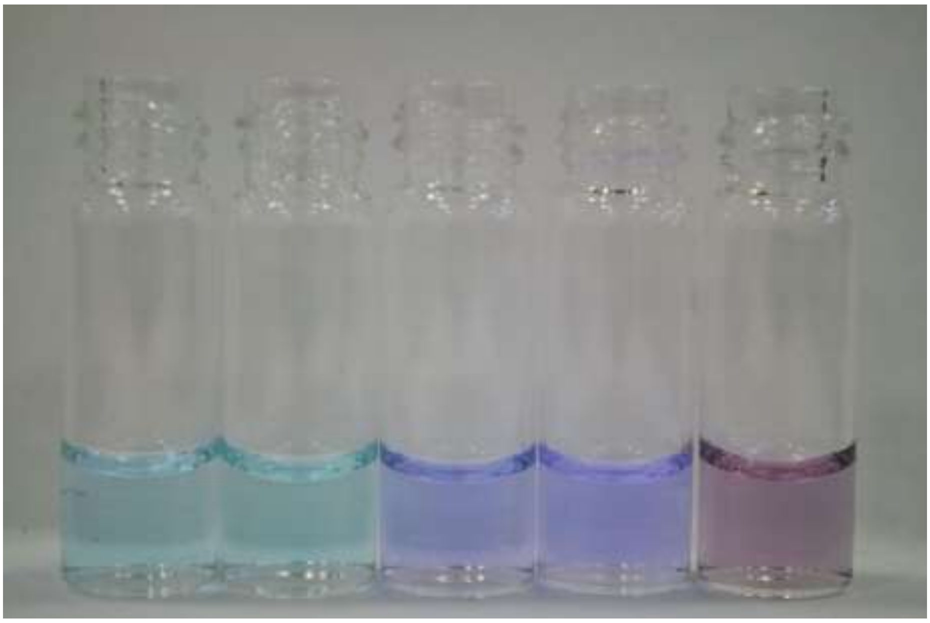

Supplementary Figure 46: Biuret tests for Gly,Gly_2, Gly_3 & material from the APS after 1 and 3 dehydration cycles.

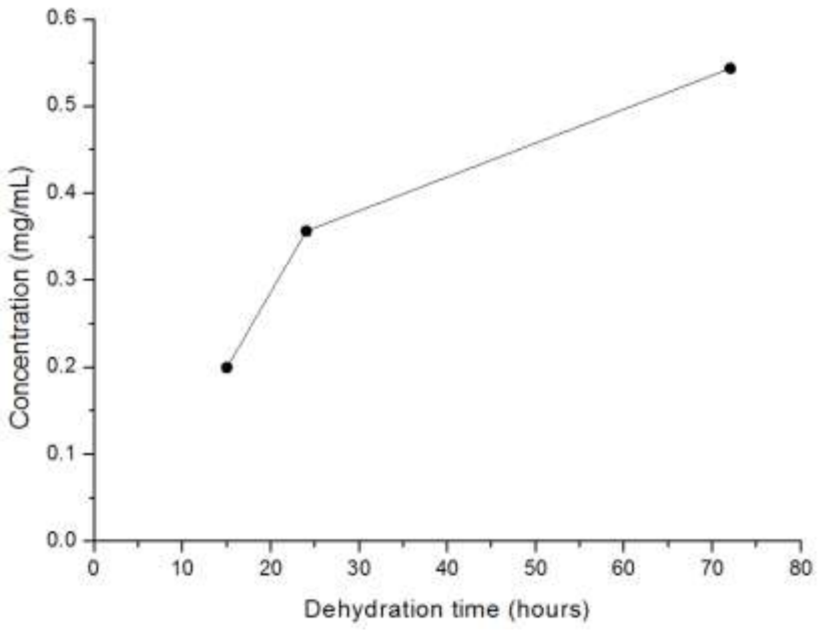

Supplementary Figure 47: Concentration of peptidic material in APS samples after different dehydration times based on Biuret assay.

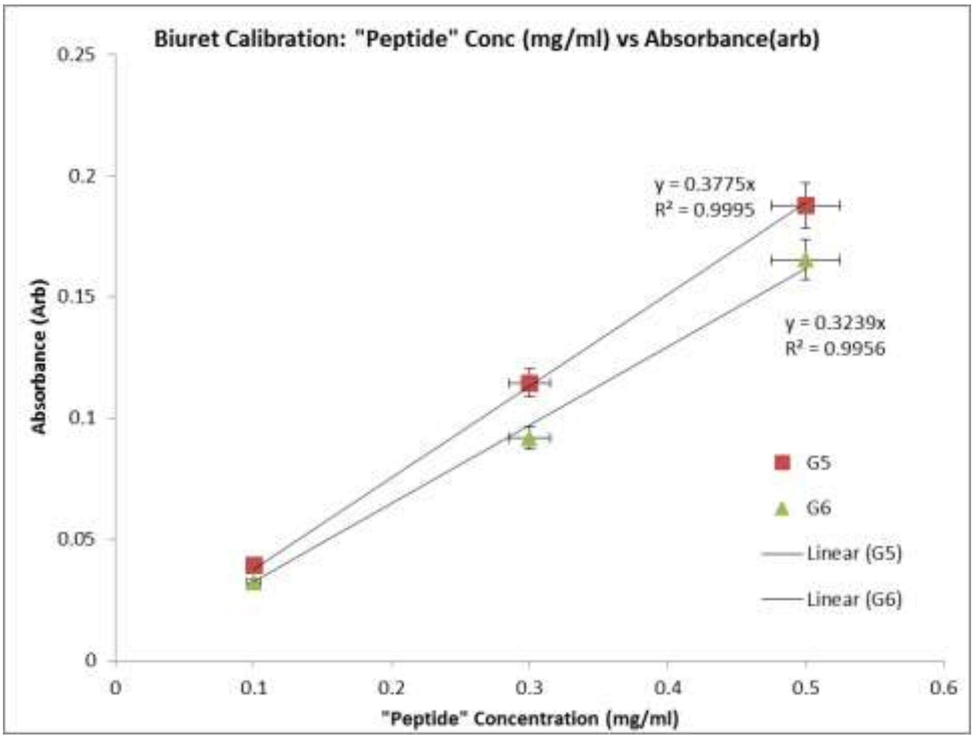

Supplementary Figure 48: Biuret calibration using known concentrations of both Gly_5 and Gly_6.

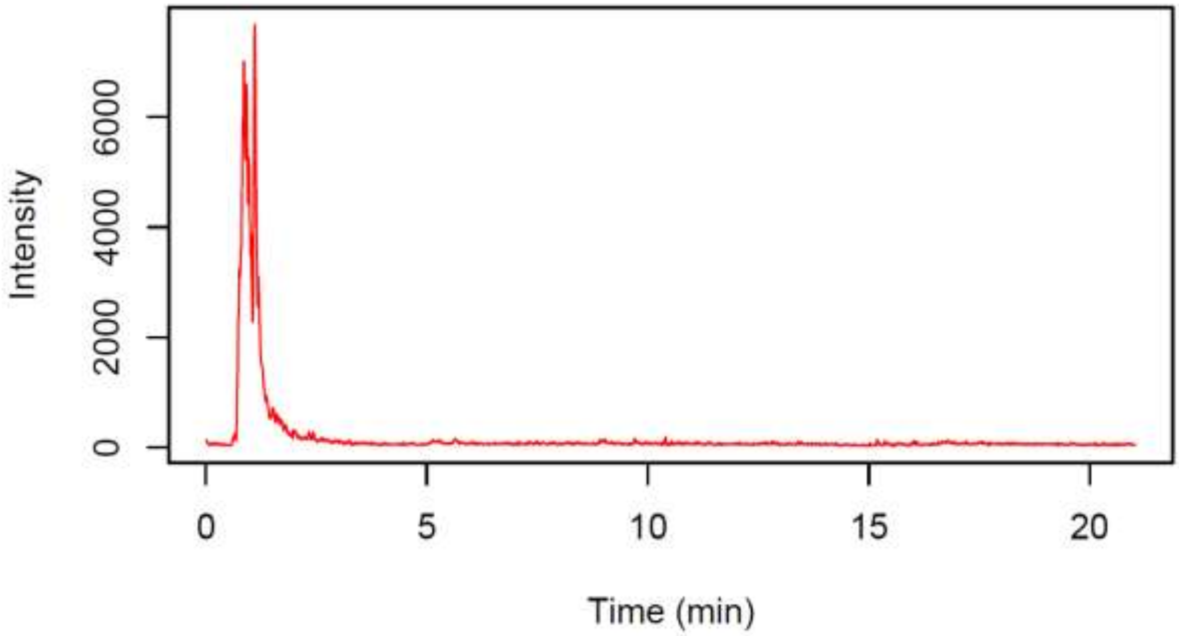

Supplementary Figure 49: "Virtual" BPC traces illustrating co-condensation of G and A monomers. VBPC is constructed from the EICs observed for a list of putative oligomers incorporating both of the amino acids monomers.

Experimental Product ion m/z	Possible parent sequence(s) at m/z=363.115	Possible production sequence(s)	Fragment type	MSMS m/z	Error
90.054	GGAA	A	[y1]1+	90.055	-0.001
90.054	GAGA	A	[y1]1+	90.055	-0.001
90.054	AGGA	A	[y1]1+	90.055	-0.001
115.05	GGAA	GG	[b2]1+	115.05	0
129.0656	GAGA	GA	[b2]1+	129.066	0
129.0656	GAAG	GA	[b2]1+	129.066	0
129.0656	AGGA	AG	[b2]1+	129.066	0
129.0656	AGAG	AG	[b2]1+	129.066	0
133.0613	AAGG	GG	[y2]1+	133.061	0
147.0752	GAGA	GA	[y2]1+	147.076	-0.001
147.0752	GAAG	AG	[y2]1+	147.076	-0.001
147.0752	AGGA	GA	[y2]1+	147.076	-0.001
147.0752	AGAG	AG	[y2]1+	147.076	-0.001
186.0859	GGAA	GGA	[b3]1+	186.087	-0.001
186.0859	GAGA	GAG	[b3]1+	186.087	-0.001
186.0859	AGGA	AGG	[b3]1+	186.087	-0.001
204.0965	AGGA	GGA	[y3]1+	204.098	-0.002
204.0965	AGAG	GAG	[y3]1+	204.098	-0.002
204.0965	AAGG	AGG	[y3]1+	204.098	-0.002

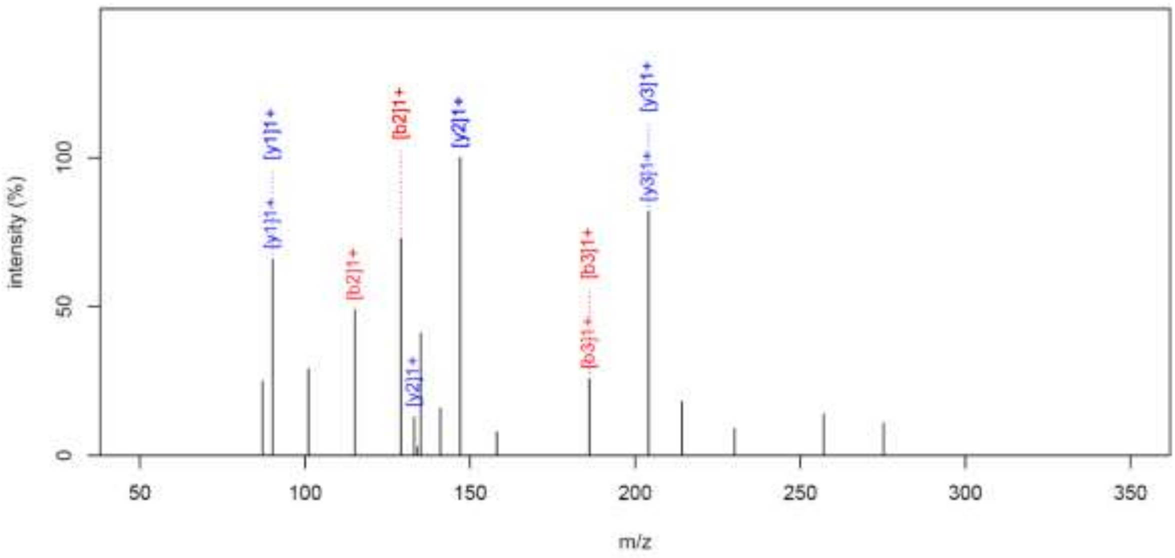

Supplementary Figure 50: Example MS/MS analyses of products of G and A monomer co-oligomerisation experiments. Spectrum (above) shows all the product ions resulting from MS/MS analysis of m/z = 275.135 (corresponding to a tetramer incorporating 2 x G and 2x A). A series of fragments are observed which can be assigned as those expected (below) from a number of different possible sequences which might be expected from tetramers incorporating 2 x G and 2 x A (ie. more than one sequence of the same composition is present, all incorporating G and A).

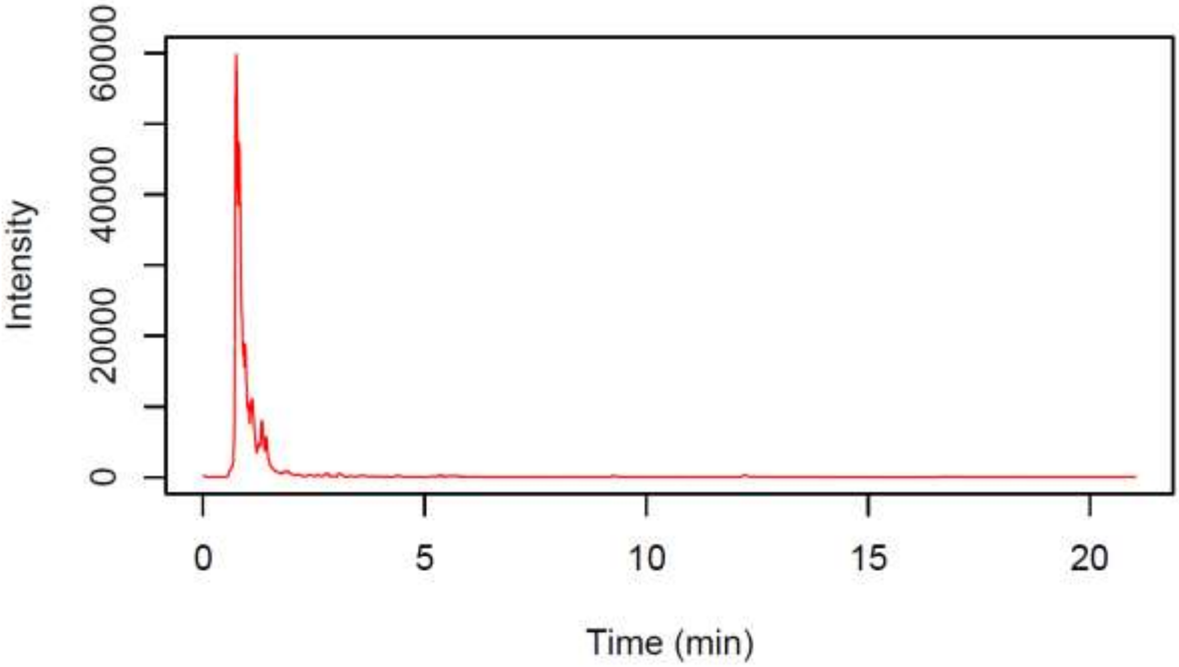

Supplementary Figure 51: "Virtual" BPC traces illustrating co-condensation of G and D monomers. VBPC is constructed from the EICs observed for a list of putative oligomers incorporating both of the amino acids monomers.

Experimental Product ion m/z	Possible parent sequence(s) at m/z=363.115	Possible production sequence(s)	Fragment type	MSMS m/z	Error
76.0327	GDDG	G	[y1]1+	76.039	-0.006
76.0327	DGDG	G	[y1]1+	76.039	-0.006
76.0327	DDGG	G	[y1]1+	76.039	-0.006
115.051	GGDD	GG	[b2]1+	115.05	0.001
173.0516	GDGD	GD	[b2]1+	173.056	-0.004
173.0516	GDDG	GD	[b2]1+	173.056	-0.004
173.0516	DGGD	DG	[b2]1+	173.056	-0.004
173.0516	DGDG	DG	[b2]1+	173.056	-0.004
191.0673	GDGD	GD	[y2]1+	191.066	0.001
191.0673	GDDG	DG	[y2]1+	191.066	0.001
191.0673	DGGD	GD	[y2]1+	191.066	0.001
191.0673	DGDG	DG	[y2]1+	191.066	0.001
230.0741	GGDD	GGD	[b3]1+	230.077	-0.003
230.0741	GDGD	GDG	[b3]1+	230.077	-0.003
230.0741	DGGD	DGG	[b3]1+	230.077	-0.003
231.0617	DDGG	DD	[b2]1+	231.061	0.001
248.0845	DGGD	GGD	[y3]1+	248.088	-0.004
248.0845	DGDG	GDG	[y3]1+	248.088	-0.004
248.0845	DDGG	DGG	[y3]1+	248.088	-0.004
249.0646	GGDD	DD	[y2]1+	249.072	-0.007
288.077	GDDG	GDD	[b3]1+	288.083	-0.006
288.077	DGDG	DGD	[b3]1+	288.083	-0.006
288.077	DDGG	DDG	[b3]1+	288.083	-0.006
306.091	GGDD	GDD	[y3]1+	306.093	-0.002
306.091	GDGD	DGD	[y3]1+	306.093	-0.002
306.091	GDDG	DDG	[y3]1+	306.093	-0.002

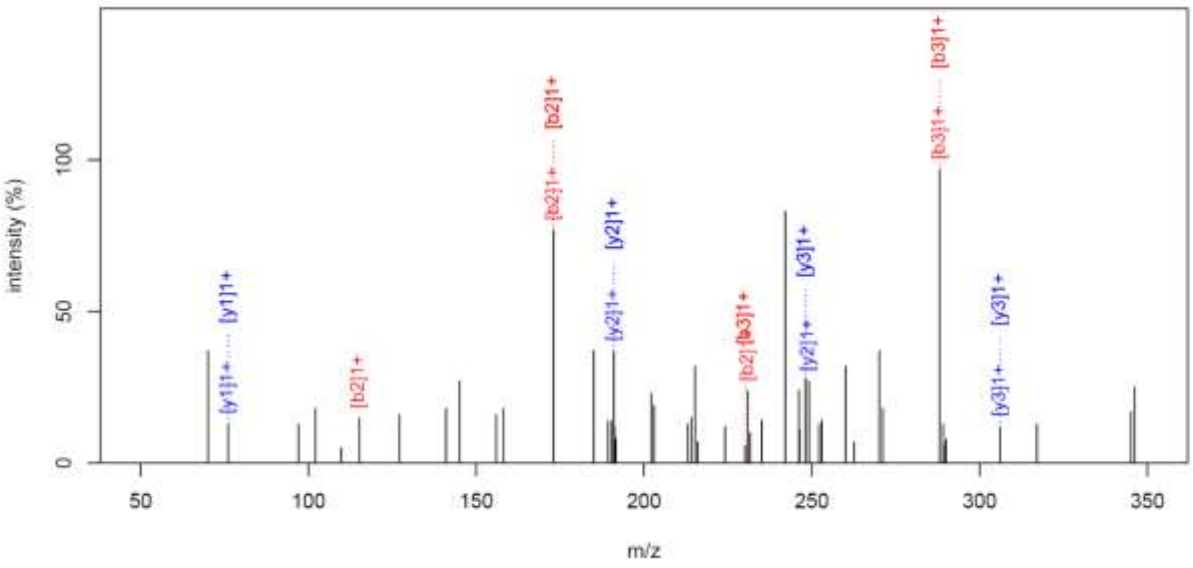

Supplementary Figure 52: Example MS/MS analyses of products of G and D monomer co-oligomerisation experiments. Spectrum (above) shows all the product ions resulting from MS/MS analysis of m/z = 363.115 (corresponding to a tetramer incorporating 2 x G and 2 x D). A series of fragments are observed which can be assigned as those expected (below) from a number of different possible sequences which might be expected from tetramers incorporating 2 x G and 2 x D (ie. more than one sequence of the same composition is present, all incorporating G and D).

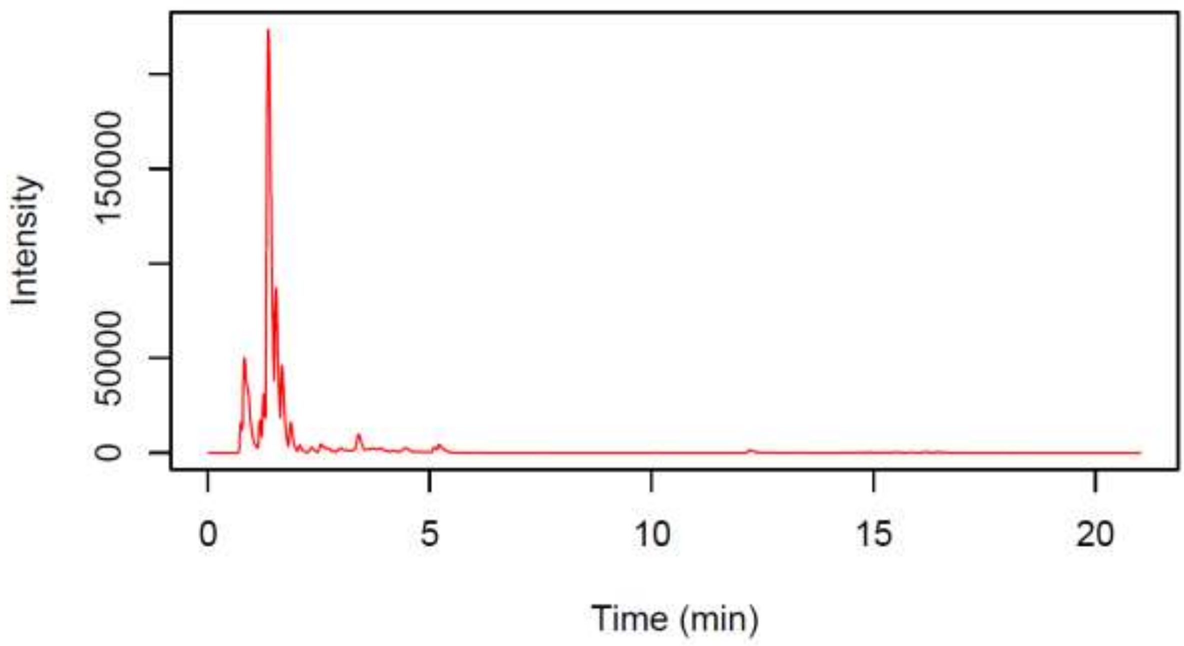

Supplementary Figure 53: "Virtual" BPC traces illustrating co-condensation of G and E monomers. VBPC is constructed from the EICs observed for a list of putative oligomers incorporating both of the amino acids monomers.

Experimental Product ion m/z	Possible parent sequence(s) at m/z=363.115	Possible production sequence(s)	Fragment type	MSMS m/z	Error
130.0536	EGGE	E	[b1]1+	130.05	0.004
130.0536	EGEG	E	[b1]1+	130.05	0.004
130.0536	EEGG	E	[b1]1+	130.05	0.004
187.0693	GEGE	GE	[b2]1+	187.071	-0.002
187.0693	GEEG	GE	[b2]1+	187.071	-0.002
187.0693	EGGE	EG	[b2]1+	187.071	-0.002
187.0693	EGEG	EG	[b2]1+	187.071	-0.002
205.0784	GEGE	GE	[y2]1+	205.082	-0.004
205.0784	GEEG	EG	[y2]1+	205.082	-0.004
205.0784	EGGE	GE	[y2]1+	205.082	-0.004
205.0784	EGEG	EG	[y2]1+	205.082	-0.004
244.1021	GGEE	GGE	[b3]1+	244.093	0.009
244.1021	GEGE	GEG	[b3]1+	244.093	0.009
244.1021	EGGE	EGG	[b3]1+	244.093	0.009
259.0765	EEGG	EE	[b2]1+	259.092	-0.015
262.108	EGGE	GGE	[y3]1+	262.103	0.005
262.108	EGEG	GEG	[y3]1+	262.103	0.005
262.108	EEGG	EGG	[y3]1+	262.103	0.005
316.0786	GEEG	GEE	[b3]1+	316.114	-0.035
316.0786	EGEG	EGE	[b3]1+	316.114	-0.035
316.0786	EEGG	EEG	[b3]1+	316.114	-0.035

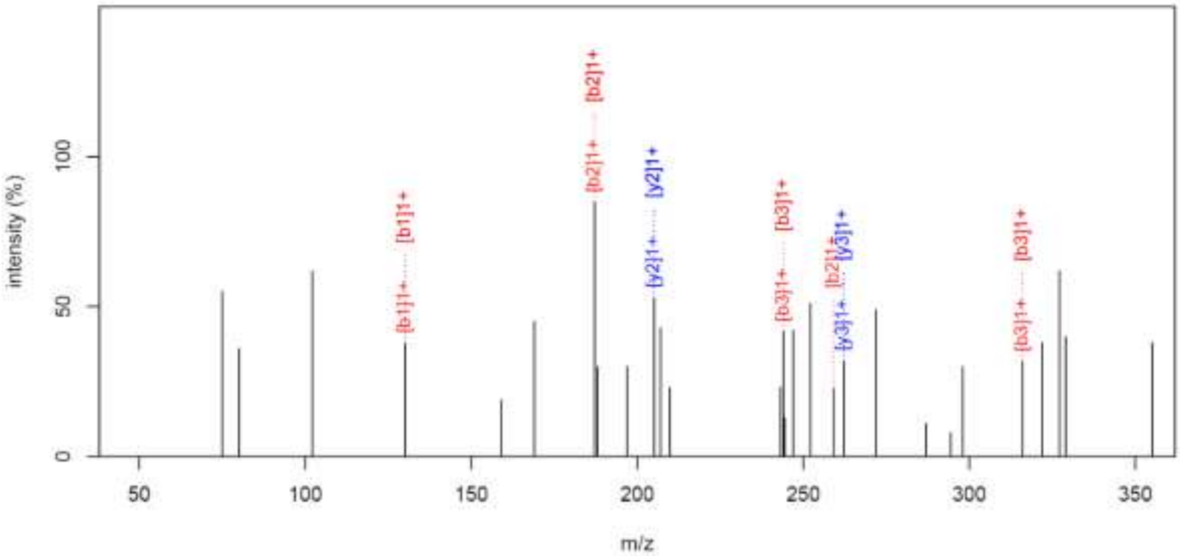

Supplementary Figure 54: Example MS/MS analyses of products of G and E monomer co-oligomerisation experiments. Spectrum (above) shows all the product ions resulting from MS/MS analysis of m/z = 391.146 (corresponding to a tetramer incorporating 2 x G and 2 x E). A series of fragments are observed which can be assigned as those expected (below) from a number of different possible sequences which might be expected from tetramers incorporating 2 x G and 2 x E (ie. more than one sequence of the same composition is present, all incorporating G and E).

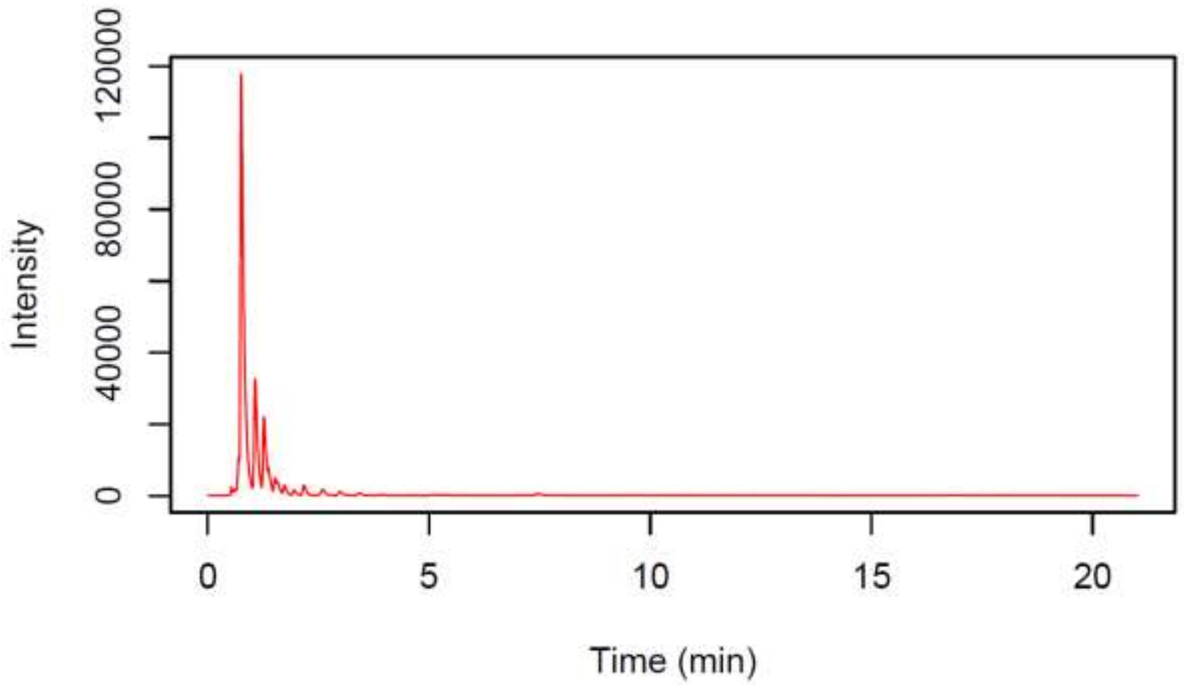

Supplementary Figure 55: "Virtual" BPC traces illustrating co-condensation of G and K monomers. VBPC is constructed from the EICs observed for a list of putative oligomers incorporating both of the amino acids monomers.

Experimental Product ion m/z	Possible parent sequence(s) at m/z=363.115	Possible production sequence(s)	Fragment type	MSMS m/z	Error
129.0966	KKGG	K	[b1]1+	129.102	-0.005
129.0966	KGKG	K	[b1]1+	129.102	-0.005
129.0966	KGGK	K	[b1]1+	129.102	-0.005
147.1163	KGGK	K	[y1]1+	147.113	0.003
147.1163	GKGK	K	[y1]1+	147.113	0.003
147.1163	GGKK	K	[y1]1+	147.113	0.003
186.105	KGKG	KG	[b2]1+	186.124	-0.019
186.105	KGGK	KG	[b2]1+	186.124	-0.019
186.105	GKKG	GK	[b2]1+	186.124	-0.019
186.105	GKGK	GK	[b2]1+	186.124	-0.019
204.1326	KGKG	KG	[y2]1+	204.134	-0.001
204.1326	KGGK	GK	[y2]1+	204.134	-0.001
204.1326	GKKG	KG	[y2]1+	204.134	-0.001
204.1326	GKGK	GK	[y2]1+	204.134	-0.001
243.1491	KGGK	KGG	[b3]1+	243.145	0.004
243.1491	GKGK	GKG	[b3]1+	243.145	0.004
243.1491	GGKK	GGK	[b3]1+	243.145	0.004
257.1957	KKGG	KK	[b2]1+	257.197	-0.001
261.1581	KKGG	KGG	[y3]1+	261.156	0.002
261.1581	KGKG	GKG	[y3]1+	261.156	0.002
261.1581	KGGK	GGK	[y3]1+	261.156	0.002
275.2036	GGKK	KK	[y2]1+	275.208	-0.004
332.2238	GKKG	KKG	[y3]1+	332.229	-0.005
332.2238	GKGK	KGK	[y3]1+	332.229	-0.005
332.2238	GGKK	GKK	[y3]1+	332.229	-0.005

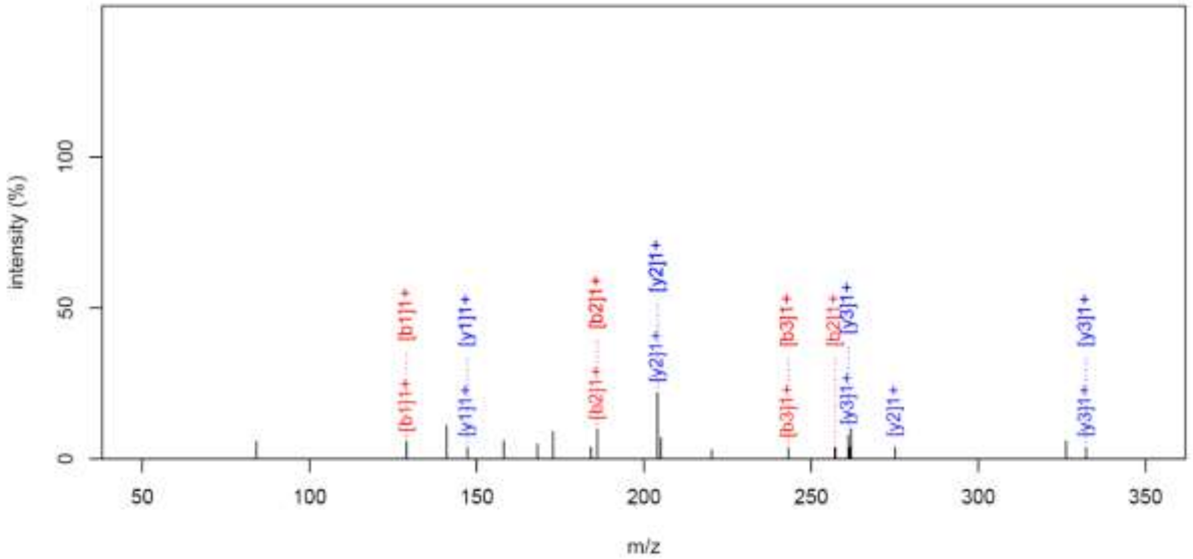

Supplementary Figure 56: Example MS/MS analyses of products of G and K monomer co-oligomerisation experiments. Spectrum (above) shows all the product ions resulting from MS/MS analysis of m/z = 389.251 (corresponding to a tetramer incorporating 2 x G and 2 x K). A series of fragments are observed which can be assigned as those expected (below) from a number of different possible sequences which might be expected from tetramers incorporating 2 x G and 2 x K (ie. more than one sequence of the same composition is present, all incorporating G and K).

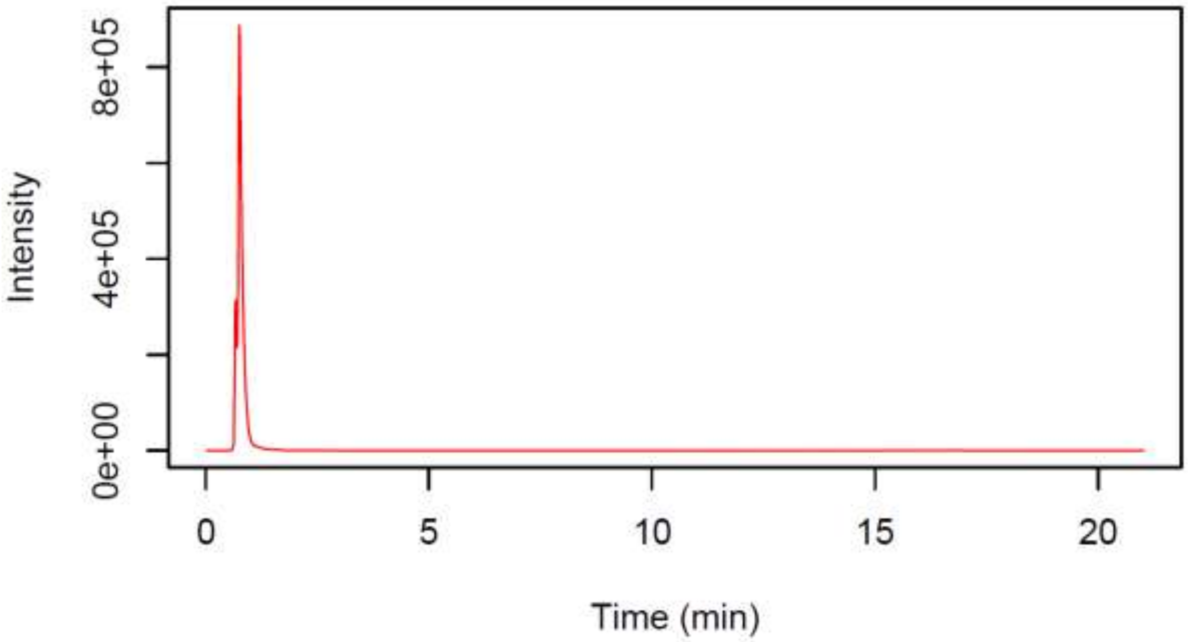

Supplementary Figure 57: “Virtual” BPC traces illustrating co-condensation of G and H monomers. VBPC is constructed from the EICs observed for a list of putative oligomers incorporating both of the amino acids monomers.

Experimental Product ion m/z	Possible parent sequence(s) at m/z=363.115	Possible production sequence(s)	Fragment type	MSMS m/z	Error
156.0753	HGGH	H	[y1]1+	156.077	-0.002
156.0753	GHGH	H	[y1]1+	156.077	-0.002
156.0753	GGHH	H	[y1]1+	156.077	-0.002
195.0851	HGHG	HG	[b2]1+	195.088	-0.003
195.0851	HGGH	HG	[b2]1+	195.088	-0.003
195.0851	GHHG	GH	[b2]1+	195.088	-0.003
195.0851	GHGH	GH	[b2]1+	195.088	-0.003
213.095	HGHG	HG	[y2]1+	213.098	-0.003
213.095	HGGH	GH	[y2]1+	213.098	-0.003
213.095	GHHG	HG	[y2]1+	213.098	-0.003
213.095	GHGH	GH	[y2]1+	213.098	-0.003
252.1054	HGGH	HGG	[b3]1+	252.109	-0.004
252.1054	GHGH	GHG	[b3]1+	252.109	-0.004
252.1054	GGHH	GGH	[b3]1+	252.109	-0.004
270.1143	HHGG	HGG	[y3]1+	270.12	-0.006
270.1143	HGHG	GHG	[y3]1+	270.12	-0.006
270.1143	HGGH	GGH	[y3]1+	270.12	-0.006
293.1282	GGHH	HH	[y2]1+	293.136	-0.008
350.1525	GHHG	HHG	[y3]1+	350.157	-0.005
350.1525	GHGH	HGH	[y3]1+	350.157	-0.005
350.1525	GGHH	GHH	[y3]1+	350.157	-0.005

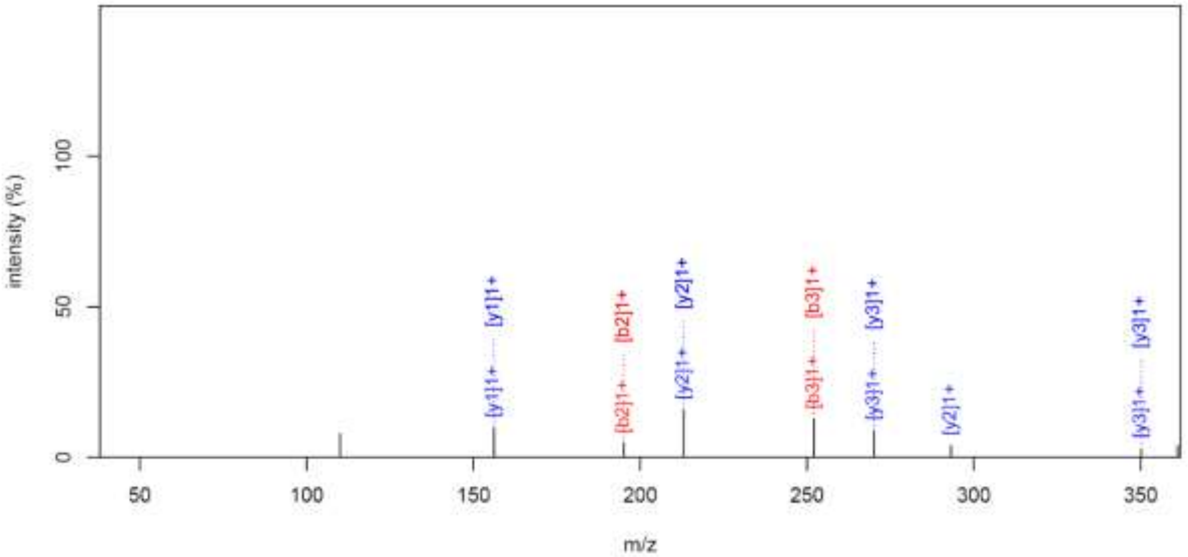

Supplementary Figure 58: Example MS/MS analyses of products of G and H monomer co-oligomerisation experiments. Spectrum (above) shows all the product ions resulting from MS/MS analysis of m/z = 407.179 (corresponding to a tetramer incorporating 2 x G and 2x H). A series of fragments are observed which can be assigned as those expected (below) from a number of different possible sequences which might be expected from tetramers incorporating 2 x G and 2 x H (ie. more than one sequence of the same composition is present, all incorporating G and H).

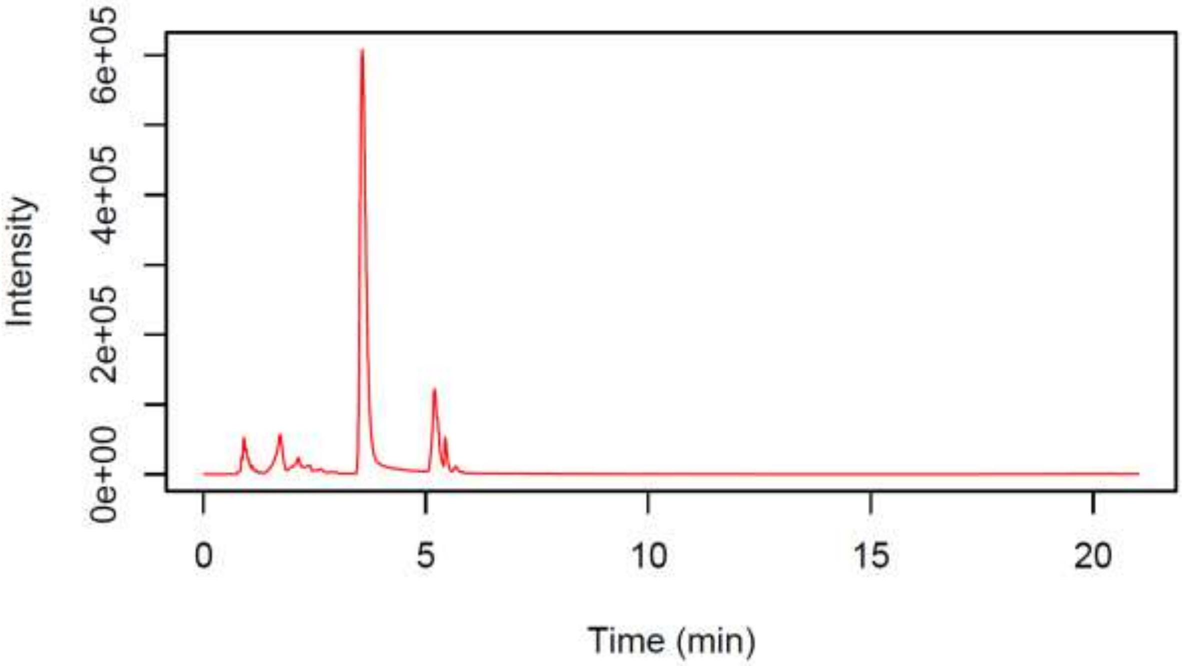

Supplementary Figure 59: “Virtual” BPC traces illustrating co-condensation of G and P monomers. VBPC is constructed from the EICs observed for a list of putative oligomers incorporating both of the amino acids monomers.

Experimental Product ion m/z	Possible parent sequence(s) at m/z=363.115	Possible production sequence(s)	Fragment type	MSMS m/z	Error
116.0691	PGGP	P	[y1]1+	116.071	-0.002
116.0691	GPGP	P	[y1]1+	116.071	-0.002
116.0691	GGPP	P	[y1]1+	116.071	-0.002
155.0795	PGPG	PG	[b2]1+	155.082	-0.002
155.0795	PGGP	PG	[b2]1+	155.082	-0.002
155.0795	GPPG	GP	[b2]1+	155.082	-0.002
155.0795	GPGP	GP	[b2]1+	155.082	-0.002
173.0896	PGPG	PG	[y2]1+	173.092	-0.002
173.0896	PGGP	GP	[y2]1+	173.092	-0.002
173.0896	GPPG	PG	[y2]1+	173.092	-0.002
173.0896	GPGP	GP	[y2]1+	173.092	-0.002
212.1	PGGP	PGG	[b3]1+	212.103	-0.003
212.1	GPGP	GPG	[b3]1+	212.103	-0.003
212.1	GGPP	GGP	[b3]1+	212.103	-0.003
230.1104	PPGG	PGG	[y3]1+	230.114	-0.004
230.1104	PGPG	GPG	[y3]1+	230.114	-0.004
230.1104	PGGP	GGP	[y3]1+	230.114	-0.004

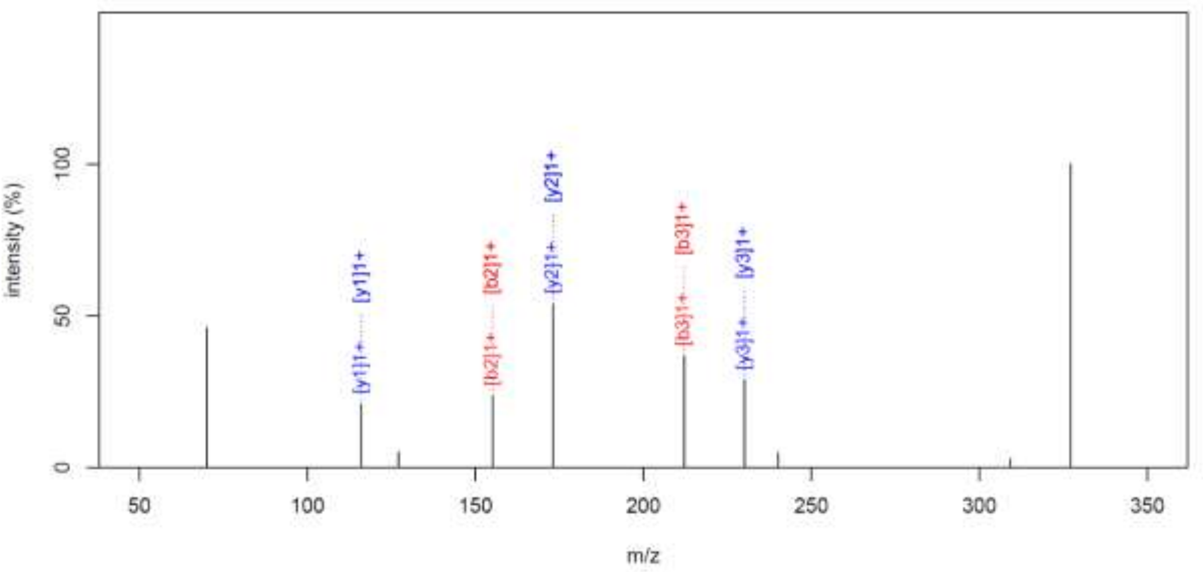

Supplementary Figure 60: Example MS/MS analyses of products of G and P monomer co-oligomerisation experiments. Spectrum (above) shows all the product ions resulting from MS/MS analysis of m/z = 327.166 (corresponding to a tetramer incorporating 2 x G and 2 x P). A series of fragments are observed which can be assigned as those expected (below) from a number of different possible sequences which might be expected from tetramers incorporating 2 x G and 2 x P (ie. more than one sequence of the same composition is present, all incorporating G and P).

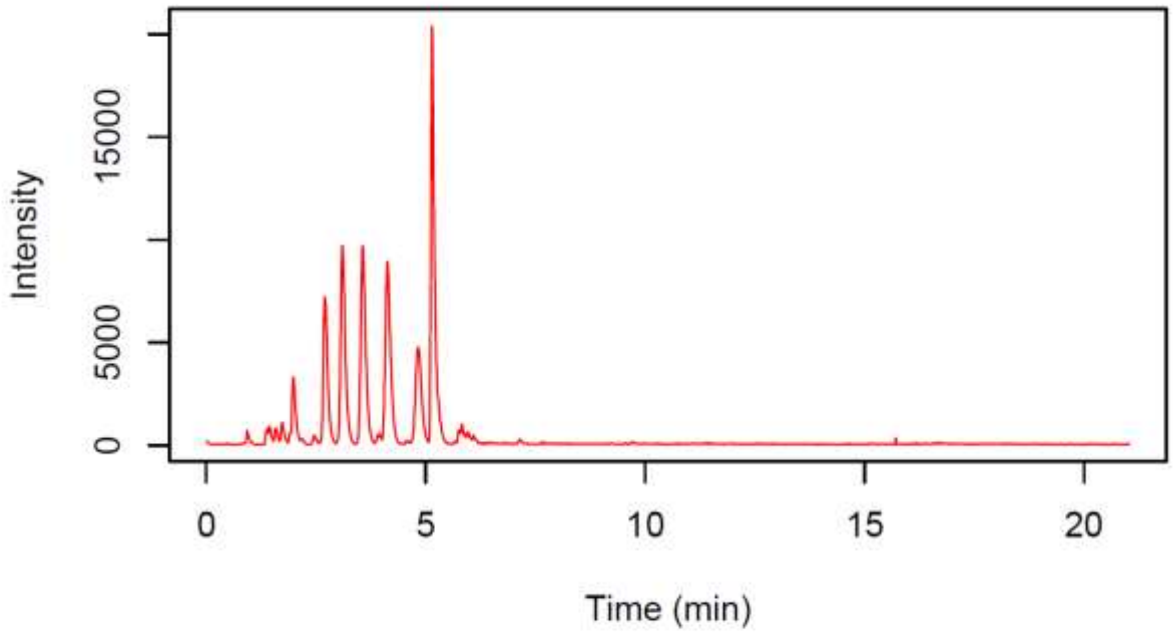

Supplementary Figure 61: “Virtual” BPC traces illustrating co-condensation of G and V monomers. VBPC is constructed from the EICs observed for a list of putative oligomers incorporating both of the amino acids monomers.

Experimental Product ion m/z	Possible parent sequence(s) at m/z=363.115	Possible production sequence(s)	Fragment type	MSMS m/z	Error
115.0521	GGVV	GG	[b2]1+	115.05	0.002
118.0793	VGGV	V	[y1]1+	118.086	-0.007
118.0793	GVGV	V	[y1]1+	118.086	-0.007
118.0793	GGVV	V	[y1]1+	118.086	-0.007
157.0938	VGVG	VG	[b2]1+	157.097	-0.003
157.0938	VGGV	VG	[b2]1+	157.097	-0.003
157.0938	GVVG	GV	[b2]1+	157.097	-0.003
157.0938	GVGV	GV	[b2]1+	157.097	-0.003
175.1017	VGVG	VG	[y2]1+	175.108	-0.006
175.1017	VGGV	GV	[y2]1+	175.108	-0.006
175.1017	GVVG	VG	[y2]1+	175.108	-0.006
175.1017	GVGV	GV	[y2]1+	175.108	-0.006
214.119	VGGV	VGG	[b3]1+	214.119	0
214.119	GVGV	GVG	[b3]1+	214.119	0
214.119	GGVV	GGV	[b3]1+	214.119	0
232.1239	VVGG	VGG	[y3]1+	232.129	-0.005
232.1239	VGVG	GVG	[y3]1+	232.129	-0.005
232.1239	VGGV	GGV	[y3]1+	232.129	-0.005

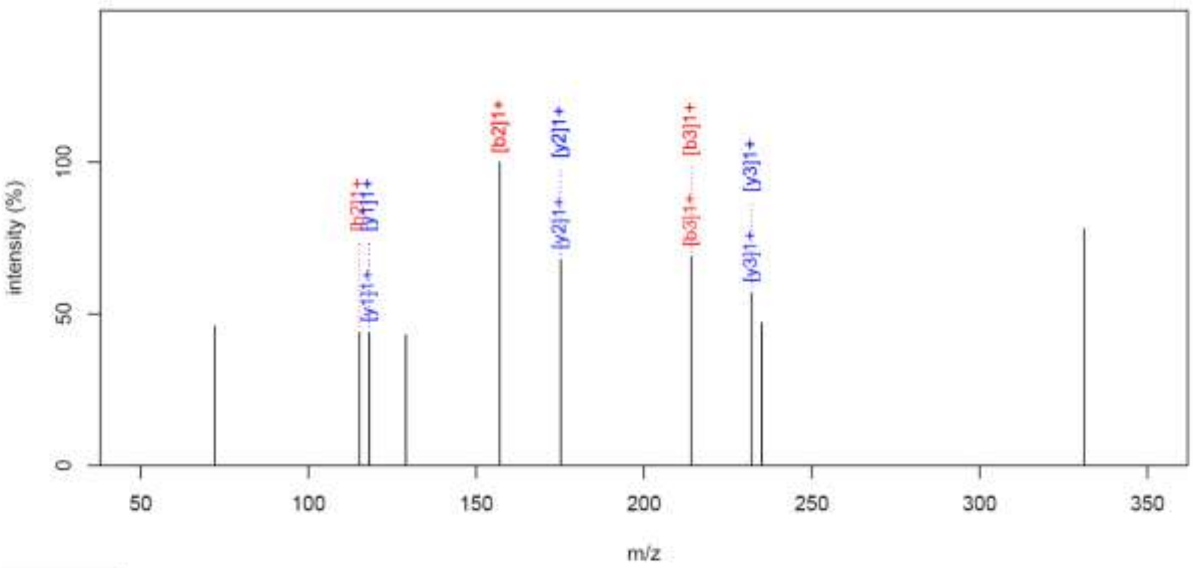

Supplementary Figure 62: Example MS/MS analyses of products of G and V monomer co-oligomerisation experiments. Spectrum (above) shows all the product ions resulting from MS/MS analysis of m/z = 331.198 (corresponding to a tetramer incorporating 2 x G and 2x V). A series of fragments are observed which can be assigned as those expected (below) from a number of different possible sequences which might be expected from tetramers incorporating 2 x G and 2 x V (ie. more than one sequence of the same composition is present, all incorporating G and V).

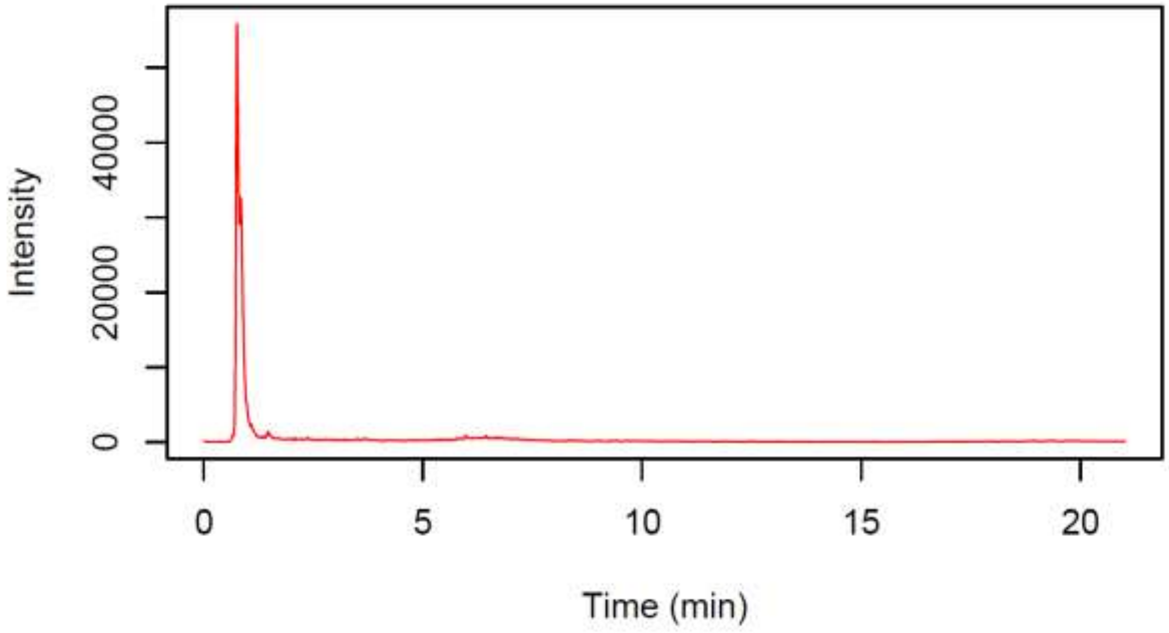

Supplementary Figure 63: “Virtual” BPC traces illustrating co-condensation of G and T monomers. VBPC is constructed from the EICs observed for a list of putative oligomers incorporating both of the amino acids monomers.

Experimental Product ion m/z	Possible parent sequence(s) at m/z=363.115	Possible production sequence(s)	Fragment type	MSMS m/z	Error
102.055	TTGG	T	[b1]1+	102.055	0
102.055	TGTG	T	[b1]1+	102.055	0
102.055	TGGT	T	[b1]1+	102.055	0
115.0464	GGTT	GG	[b2]1+	115.05	-0.004
115.0853	GGTT	GG	[b2]1+	115.05	0.035
120.0686	TGGT	T	[y1]1+	120.066	0.003
120.0686	GTGT	T	[y1]1+	120.066	0.003
120.0686	GGTT	T	[y1]1+	120.066	0.003
133.0587	TTGG	GG	[y2]1+	133.061	-0.002
159.0758	TGTG	TG	[b2]1+	159.076	0
159.0758	TGGT	TG	[b2]1+	159.076	0
159.0758	GTTG	GT	[b2]1+	159.076	0
159.0758	GTGT	GT	[b2]1+	159.076	0
177.0841	TGTG	TG	[y2]1+	177.087	-0.003
177.0841	TGGT	GT	[y2]1+	177.087	-0.003
177.0841	GTTG	TG	[y2]1+	177.087	-0.003
177.0841	GTGT	GT	[y2]1+	177.087	-0.003
203.099	TTGG	TT	[b2]1+	203.103	-0.004
216.0929	TGGT	TGG	[b3]1+	216.098	-0.005
216.0929	GTGT	GTG	[b3]1+	216.098	-0.005
216.0929	GGTT	GGT	[b3]1+	216.098	-0.005
221.1111	GGTT	TT	[y2]1+	221.113	-0.002
234.1073	TTGG	TGG	[y3]1+	234.108	-0.001
234.1073	TGTG	GTG	[y3]1+	234.108	-0.001
234.1073	TGGT	GGT	[y3]1+	234.108	-0.001
260.131	TTGG	TTG	[b3]1+	260.124	0.007
260.131	TGTG	TGT	[b3]1+	260.124	0.007
260.131	GTTG	GTT	[b3]1+	260.124	0.007

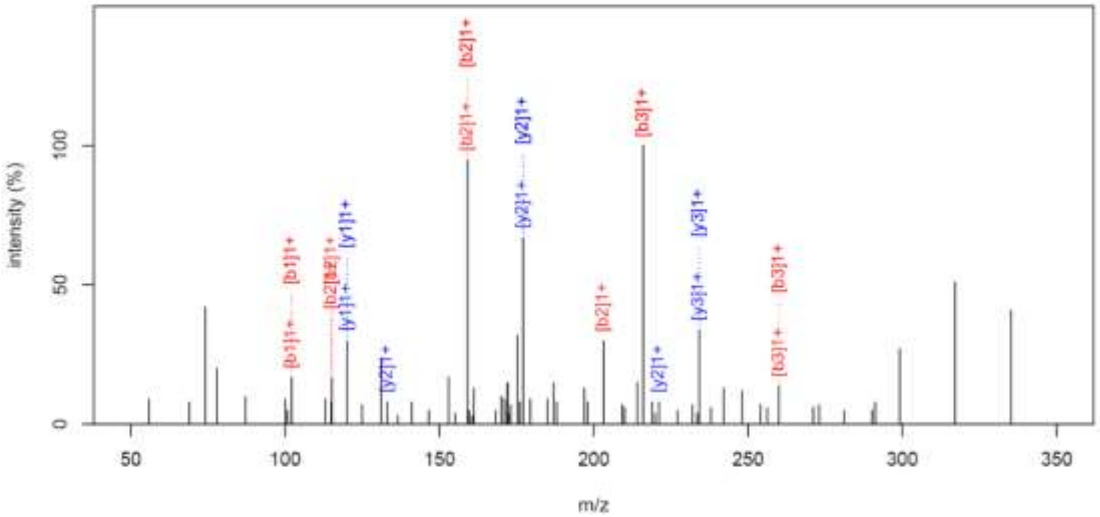

Supplementary Figure 64: Example MS/MS analyses of products of G and T monomer co-oligomerisation experiments. Spectrum (above) shows all the product ions resulting from MS/MS analysis of m/z = 335.156(corresponding to a tetramer incorporating 2 x G and 2 x T). A series of fragments are observed which can be assigned as those expected (below) from a number of different possible sequences which might be expected from tetramers incorporating 2 x G and 2 x T (ie. more than one sequence of the same composition is present, all incorporating G and T).

a

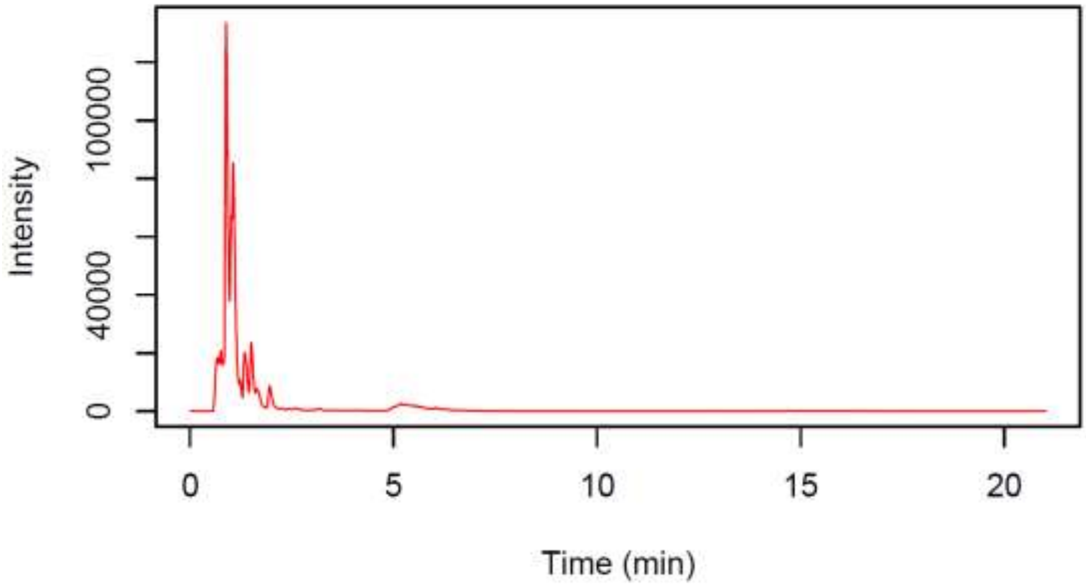

b

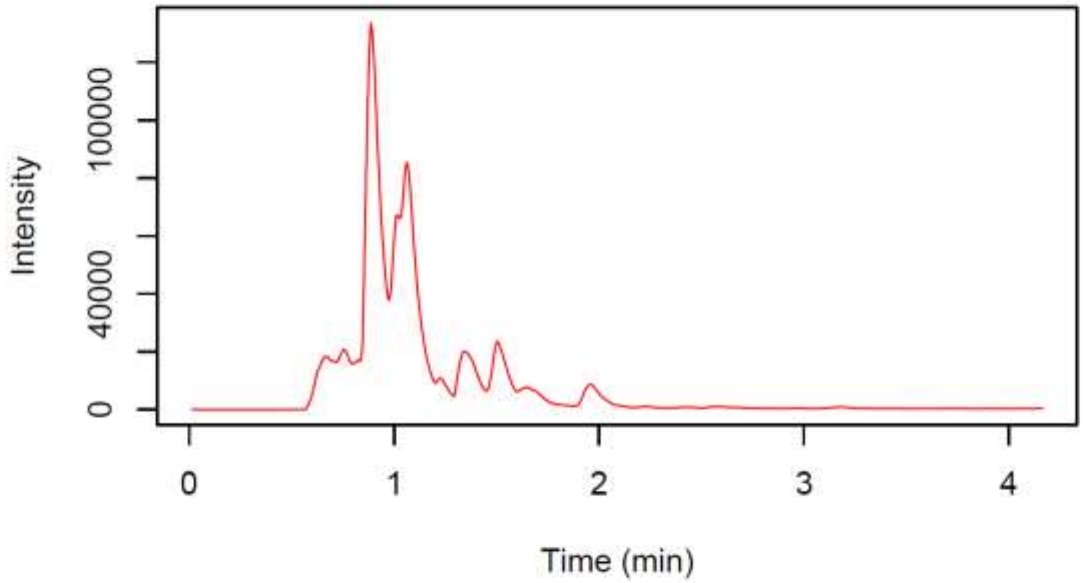

Supplementary Figure 65: "Virtual" BPC traces illustrating co-condensation of G, A and K monomers. VBPC is constructed from the EICs observed for a list of putative oligomers incorporating all three of the amino acids monomers. A) shows the entire VBPC spectra and B) focuses on the region of the spectra where peaks from the co-condensation products are easily observed.

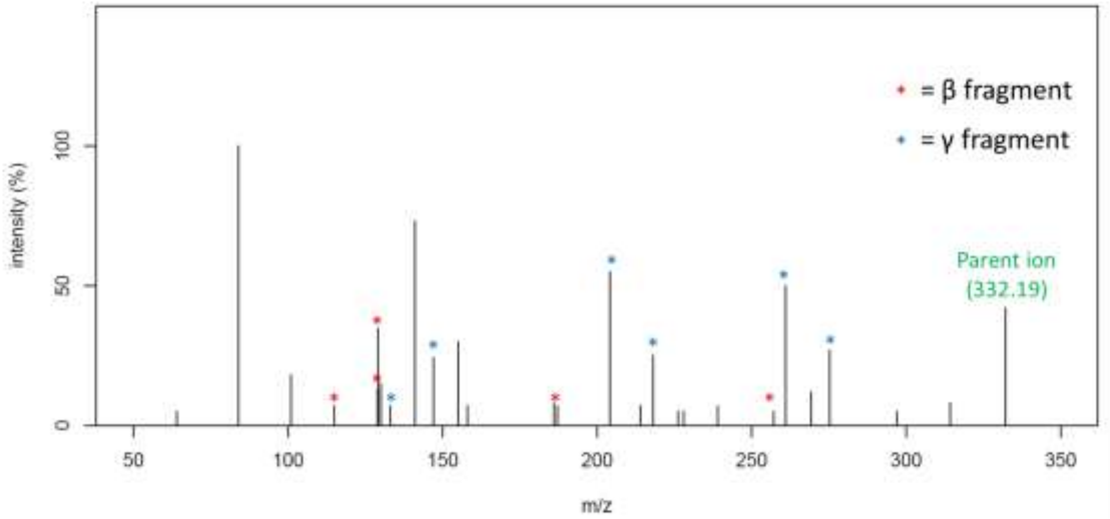

Experimental product ion m/z	Possible parent ion sequence(s) at m/z = 332.09	Possible product ion sequence(s)	Fragment type	MSMS m/z
115.0491	GGKA	GG	[b2]1+	115.05
115.0491	GGAK	GG	[b2]1+	115.05
129.0655	GAKG	GA	[b2]1+	129.066
129.0655	GAGK	GA	[b2]1+	129.066
129.0655	AGKG	AG	[b2]1+	129.066
129.0655	AGGK	AG	[b2]1+	129.066
129.1008	KGGA	K	[b1]1+	129.102
129.1008	KGAG	K	[b1]1+	129.102
129.1008	KAGG	K	[b1]1+	129.102
133.0597	KAGG	GG	[y2]1+	133.061
133.0597	AKGG	GG	[y2]1+	133.061
147.1115	KGGA	GA	[y2]1+	147.076
147.1115	KGAG	AG	[y2]1+	147.076
147.1115	GKGA	GA	[y2]1+	147.076
147.1115	GKAG	AG	[y2]1+	147.076
147.1115	GGAK	K	[y1]1+	147.113
147.1115	GAGK	K	[y1]1+	147.113
147.1115	AGGK	K	[y1]1+	147.113
186.0866	GGAK	GGA	[b3]1+	186.087
186.0866	GAGK	GAG	[b3]1+	186.087
186.0866	AGGK	AGG	[b3]1+	186.087
186.1216	KGGA	KG	[b2]1+	186.124
186.1216	KGAG	KG	[b2]1+	186.124

Experimental product ion m/z	Possible parent ion sequence(s) at m/z = 332.09	Possible product ion sequence(s)	Fragment type	MSMS m/z
186.1216	GKGA	GK	[b2]1+	186.124
186.1216	GKAG	GK	[b2]1+	186.124
204.1317	GAKG	KG	[y2]1+	204.134
204.1317	GAGK	GK	[y2]1+	204.134
204.1317	AGKG	KG	[y2]1+	204.134
204.1317	AGGK	GK	[y2]1+	204.134
218.1468	GGKA	KA	[y2]1+	218.15
218.1468	GGAK	AK	[y2]1+	218.15
257.159	KGAG	KGA	[b3]1+	257.161
257.159	KAGG	KAG	[b3]1+	257.161
257.159	GKAG	GKA	[b3]1+	257.161
257.159	GAKG	GAK	[b3]1+	257.161
257.159	AKGG	AKG	[b3]1+	257.161
257.159	AGKG	AGK	[b3]1+	257.161
261.1533	AKGG	KGG	[y3]1+	261.156
261.1533	AGKG	GKG	[y3]1+	261.156
261.1533	AGGK	GGK	[y3]1+	261.156
275.1684	GKGA	KGA	[y3]1+	275.171
275.1684	GKAG	KAG	[y3]1+	275.171
275.1684	GGKA	GKA	[y3]1+	275.171
275.1684	GGAK	GAK	[y3]1+	275.171
275.1684	GAKG	AKG	[y3]1+	275.171
275.1684	GAGK	AGK	[y3]1+	275.171

Supplementary Figure 66: Example MS/MS analyses of products of G, A, and K monomer co-oligomerisation experiments. Spectrum (above) shows all the product ions resulting from MS/MS analysis of m/z = 332.09 (corresponding to a tetramer incorporating 2 x G, 1 x A, and 1 x K) eluted over t = 0.5 – 2 min in RP-HPLC-MS. A series of fragments are observed which can be assigned as those expected (below) from a number of different possible sequences which might be expected from tetramers incorporating 2 x G, 1 x A, and 1 x K (i.e. more than one sequence of the same composition is present, all incorporating G, A, and K).

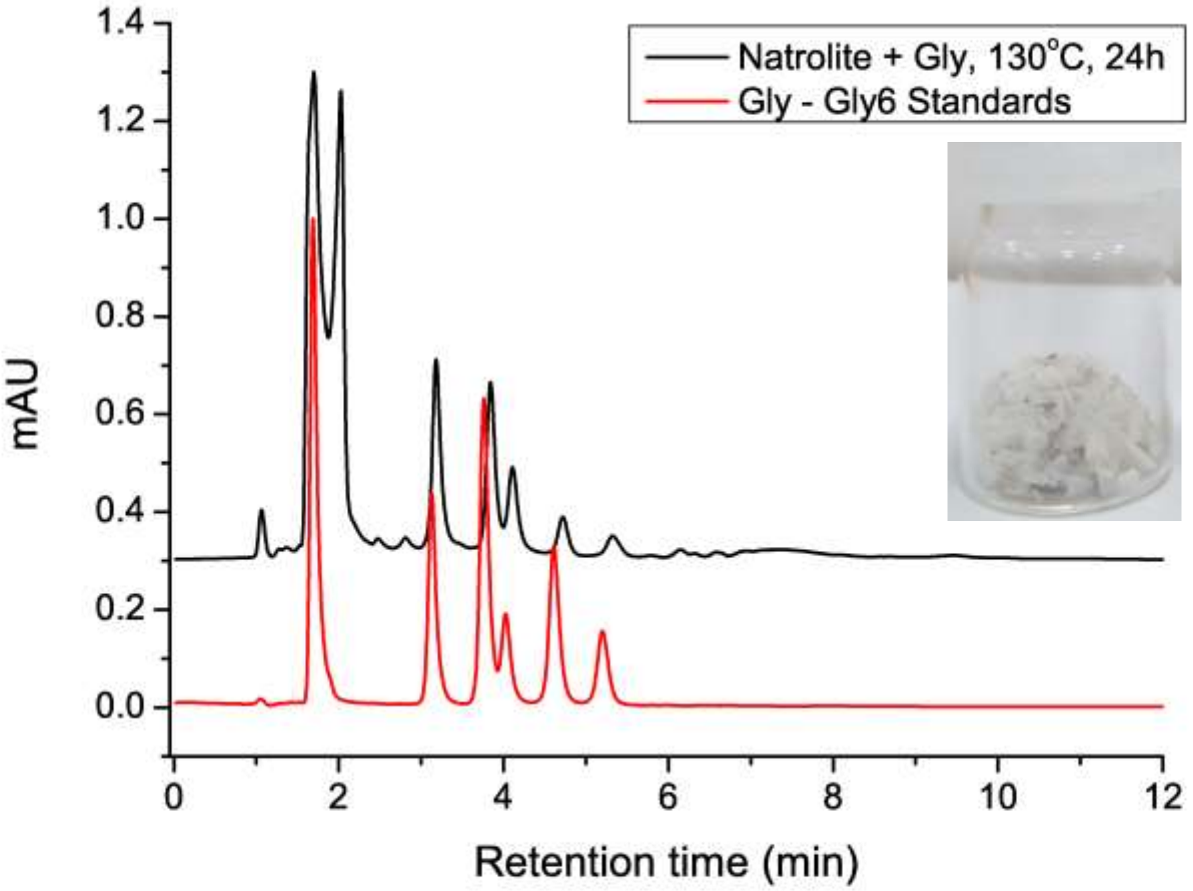

Supplementary Figure 67: IP-HPLC chromatogram of glycine oligomerisation products with natrolite mineral (24h, 130ºC) compared to Gly, $(Gly)_{2-6}$ standards. Inset: a vial containing natrolite.

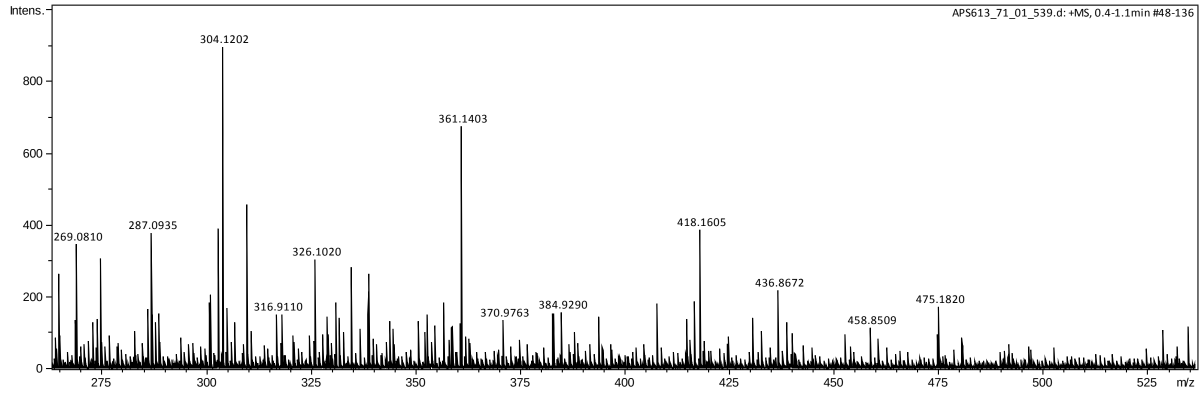

Supplementary Figure 68: MS of integration of the 0.5-1.5 min area of the RP-HPLC-MS of the natrolite experiment, clearly showing a peak at 418.168 and 475.19 (7-mer and 8-mer, respectively). This can also be see in the EIC.

Supplementary Table 1: Table of calculated yields vs. dehydration time at 90ºC /110ºC / 130ºC at pH9.8.

Dehydration time (hours)	90ºC	Error(%)	110ºC	Error(%)	130ºC	Error(%)
**1**	0.07	7.87295822	0.05476007	21.36	0.0786	0.00
**3**	0.37	12.38534	2.262746011	3.75	17.20836179	1.52
**7**	0.65	19.01494	8.043332904	9.04	37.87169978	3.93
**15**	7.58	6.21074	15.81516302	9.35	42.51868225	4.23
**24**	7.8667	4.27378351	24.37700448	0.32	38.58585171	2.56
**48**	12.04	13.65692	34.96651851	0.36	35.07398746	3.10
**72**	18.4133	3.354	41.66696797	3.48	33.5822961	3.73
**96**	22.25667	0.922556	39.17051687	7.60	29.47759773	1.07

Supplementary Table 2: Table of calculated yields vs. pH at 130ºC after 24h.

pH	130ºC	Error(%)
2.15	19.80966425	8.498902
2.95	8.119637164	11.12281
3.90	1.554716719	7.308297
4.85	0.287135193	6.280165
6.10	0.797804131	11.23695
8.00	3.719450448	2.93288
8.67	6.047332728	5.277626
8.90	11.15483399	4.514116
9.30	21.13742904	4.045178
9.60	33.44756051	3.894415
9.80	38.56432132	4.599946
10.00	44.70615593	1.827024

Supplementary Table 3: Table of calculated oligomer concentrations vs. number of hydration-dehydration cycles at 130ºC after 24h. Yields calculated as a percentage of the Gly starting material accounted for each product.

[Oligomer] (mM)	Cycle 1	Cycle 2	Cycle 3	Cycle 4
**Gly_2**	13.96	10.26	9.42	8.36
**Gly_3**	10.40	7.70	6.46	5.41
**Gly_4**	7.61	5.95	5.11	4.41
**Gly_5**	5.11	4.23	3.53	3.07
**Gly_6**	3.64	3.71	3.37	3.03
**Gly_7**	1.91	2.07	1.94	1.67
**Gly_8**	1.91	2.05	1.07	0.64
**Gly_9**	1.09	1.30	0.77	0.66
**Gly_{10}**	0.81	0.93	0.81	0.74
**Gly_{11}**	0.20	0.32	0.28	0.26
**Gly_{12}**	0.40	0.56	0.40	0.38
**Gly_{13}**	0.11	0.34	0.30	0.25

Supplementary Table 4: Concentration of glycine oligomers formed against glycine monomer input concentration.

[Starting material] (M)	[Gly_2] (mM)	[Gly_3] (mM)
10^{-4}M	0.007	0.003
10^{-3}M	0.159	0.015
10^{-2}M	0.376	0.155
10^{-1}M	1.292	1.750

Supplementary Table 5: Table of amide N-H and C=O stretches. N-H stretch: secondary amide produces one N-H stretch at 3500-3100 cm^{-1}. C=O stretch: carboxyl groups in amide produces one C=O stretch at 1690-1630 cm^{-1}.

	IR AMIDE SIGNAL	
**Sample/standard**	**N-H stretch (cm^{-1})**	**C=O stretch (cm^{-1})**
Gly_2 – Gly_6 standards	3307	1634
Reaction carried out without NaCl addition	3271	1640
Reaction carried out with NaCl addition	3871	1640

Supplementary Table 6: Biuret assay data processing.

	Sample	Abs	Abs - 0	conc (G5)	conc (G6)	conc (avg)
**Blank**	**0**	0.0275	**0**	0	0	**0**
**Pentaglycine**	**G5-0.1mg/ml**	0.0669	**0.0394**	0.1044	0.1217	**0.1130**
	**G5-0.3mg/ml**	0.142	**0.1145**	0.3033	0.3536	**0.3284**
	**G5-0.5mg/ml**	0.2152	**0.1877**	0.4972	0.5796	**0.5384**
**Hexaglycine**	**G6-0.1mg/ml**	0.0596	**0.0321**	0.0850	0.0991	**0.0921**
	**G6-0.3mg/ml**	0.1193	**0.0918**	0.2432	0.2835	**0.2633**
	**G6-0.5mg/ml**	0.1927	**0.1652**	0.4376	0.5101	**0.4738**
**APS Samples (dehydration time)**	**15h**	0.0972	**0.0697**	0.1846	0.2152	**0.1999**
	**24h**	0.1519	**0.1244**	0.3295	0.3841	**0.3568**
	**72h**	0.2171	**0.1896**	0.5022	0.5854	**0.5438**

Supplementary Methods

LabView Control Software

The control over the fluids described in the manuscript is implemented with Tricontinent syringe pumps operated through a USB to Serial connection to a laptop (Dell Latitude 3440). We developed a user interface for simple control of the pumps as well as easier implementation of the experimental design through National Instruments LabVIEW 2013 SP1 (32 bit). Figures S3-S6 show screen shots of the main control software, "pump_control.vi," as well as an important Sub VI, "Pick up and Deliver (dist vale).vi". Commands are sent with the inbuilt VISA functions included with a LabVIEW installation. The Tricontinent C-Series Precision Pump (C3000/C24000) Software Manual and the C3000 Precision Pump Operator's Manual (available from the Tricontinent website, http://www.tricontinent.com) were used to inform the writing this control software (see Supplementary Figs 3-6).

HPLC analysis and characterisation of glycine homo-oligomers

Dehydration time dependence experiments

Starting from an aqueous solution of glycine monomer and keeping the reaction at pH=9.75, we show the conversion to oligomers depending on the dehydration time (after 1, 3, 5, 7, 15 and 24 h) at three different temperatures (90, 110 and 130 ºC). The highest conversion takes places after 15h.

In all experiments, Glycine (350µl from a 1M Gly solution, so $3.5 \cdot 10^{-4}$ moles) was mixed with 1 ml of a 1M solution of NaCl + 2.55 ml HPLC water at pH=9.75 (adjusted by adding 100 microliters of NaOH 1M) .

Each sample was dehydrated in a single cycle for different periods of time: 1, 3, 5, 7, 15, 24, 48, 72 and 96h. Once finished, products were prepared for analysis by dissolution in 8ml of a 0.1% TFA aqueous solution (see Supplementary Table 1 and Supplementary Figs 9 and 10).

pH dependence experiments

Starting from an aqueous solution of glycine monomer and keeping the reaction at the same temperature (130°C) for a dehydrating time of 24 hours, we show the conversion to oligomers depending on the pH (between pH=10.0 and 2.15).
In all experiments, Glycine (350µl from a 1M Gly solution, so $3.5\cdot10^{-4}$ moles) was mixed with 1 ml of a 1M solution of NaCl + 2.55 ml HPLC water at various pH values (adjusted by adding a 1M NaOH or 1M HCl solution) . Once finished, products were prepared for analysis by dissolution in 8ml of a 0.1% TFA aqueous solution (see Supplementary Table 2 and Supplementary Figs 11-13).

Cycle dependency experiments

Starting from an aqueous solution of glycine monomer and keeping the reaction at the same temperature (130°C) and pH= 2.61 for a dehydrating time of 24 hours, we show the conversion to oligomers depending on number of hydration-dehydration cycles (*n*=1-3).
In all experiments, Glycine (350µl from a 1M Gly solution, so $3.5\cdot10^{-4}$ moles) was mixed with 1 ml of a 1M solution of NaCl + 2.60 ml HPLC water, and adjusted to pH=2.61 by adding 50 µl of a 1M HCl solution.
Each sample was dehydrated and subsequently rehydrated for a given number of cycles. Once finished, products were prepared for analysis by dissolution in 8ml of a 0.1% TFA aqueous solution (see Supplementary Table 3 and Supplementary Fig 14).

Concentration dependency experiments

Starting from an aqueous solution of glycine monomer and keeping the reaction at the same temperature (130°C) and pH= 9.8 for a dehydrating time of 24 hours, we show the conversion to oligomers depending on the concentration of the starting material.
Four solutions of Glycine monomer were prepared at different concentrations (10^{-1}M, 10^{-2}M, 10^{-3}M and 10^{-4}M), and adjusted at pH 9.8 by using NaOH 1M. 350µl of each solution were added to 100µl of NaCl 1M + 500µl of HPLC water. The samples were dehydrated for 24h in

a single dehydration experiment. All the dried products were prepared for analysis by adding 1 ml of HPLC water (see Supplementary Table 4 and Supplementary Fig 15).

Glycinamide and DKP Oligomerisation Reactions

In this experiment, DKP (0.0014g) was dissolved in 1 ml of a 1M solution of NaCl + 2.55 ml HPLC water, and adjusted to pH=9.75 using 1M NaOH. The sample was left to dehydrate for 24h in a single cycle dehydration experiment. Once finished, products were prepared for analysis by dissolution in 8ml of a 0.1% TFA aqueous solution (see Supplementary Figures 16).

In this experiment, Glycinamide (0.0014g) was dissolved in 1 ml of a 1M solution of NaCl + 2.55 ml HPLC water, and adjusted to pH=9.75 using 1M NaOH. The sample was left to dehydrate for 24h in a single cycle dehydration experiment. Once finished, products were prepared for analysis by dissolution in 8ml of a 0.1% TFA aqueous solution (see Supplementary Fig 17).

Glycine oligomerisation in a Teflon® vessel

In this, Glycine (350µl from a 1M Gly solution) was mixed with 1 ml of a 1M solution of NaCl + 2.55 ml HPLC water, and adjusted to pH=9.75 using 1M NaOH. The sample was left to dehydrate for 24h in a single cycle dehydration experiment in a Teflon® vessel. Once finished, products were prepared for analysis by dissolution in 8ml of a 0.1% TFA aqueous solution (see Supplementary Fig 18).

Evidence for concurrent bond breakage and formation

In this experiment, Glycylglycine (175µl from a 1M GlyGly solution, so $1.75 \cdot 10^{-4}$ moles) was mixed with 1 ml of a 1M solution of NaCl + 2.725 ml HPLC water, and adjusted to pH=9.8 by adding 100 µl of a 1M NaOH solution. The reaction was left to dehydrate at a) 110 ºC and b) 130ºC for a single dehydration cycle. Once finished, products were prepared for analysis by dissolution in 8ml of a 0.1% TFA aqueous solution.

Since $(Gly)_n$ series are observed with odd-numbered n (i.e. n = 3,5, 7, etc), we can conclude that peptide bonds must have been both broken and formed in the same experimental cycle.

We observe a greater occurrence of this at higher temperatures (see Supplementary Figs 19 and 20).

NaCl concentration dependence

We show that conversion to oligomers does not depend strongly on the concentration of NaCl added to the starting solution, with a series of experiments.

Each experiment started from an aqueous solution of glycine monomer, and was maintained at 130°C and pH=9.75 for a dehydrating time of 15 hours.

In all experiments, Glycine (350μl from a 1M Gly solution, so $3.5 \cdot 10^{-4}$ moles) was diluted in a total volume of 4ml. Different volumes of a 5M NaCl solution were added to adjust the NaCl concentration to 0, 0.025, 0.25 and 1 M. The pH was adjusted to 9.75 by adding 100 μl of a 1M NaOH solution (except in one case, where LiOH was used, to demonstrate that Na^+ was not vital). Each sample was dehydrated for one cycle. Once finished, products were prepared for analysis by dissolution in 8ml of a 0.1% TFA aqueous solution (see Supplementary Figs 21-23).

MS/MS Analysis of glycine homo-oligomers product ions

The Supplementary Figures 24-28 include typical MS/MS spectra of $(Gly)_x$ oligopeptide products, clearly exhibiting fragmentation patterns consistent with their assigned structures, comprising multiple peptide bonds.

Direct infusion MS analysis of glycine homo-oligomers products

The sample was prepared by taking an aliquot of 350 μl from a 1 M solution of glycine, to which we subsequently added: 1 ml of a 1M NaCl solution, 2 ml of (HPLC) water, and finally the pH was adjusted to 9.75 by adding 200 μl of 1M NaOH. The sample was processed in our APS system for 1 hydration-dehydration cycle with a dehydrating time of 24h. After extracting the sample with 4ml of HPLC water, it was analysed by HPLC, and then freeze dried for ESI-MS analysis.

The sample was solvated in water and diluted into a mixture of Water:MeOH 1:1 with 10% formic prior to analysis by positive nanospray. The expected $(+H)^+$ series from *n*=2 to *n*=7 is clearly observed; further oligomers are obvious extending to higher mass as the Na^+ adducts . The sample also seems to be a mixture, with at least one other series evident with the repeat unit of 57Da. i.e. ions at 550/607/664/721 (778) (see Supplementary Figs 29-39).

MALDI analysis of a glycine homo-oligomers solid fraction

Sample was attempted by solvent-free MALDI, initially with CHCA (α-Cyano-4-hydroxycinnamic acid) matrix typically used for peptide samples. However, no polymeric species and only matrix ions were observed. The analysis was repeated with DHB (2,5-dihydroxybenzoic acid) matrix, and now in addition to matrix ions, 4 oligomeric series were observed. The four series all have the expected repeat unit mass of 57Da, and correspond to $[M+H]^+$, $[M+Na]^+$, $[M+K]^+$, and the MALDI artefact $[M-H+2Na]^+$; species for n=8 can be observed considering only–H and –OH endgroups. Despite these four series splitting the ion intensity, oligomeric species are observable up to around 1300Da, corresponding to at least $(Gly)_{20}$ (see Supplementary Fig 41).

IR, NMR and chemical tests of glycine homo-oligomers

Transmission IR Spectroscopy

The solid samples collected for IR analysis were obtained by the following experimental procedure:

Glycine (350µl from a 1M Gly solution, so $3.5 \cdot 10^{-4}$ moles) was diluted to a total final volume of 4 ml and the pH adjusted to 9.5. Two different experiments were simultaneously performed with (1ml of a 1M NaCl solution) and without NaCl. Both samples were left to dehydrate at 130 °C for 15h, in order to evaporate the solution to complete dryness. Once a cycle was finished, the process was re-started by re-hydrating the sample with 4 mL of HPLC water, for a total number of 10 dehydration-hydration cycles.

Once finished, insoluble oligomeric products were separated from the supernatant by centrifugation and decantation. The solid was further washed by re-suspension in HPLC

grade water and freeze-dried overnight (see Supplementary Table 5 and Supplementary Figs 42 and 43).

^{1}H-NMR Spectrometry

For NMR analysis, deuterated DMSO was added to a sample from one of the peptide formation experiments and the mixture heated to aid dissolution. The saturated DMSO was then filtered into an NMR tube and a proton spectrum was taken. Using similar methods, saturated standards of the glycine monomer, dimer and trimer were also prepared. Looking specifically at the α-proton region (ca. 3.5 to 5.5), we can see there is a strong similarity between the sample peaks and the peaks seen for the trimer standard (shown inset). Looking then at the amide proton region (ca. 7 to 11 ppm) for the sample and all standards, no peak could be observed for the monomer (as expected), while peaks could be observed for both the dimer and trimer. The sample showed a number of peaks in this region (as would be expected for a complex mixture of oligomers and side products), but peaks in the right regions for the dimer and trimer could be seen. This shows that the proton NMR is not at all inconsistent with the presence of amide bonds (see Supplementary Figs 44 and 45).

Qualitative chemical testing for peptide bonds

Qualitative chemical testing for peptide bonds was performed using the Biuret test. 1ml of Biuret reagent (hydrated copper (II) sulfate, sodium hydroxide (NaOH) and potassium sodium tartrate.) was added to a solution of ~5mg amino acid/polyamino acid in 1ml of fresh 0.1M sodium hydroxide solution. A positive test for peptide bonds is shown by a colour change from pale blue to violet. As can be seen from the Supplementary Fig 46, Gly and Gly_2 are pale blue, whilst a standard solution of Gly_3 shows a pale violet colour, indicating that at least three amino acids residues are required for a positive test. Oligomeric products resulting from APS samples obtained after 1 and 3 dehydration cycles show positive violet colouration.

Quantitative Biuret assay

Standard solutions of Gly_5 and Gly_6 were prepared in 0.1M NaOH at a concentration of 1 mg/ml. Further dilution steps were performed to achieve the following standard concentrations: 0.1mg/ml, 0.3mg/ml and 0.5mg/ml. Then they were diluted to a total volume of 0.5ml with 0.1M NaOH. APS samples were prepared using 0.1ml of sample dissolved in 0.4ml 0.1M NaOH. Then 0.5ml of Biuret reagent was added to the sample/standard diluted in 0.1M NaOH. Finally the solutions were shaken to ensure complete mixing and reaction.

The absorption was then measured at 540nm. A background chromatogram was taken of 0.5ml Biuret reagent in 0.5ml 0.1M NaOH (see Supplementary Table 6 and Supplementary Figs 47 and 48).

Experiment using natrolite mineral and glycine monomer

Natrolite is a tectosilicate mineral species belonging to the zeolite group. It is a basic hydrated sodium and aluminium silicate with the formula $Na_2Al_2Si_3O_{10}\cdot 2H_2O$. Natrolite was obtained from Richard Tayler Minerals, Cobham, Surrey, England, and was used (crushed) without further purification.

In this experiment, Glycine (350μl from a 1M Gly solution, so $3.5\cdot 10^{-4}$ moles) was dissolved in 4 ml water and added to ca. 500mg crushed natrolite. The sample was dehydrated in a single cycle for 24h at 130ºC. All the dried products were extracted by adding 4 ml of HPLC water (see Supplementary Figs 49 and 50).

Hetero-oligomer synthesis

In all experiments, Glycine (3.5ml from a 0.1M Gly solution, so $3.5\bullet 10^{-4}$ moles) was mixed with 3.5ml of a 0.1M solution of a second amino acid (A, D, E, K, H, P, V and T). Then, 1 ml of a 1M solution of NaCl was added, and the pH was adjusted to pH = 3 using a small amount of 1M HCl solution. All samples were dehydrated at 130°C for 15h; they were rehydrated (4 ml of HPLC water) and dehydrated (again, 130°C for 15h) to achieve a total number of 5 dehydration-hydration cycles. Once finished, products were prepared for analysis by dissolution in 8ml of a 0.1% TFA aqueous solution.

Confirmation of the production of hetero-oligomers incorporating different amino acids

Since increased complexity results from the co-condensation of multiple amino acid monomers and makes thorough characterization of all the resulting products (presuming a relatively uncontrolled/non-selective reaction) more challenging, a “virtual” BPC, constructed. This is derived from EICs resulting from a combinatorial list of the all possible oligomer-forming combinations of both/all the amino acids (i.e. only species incorporating all starting amino acids are represented, not homo-oligomers. In all cases many peaks are observed in these traces, suggesting the formation of many co-oligomerisation products.

Secondly, some representative components of these complex mixtures were chosen for MS/MS analysis to corroborate the peptide nature of the products observed (see Supplementary Figs 51-68).

Supplementary References

1 Liao, W. L., Heo, G. Y., Dodder, N. G., Pikuleva, I. A., Turko, I. V. Optimizing the conditions of a multiple reaction monitoring assay for membrane proteins: quantification of cytochrome P450 11A1 and adrenodoxin reductase in bovine adrenal cortex and retina. *Anal. Chem.* **82**, 5760-5767 (2010).

2 R Development Core Team (2008). R: A language and environment for statistical computing. R Foundation for Statistical Computing, Vienna, Austria. *ISBN 3-900051-07-0*, URL http://www.R-project.org.

3 Chambers, M. C. *et al.* A cross-platform toolkit for mass spectrometry and proteomics. *Nature Biotech.* **30**, 918-920 (2012).

4 Smith, C. A., Want, E. J., O'Maille, G., Abagyan, R., Siuzdak, G. XCMS: Processing mass spectrometry data for metabolite profiling using nonlinear peak alignment, matching and identification. *Anal. Chem.* **78**, 779-787 (2006).
